# A role for selective ER autophagy in gametogenic rejuvenation revealed by high-throughput lifespan profiling

**DOI:** 10.1038/s41467-026-77993-0

**Published:** 2026-09-29

**Authors:** Silvan Spiri, Tina Lynn Sing, Nhi Phung, Jay S. Goodman, Elçin Ünal, Gloria Ann Brar

**Affiliations:** 1 Department of Molecular and Cell Biology, University of California, Berkeley, Berkeley, CA USA; 2 California Institute for Quantitative Biosciences (QB3), University of California, Berkeley, Berkeley, CA USA; 3 Center for Computational Biology, University of California, Berkeley, Berkeley, CA USA

**Keywords:** Autophagy, Ageing, Endoplasmic reticulum, Lab-on-a-chip, Meiosis

## Abstract

During mitotic growth, *Saccharomyces cerevisiae* cells age by dividing asymmetrically, producing young daughter cells while retaining age-associated damage in the mother cell, which will eventually become senescent. Gametogenesis naturally and fully resets precursor cell lifespan, even for replicatively aged cells. However, the mechanisms responsible for gametogenic rejuvenation have remained mysterious. This is partly due to the existing methods to quantify lifespan resetting in this context, which are limited to low-throughput and labor-intensive approaches. Here, we introduce a high-throughput assay that allows systematic characterization of factors required for gametogenic rejuvenation. With this approach, we show that we can sensitively measure a wide range of gamete replicative lifespans that are consistent with established mutants. Excitingly, we also discover that Atg39 and Atg40, receptors involved in selective autophagy of the ER, are molecular determinants of gametogenic rejuvenation. This technique will enable systematic identification of the molecular factors that drive gametogenic rejuvenation.

## Abstract (web summary)

Gametogenesis fully resets lifespan, although how this is achieved is unclear. Here, Spiri et al. developed a high-throughput assay to study this rejuvenation event in yeast, revealing selective autophagy of the ER as a key molecular mechanism.

## Introduction

Life is continuously maintained through the genesis of offspring. In sexually reproducing eukaryotes, gametogenesis is the conserved developmental program that forms haploid gametes from diploid precursor cells. Although somatic cells deteriorate over time, robust mechanisms eliminate age-associated damage in the germline and during gametogenesis to secure the fitness and survival of the next generation^1–7^. Despite the importance of these pathways, the specific molecular mechanisms required for mediating this programmed developmental rejuvenation remain elusive.

**Fig. 1:**
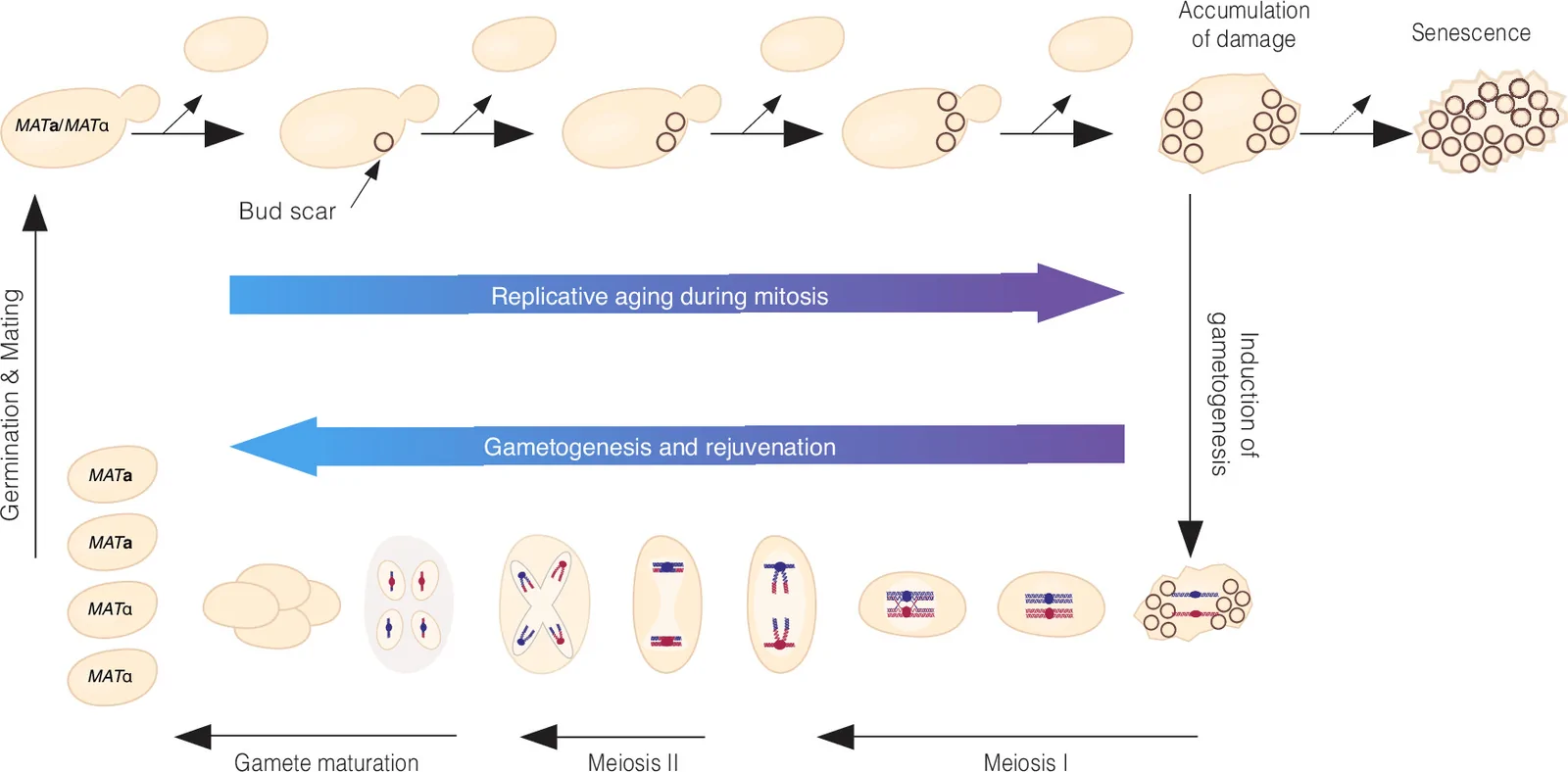
Replicative lifecycle and gametogenic rejuvenation in *Saccharomyces cerevisiae*. In vegetative growth, yeast cells divide asymmetrically, with the daughter cell born young while the mother cell accumulates aged and damaged components until reaching senescence. Replicative age of a single yeast cell is determined by the number of bud scars, which form at each division and are retained on the cell wall (top panel). Gametogenesis can be induced by nutrient depletion (downwards arrow, right). During gametogenesis, diploid yeast divide symmetrically, fully resetting their lifespan and leading to the formation of four equally young haploid gametes, even from aged precursor cells (bottom panel; note two different mating types created among gametes). Addition of nutrients induces germination, return to mitotic growth and formation of diploid cells upon mating (upward arrow, left).

During mitotic growth, *Saccharomyces cerevisiae* divides asymmetrically, producing young daughter cells and retaining age-associated damage in the aging mother cell^8–11^. This limits the number of daughter cells that a mother cell can produce (termed “replicative lifespan”) before reaching senescence. Excitingly, upon completion of gametogenesis, replicative lifespan of gametes is completely reset in *S. cerevisiae*, relative to that of the precursor cell^4,12,13^ (Fig. 1). The portability of this rejuvenation program was demonstrated by the observation that ectopic expression of Ndt80, a transcription factor necessary for completion of gametogenesis, extends replicative lifespan in mitotic cells^12,14^. Since this initial discovery, there has been interest in determining the gametogenic factors that drive lifespan resetting in gametes^4^. Drug-mediated inhibition of bulk autophagy prevents clearance of age-associated Hsp104 foci during gametogenesis as well as the completion of gametogenesis. This suggests that autophagy-mediated turnover of aged proteins and cellular components may play an important role in orchestrating gametogenic rejuvenation^12,15^.

Yeast gametogenesis, a highly experimentally tractable program that can be induced efficiently at will, presents an opportunity to gain mechanistic insights into the process of gametogenic rejuvenation. However, current techniques to quantify replicative lifespan of gametes significantly limit progress in the field. Quantification of gamete replicative lifespan has relied on scoring the progeny produced by a gamete over several days by continuous counting and manual removal of daughter cells via micromanipulation^12^. This technique is tedious, low throughput and labor-intensive. In fact, since gametogenesis-specific rejuvenation was initially observed (Ünal et al.^12^), only two other studies have used this technique to measure replicative lifespan of gametes^16,17^ and the specific pathways required for mediating gametogenic rejuvenation remain unknown. Although micromanipulation has historically been used to quantify replicative lifespan in vegetatively growing haploid yeast cells^18^, microfluidics-based approaches that increase throughput and lower manual labor have successfully been developed and utilized for yeast lifespan measurement in recent years. Multiple different trap designs have been used to capture and retain mother cells in imaging chambers with a constant media flow over prolonged periods of time. Parallelization of multiple imaging chambers allows acquisition of thousands of cells from multiple different genetic backgrounds over a few days in one single experiment^19–24^. While this method is rapidly replacing micromanipulation-based readouts for vegetatively growing haploid yeast cells, a microfluidics-based assay to investigate gamete replicative lifespan does not currently exist. The challenges associated with capturing and measuring gamete lifespan include: (i) purifying gametes from precursor cells and from the ascus that encloses four gametes together, (ii) preventing mating of neighboring haploid gametes, and (iii) the small size of gametes relative to precursor cells.

To overcome these limitations, we developed a microfluidic-based microscopy assay that allows high-throughput characterization of factors required for gametogenic rejuvenation in *S. cerevisiae*. We engineered inert genetic modifications and addressed the aforementioned challenges to enable microfluidic-based assessment of replicative lifespan in *S. cerevisiae* gametes derived from the SK1 background, which undergoes gametogenesis with high efficiency^25,26^. With this approach, we were able to isolate and purify single gametes from both young and aged precursor cells, which were then imaged over 72 h using a custom microfluidic device adapted from previously established device designs^21^, enabling quantitative analysis of gamete replicative lifespan.

Comparing the replicative lifespan of gametes derived from young versus aged precursor cells captured a broad spectrum of lifespan outcomes and confirmed the effects of known short- and long-lived mutants. Most excitingly, this system provided long-elusive molecular insight to gametogenic rejuvenation, showing that turnover of the endoplasmic reticulum (ER) mediated by selective autophagy (ER-phagy), but not mitophagy, is required during gametogenesis for complete lifespan resetting in gametes derived from aged precursor cells. We discovered that ER-phagy receptors Atg39 and Atg40 are required to drive full lifespan resetting through gametogenesis, providing a molecular foothold to dissect the impact of ER quality and inheritance in cellular deterioration. Moreover, this discovery exemplifies the potential of our microfluidic approach to uncover the mechanisms by which eukaryotic cells counteract age-associated damage.

## Results

### An approach for long-term marking of gametes to enable gamete lifespan analysis

**Fig. 2:**
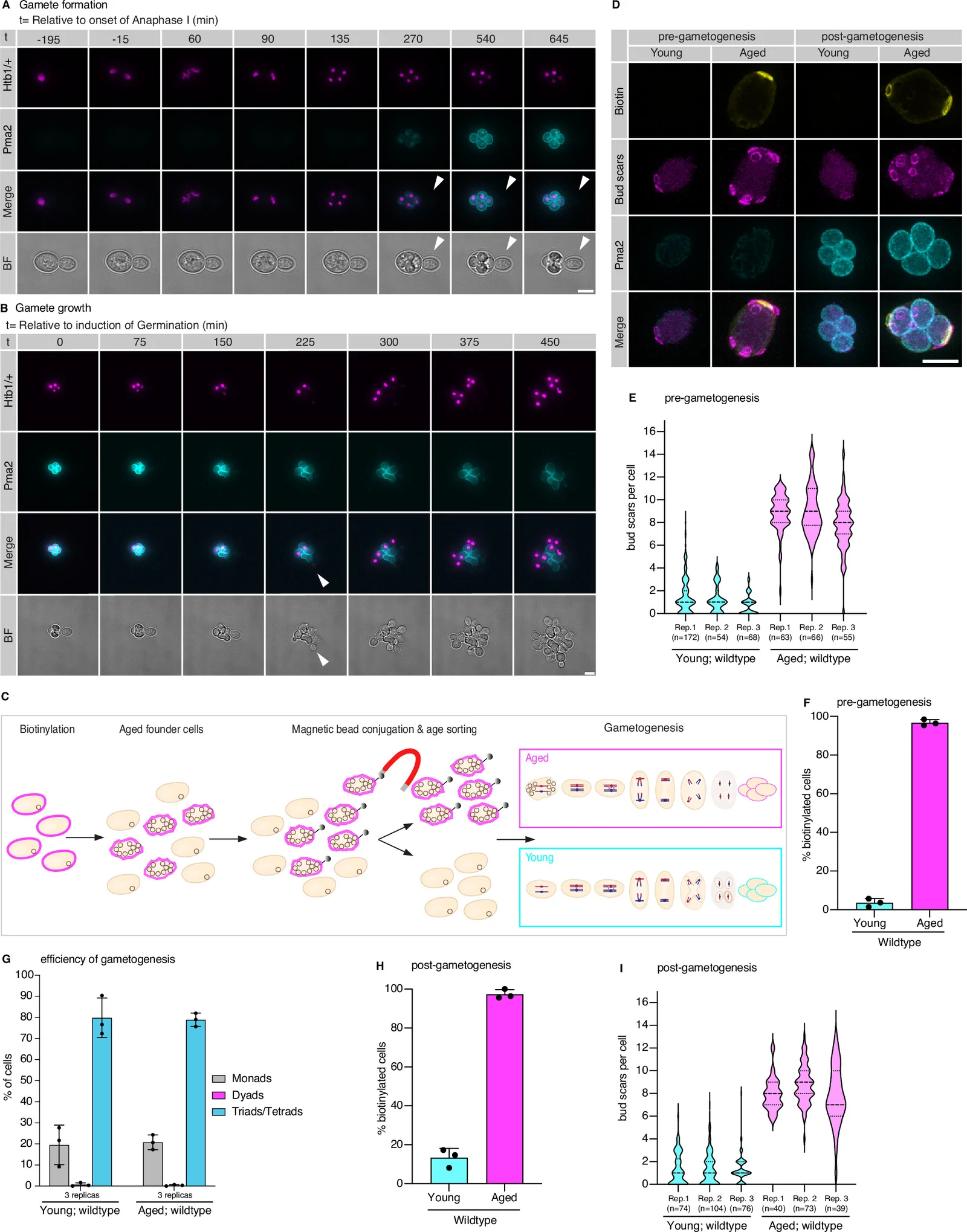
Gametogenesis-specific expression of Pma2 remains detectable post-germination in age-sorted gametes. **A** Montage of max-intensity projections of a cell progressing through gametogenesis visualizing Pma2-GFP (cyan), histone marker Htb1-mCherry (magenta) and brightfield (BF, gray). Arrows indicate a cell that failed to undergo gametogenesis. **B** Montage of germinating max-intensity projected tetrad visualizing Pma2-GFP (cyan), Htb1-mCherry (magenta) and brightfield (BF, gray), arrow indicates Pma2-GFP-negative emerging daughter cell. **C** Schematic depiction of biotin mediated age sorting, magenta indicates biotinylated cells. **D** Representative age sorted pre-gametogenic max-intensity projected cells and post-gametogenic tetrads visualizing biotin using streptavidin Alexa Fluor 546 conjugate (yellow), chitin rich bud scars visualized with Wheat Germ Agglutinin Alexa Fluor 350 conjugate (magenta) and Pma2-GFP (cyan). **E** Replicative age of young (1.16 ± 0.41, mean $\pm \,$SD, *n* = 3 replicas, *n* = 172; 54 and 68 respectively) and aged (8.55 ± 0.66, mean $\pm \,$SD, 3 replicas, *n* = 63, 66 and 55 respectively) wildtype cell populations. **F** Fraction of biotinylated cells in young (3.63 ± 2.12 %, mean $\pm \,$SD, 3 replicas, *n* = 175, 54 and 68 respectively) and aged (96.74 ± 1.58 %, mean $\pm \,$SD, 3 replicas, *n* = 65, 66 and 55 respectively) wildtype cell populations. **G** Fraction of tetrads in young (79.8 ± 9.43%; mean $\pm \,$SD, 3 replicas Rep.1&3 *n* = 300, Rep. 2 *n* = 281) and aged (aged: 78.89 ± 3.17 %, mean $\pm \,$SD, 3 replicas *n* = 300 each) post-gametogenic wildtype cell populations. **H** Fraction of biotinylated tetrads in young (13.38 ± 4.74%, mean $\pm \,$SD, *n* = 3 replicas, *n* = 74, 104 and 76 respectively) and aged (97.34 ± 2.33%, mean $\pm \,$SD, 3 replicas, *n* = 46, 73 and 39 respectively) wildtype populations after completion of gametogenesis. **I** Replicative age of young (1.31 ± 0.04, mean $\pm \,$SD, 3 replicas, *n* = 74, 104 and 76 respectively) and aged (8.17 ± 0.54, mean $\pm \,$SD, 3 replicas, *n* = 40, 73 and 39 respectively) wildtype tetrad populations. Bar graphs show mean $\pm \,$SD, confidence intervals were calculated with Wilson score method. Violin plots indicate median (solid lines), 25th and 75th percentiles (dotted lines). Scale bars, 5 µm. Source data are provided as a Source Data file.

To microscopically distinguish gametes from precursor cells, we looked for markers exclusively expressed in gametes. One such gene, *PMA2*, encodes a plasma membrane proton pump^27–29^. Its paralogous protein, Pma1, is highly expressed during vegetative growth, with a long half-life and asymmetric retention in mother cells (Henderson et al.^30^). *PMA1* is essential for viability but *PMA2* is not, and Pma2 protein is barely detectable during mitotic growth^29–32^. *PMA2* translation and protein abundance are upregulated late in gametogenesis (Supplementary Fig. 1A–C)^33^. We created a transgenic reporter strain carrying a Pma2-GFP fusion protein under control of its endogenous promoter and tracked its expression timing relative to meiotic divisions by using a fluorescently labeled chromatin marker (Htb1-mCherry). Pma2-GFP expression was upregulated 1–2 h following anaphase II and predominantly localized to the gamete plasma membranes (Fig. 2A). Importantly, Pma2-GFP was not detected in cells that fail to undergo gametogenesis (arrows, Fig. 2A). Next, we analyzed Pma2-GFP expression in gametes undergoing germination, their first mitotic division. We found that gamete-derived daughter cells lacked Pma2-GFP signal, which was fully retained on the plasma membrane of gametes for several rounds of cell division (Fig. 2B). This expression pattern analysis revealed that Pma2-GFP is a reliable, specific, and retained marker of gametes, exhibiting gamete-specific retention beyond germination, making it an optimal reporter to distinguish gametes from cells that fail to complete gametogenesis and vegetative daughter cells.

### Cell populations efficiently sorted by age progress robustly through gametogenesis

To quantify gametogenic rejuvenation, we first separated pre-meiotic diploid cell populations by replicative age using an established protocol^34^. Briefly, a founder cell population was biotin-labeled with sulfo-NHS-LC-biotin, which specifically and permanently binds to cell surface proteins that are retained on the founder (mother) cells during subsequent mitotic divisions. These biotin-labeled founder cells were then replicatively aged and incubated with anti-biotin magnetic beads that enabled their isolation from their young daughter cells. The labeled cell suspension was sequentially passed through two magnetic columns to increase purity, followed by elution. Flow-through cells from initial magnetic sorting provide a pure population of young cells. The eluate from the second column represents the aged cell population (Fig. 2C, see “Methods”). Sorted cell populations were stained with fluorescent dyes visualizing both biotin and the chitin-rich, ring-like structures called bud scars on the yeast cell wall, each of which represents a budding event and thus reveals the replicative age of a cell^35^. We measured the purity and age of the sorted population by fluorescent microscopy of these markers (Fig. 2D, left panel). With our consecutive-sort protocol, we consistently obtained near pure young (3.63% biotinylated cells with a mean replicative age of 1.16), and aged (96.74% biotinylated cells with a mean replicative age of 8.55) cell populations (Fig. 2E, F). Next, we induced gametogenesis in the separated cell populations and analyzed efficiency of gametogenesis after 24 h. We found that 79.8% of young cells and 78.89% of aged cells successfully formed visibly distinct tetrads (Fig. 2G) and that gametes from young and aged cell population both robustly expressed Pma2-GFP after completion of gametogenesis (Fig. 2D, right panel). Furthermore, we re-evaluated the purity and the age of the sorted population following gametogenesis, confirming that the relative distributions of young and aged populations were maintained (Fig. 2H, I). In summary, we reproducibly isolated young and aged cell populations with high purity and observed no negative effect of our experimental regime on the efficiency of gametogenesis.

### Isolation of single gametes for microfluidics-based replicative lifespan profiling

To capture replicative lifespan of yeast gametes with microfluidic devices^21^, we had to overcome four major obstacles. First, without inert mutations to disrupt associations between individual cells, they were not suitable for microfluidics-based applications. Second, gametogenesis produces gametes with two mating types, thus we had to prevent initiation of mating in response to mating pheromones, which leads to growth arrest and abnormal cell morphology. Third, gametes are packaged into tetrad structures inside an enclosed ascus and do not separate during germination (Fig. 2B), rendering assessment of replicative lifespan impossible. Thus, we had to find a way to separate tetrads into single gametes before loading into the microfluidics imaging chambers. Fourth, separated gametes are significantly smaller than vegetative growing cells, and do not fit into the custom-built U-traps in our microfluidics devices that are built to capture vegetatively growing haploid cells (Supplementary Fig. 2A–D adapted from ref. ^21^).

**Fig. 3:**
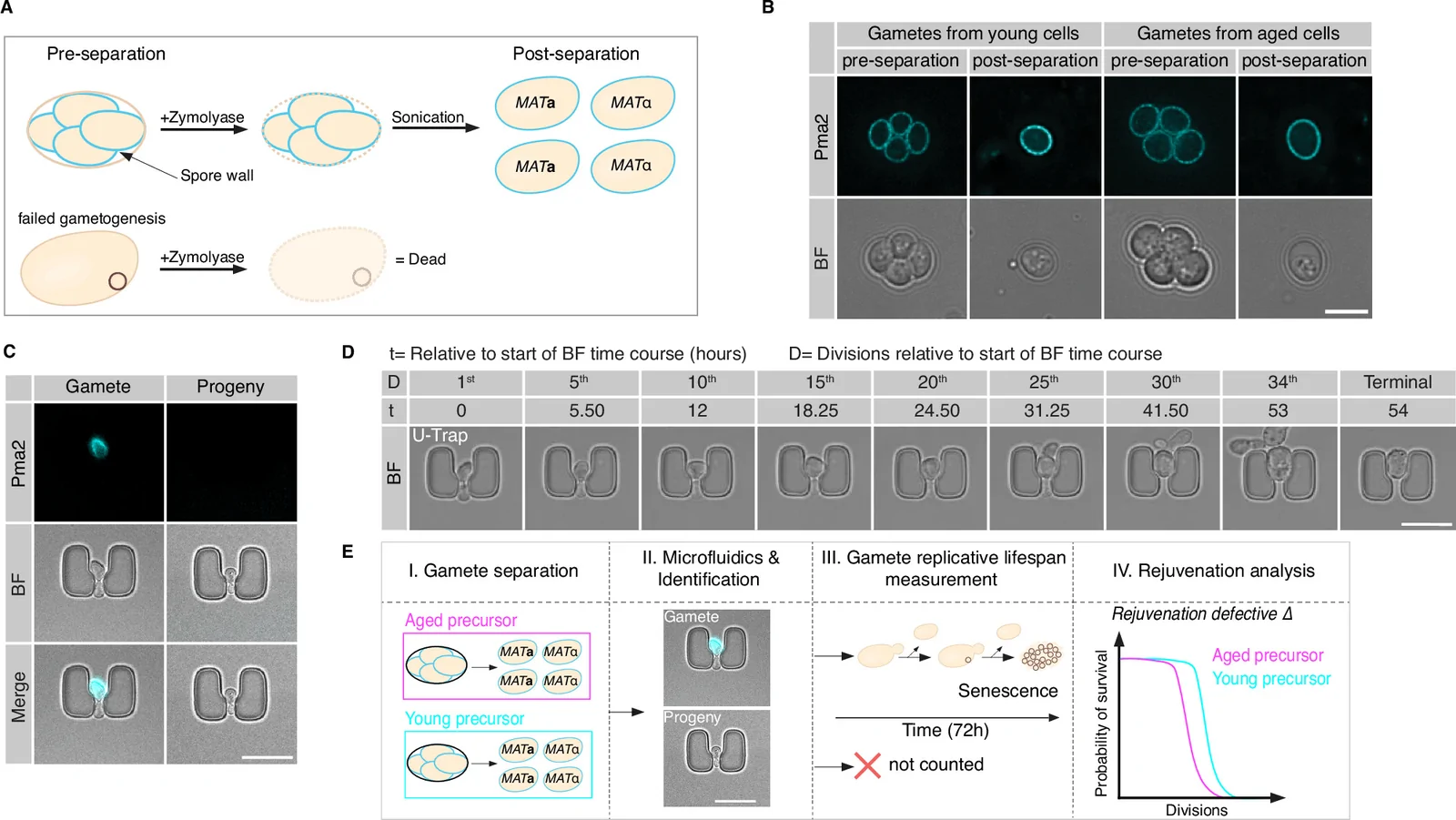
Separation of tetrads into single sterile gametes to assess gamete replicative lifespan with microfluidics. **A** Schematic depiction of tetrad separation procedure. **B** Example single z-slices of pre- and post-separated young (left panel) and aged (right panel) tetrads and gametes, respectively, visualizing brightfield (BF, gray) and Pma2-GFP (cyan). **C** Example image of Pma2-GFP positive gamete (left panel) and Pma2-GFP negative gamete progeny (right panel) trapped in microfluidics-based devices visualizing brightfield (BF, gray) and Pma2-GFP (cyan). **D** Representative gamete trapped in microfluidics imaging unit visualizing every 5th division until the terminal 34th division over the time of 54 h visualized in brightfield (BF, gray). **E** Schematic depiction of the developed pipeline to assess gametes replicative lifespan. Scale bars are 5 µm.

Cells from the SK1 background are known to form both covalent (mother-daughter cell adherence) and non-covalent (flocculation) interactions, which complicate microfluidics-based applications due to elevated clogging. To mitigate this, we introduced an inert point mutation into *AMN1* to promote efficient mother-daughter cell separation^36^. We also deleted *FLO8*, a transcription factor required for flocculation^37^ to prevent the cells from clumping together. During gametogenesis, a diploid yeast cell undergoes two meiotic divisions forming a tightly packed tetrad containing four haploid gametes with a 2:2 segregation of the two mating types *MAT***a** and *MAT*α (Figs. 1, 3A). To avoid triggering the mating response in gametes, we replaced the endogenous promoter of the mating response signaling scaffold gene *STE5*^38^, with an anhydrotetracycline-inducible promoter (Supplementary Fig. 2E)^39^. We confirmed sterility and inducible fertility by mixing *MAT***a** and *MAT*α cells on control and anhydrotetracycline-containing plates and scoring formation of mated zygotes (Supplementary Fig. 2F).

To obtain single gametes and to eliminate cells that did not proceed through gametogenesis, we incubated post-gametogenesis cultures with zymolyase. This enzyme hydrolyzes the main structural polysaccharide in the cell wall, thus lysing non-gametes and weakening the ascus protecting the four gametes within a tetrad (Fig. 3A). Zymolyase-treated tetrads were then gently sonicated to disassociate them into single gametes, and presence of the Pma2-GFP marker in single gametes was confirmed by fluorescence microscopy (Fig. 3A, B).

When loading separated gametes directly into our custom microfluidics devices (Supplementary Fig. 2A–D adapted from devices established in ref. ^21^), gametes were not retained in the traps due to their small size. To solve this issue, gametes were first grown in rich media following their separation for 3 h, which was sufficient to increase their size but not long enough to allow the first cell division to occur in the vast majority of gametes (Supplementary Fig. 2G, H). These pre-grown gametes were then loaded into the microfluidic devices and an image of the whole region of interest was taken in the FITC and brightfield channels. By assessing Pma2-GFP expression, we could reliably determine if a trapped cell was a gamete, or a progeny cell generated during re-growth (Fig. 3C). Next to quantify replicative lifespan of gametes, brightfield images were acquired every 15 min over 72 h, capturing every cell division (Fig. 3D and Supplementary Fig. 2I). Together, these steps constitute a high-throughput microfluidics-based pipeline that enables assessment of gamete replicative lifespan, and identification of mutants that specifically impact the rejuvenation of aged cells (Fig. 3E).

### Wild-type gametes from aged precursor cells display complete resetting of replicative lifespan

**Fig. 4:**
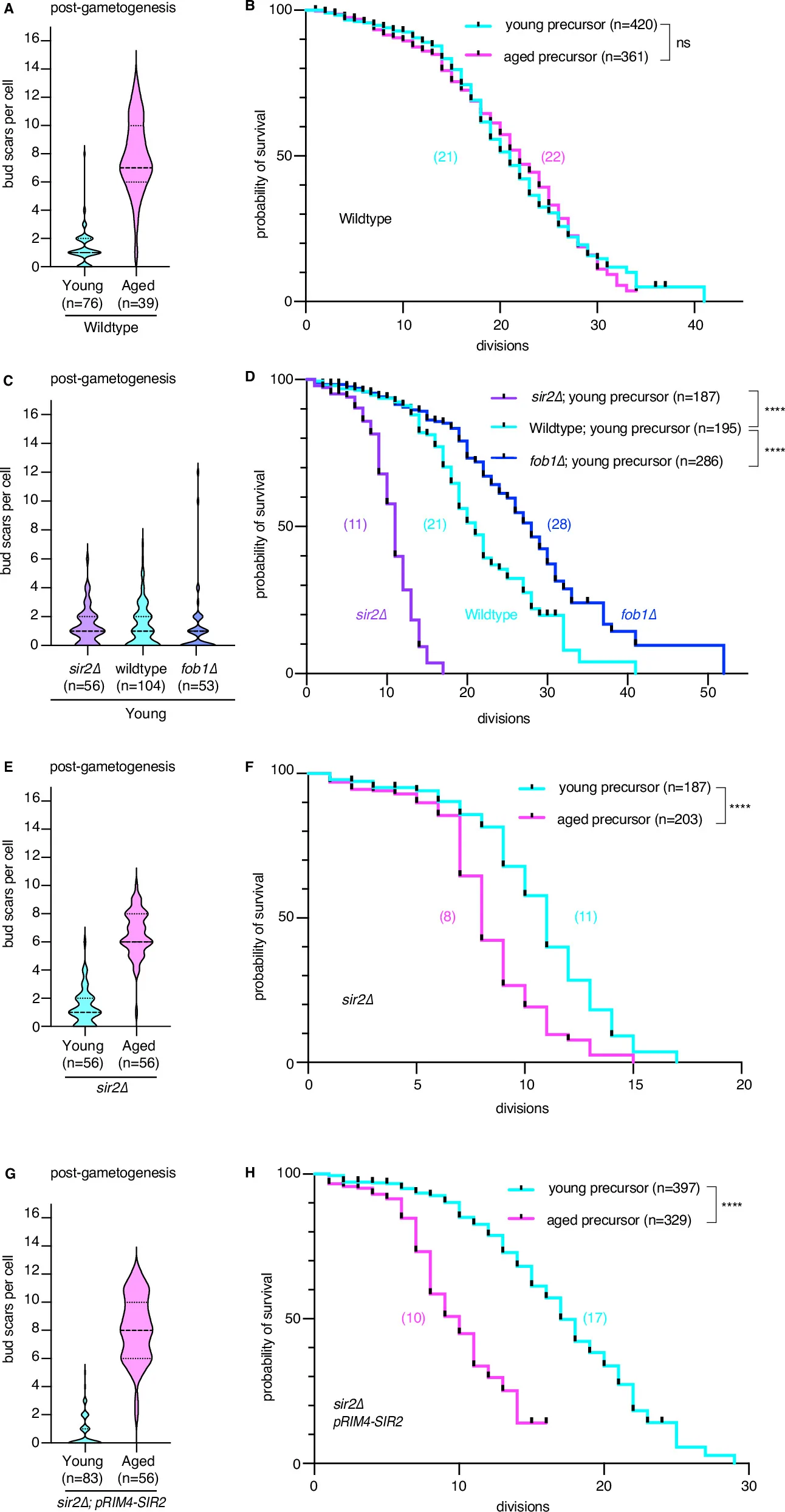
Replicative lifespan resets in wildtype gametes but rejuvenation fails in *sir2∆* mutants through a gametogenesis-independent mechanism. **A** Replicative age of young (1.3 ± 1.2, mean ± SD, *n* = 76) and aged (7.8 ± 2.6, mean $\pm \,$SD, *n* = 39) wildtype gamete populations, same data as shown in Fig. 2I for Rep. 3. **B** Replicative lifespan of young (median lifespan = 21, *n* = 420, 223 censored subjects) and aged (median lifespan = 22, *n* = 361, 213 censored subjects, *p* = 0.982 compared to young) wildtype gametes. **C** Replicative age of young *sir2*∆ (1.3 ± 1.3, mean $\pm \,$SD, *n* = 56), wildtype (1.4± 1.5, mean $\pm \,$SD, *n* = 104) and *fob1*∆ (1.2± 2.2, mean $\pm \,$SD, *n* = 53) gamete populations. **D** Replicative lifespan of young *sir2*∆ (median lifespan = 11, *n* = 187, 80 censored subjects, *p* = 1.31 × 10^−40^ compared to wildtype), wildtype (median lifespan = 21, *n* = 195, 103 censored subjects) and *fob1*∆ (median lifespan = 28, *n* = 286, 174 censored subjects, *p* = 6 × 10^−6^ compared to wildtype) gametes. **E** Replicative age of young (1.3 ± 1.3, mean $\pm \,$SD, *n* = 56, same data as shown in Fig. 4C) and aged (6.6 ± 1.6, mean $\pm \,$SD, *n* = 56) *sir2*∆ gamete populations. **F** Replicative lifespan of young (median lifespan = 11, *n* = 187, 80 censored subjects, same data as shown in Fig. 4D) and aged *sir2*∆ (median lifespan = 8, *n* = 203, 103 censored subjects, *p* = 7.388 × 10^−11^ compared to young) gametes. **G** Replicative age of young (0.72 ± 1.1, mean $\pm \,$SD, *n* = 83) and aged (8.1 ± 2.3 mean $\pm \,$SD, *n* = 56) *pRIM4-SIR2 sir2*∆ gamete populations. **H** Replicative lifespan of young (median lifespan = 17, *n* = 397, 266 censored subjects) and aged *pRIM4-SIR2 sir2*∆ (median lifespan = 10, *n* = 329, 219 censored subjects, *p* = 2.763 × 10^−26^ compared to young) gametes. Violin plots indicate median (solid lines), 25th and 75th percentiles (dotted lines). Confidence intervals were calculated with Wilson score method and statistical significance for differences between replicative lifespan differences was calculated using the Log-rank (Mantel-Cox) test, tick marks on survival curves represent censored events. *P* values are indicated (ns, *P* > 0.05, *P ≤ 0.05, **P ≤ 0.01, ***P ≤ 0.001, ****P ≤ 0.0001). Source data are provided as a Source Data file.

It has previously been shown that replicative lifespan is completely reset after gametogenesis^12^. This study used a micromanipulator to continuously count and remove daughter cells of gametes previously placed onto agar plates, and was assessed only in the W303 and A364a strain backgrounds. Thus, we tested whether gametes also showed a complete resetting of their replicative lifespan using the approach described above in the SK1 background. Toward this end, we sorted diploid wild-type cells following our protocol established here (Fig. 2C) and analyzed the replicative age of young and aged pre-gametogenic cell populations (Supplementary Fig. 3A, B). Separated cell populations were transferred to gametogenesis-inducing media; after 24 h both populations successfully completed gametogenesis (Supplementary Fig. 3C), resulting in pure tetrad populations from replicatively aged and young precursor cells (Fig. 4A and Supplementary Fig. 3D). We acquired and quantified the replicative lifespan of gametes using our pipeline and confirmed that the median replicative lifespan of gametes from both young and aged precursors cells was the same and reproducible (Fig. 4B, *p* = 0.982; independent biological replicate in Supplementary Fig. 3E–J). These results confirm that the high-throughput microfluidic-based pipeline we developed can capture and quantify gametogenic rejuvenation.

### Microfluidics-based analyses of gamete lifespans capture a wide range of outcomes among known short- and long-lived mutants

To test the sensitivity of our system in detecting differences in gamete replicative lifespan, we quantified lifespans of young gametes derived from established short and long-lived mutants. Deletion of the gene encoding Sir2, a NAD^+^- dependent histone deacetylase, is known to decrease replicative lifespan of yeast by approximately 50%, in part due to loss of rDNA locus silencing, leading to the generation and accumulation of extrachromosomal rDNA circles^40,41^. In contrast, deletion of the gene encoding Fob1, an rDNA spacer replication fork barrier binding protein, is reported to extend lifespan in yeast by up to approximately 40%, due to decreased rDNA recombination and thus fewer extrachromosomal rDNA circles^42^.

We sorted *sir2∆*, wild-type, and *fob1∆* diploid cells (Fig. 2C), and verified the sorting efficiency and replicative age of the young pre-gametogenic cell populations (Supplementary Fig. 3K, L). We induced gametogenesis in the separated cell populations and found after 24 h, 87.3% of *sir2∆* cells, 90.4% of wildtype cells and 73.7% of *fob1∆* cells successfully completed gametogenesis (Supplementary Fig. 3M). Upon confirming purity of sorting and replicative age in tetrads (Fig. 4C and Supplementary Fig. 3N), we separated them into single gametes and acquired their replicative lifespan over 72 h. The median replicative lifespan for gametes derived from young precursor *sir2∆* cells was 11 divisions, significantly lower than gametes derived from young wild-type cells, with a median lifespan of 21 (Fig. 4D, *p* = 1.31 × 10^−40^). Gametes derived from young *fob1∆* precursor cells, on the other hand, had a significantly increased median lifespan of 28 compared to wildtype (Fig. 4D, *p* = 6 × 10^−6^). Thus, our microfluidic platform and workflow can capture a range of gamete lifespans from young precursor cells with mutations in established longevity-linked genes resulting in lifespan measurements that closely reflect published replicative lifespan of haploid cells acquired using microdissections (Supplementary Table 1).

### Gametes lacking *SIR2* fail to reset replicative lifespan through a mechanism that is independent of gametogenesis

While testing our pipeline, we observed that gametes derived from aged *sir2∆* precursor cells did not display complete resetting of replicative lifespan. We sorted *sir2∆* pre-gametogenic diploid cells into a young cell population (same data as Supplementary Fig. 3K, L) and an aged cell population (Supplementary Fig. 4A, B). 87.3% of the young population (same data as Supplementary Fig. 3M) and 68.0% of old the population completed gametogenesis after 24 h (Supplementary Fig. 4C). We verified purity and replicative age of post-gametogenic cell populations before measuring their lifespan (Fig. 4E and Supplementary Fig. 4D, same data as shown in Fig. 4C and Supplementary Fig. 3N for young). Strikingly, the replicative lifespan of gametes derived from aged *sir2∆* precursor cells revealed a median replicative lifespan of 8 divisions, significantly lower than for gametes derived from young *sir2∆* precursor cells, which had a median lifespan of 11 (Fig. 4F, *p* = 7.388 × 10^−11^ same data as shown in Fig. 4D for young).

To test whether ectopic expression of *SIR2* during gametogenesis can rescue the lack of replicative lifespan resetting observed in *sir2∆* gametes, we expressed the *SIR2* ORF under the control of a *RIM4* promoter fragment in *sir2∆* cells. Although expression levels are not perfectly matched, *RIM4* expression closely resembles *SIR2* expression pattern in gametogenesis and the *pRIM4-SIR2* rescue construct resulted in robust gametogenesis-specific expression of Sir2 (Supplementary Fig. 4E–I)^43,44^. We sorted pre-gametogenic diploid *sir2∆* cells carrying this rescue construct into a young and aged cell population (Supplementary Fig. 4J–K) and verified purity and replicative age of sorted cell populations upon gametogenesis before analyzing replicative lifespan of gametes (Fig. 4G and Supplementary Fig. 4L, M). Gametes derived from aged *pRIM4-SIR2 sir2∆* precursor cells showed a median replicative lifespan of 10, which was still significantly lower than gametes derived from young *pRIM4-SIR2 sir2∆* cells, with a median lifespan of 17 (Fig. 4H). We note a slightly increased replicative lifespan of gametes derived from both young and aged *pRIM4-SIR2 sir2Δ* precursor cells compared to those derived from *sir2∆* cells, which may be due to residual Sir2 protein observed in late gametes that could carry over through the first cell divisions (Supplementary Fig. 4I, right most band). In summary, *sir2∆* gametes do not completely reset their lifespan following gametogenesis; however, this phenotype cannot be rescued by providing Sir2 during gametogenesis, suggesting that aged *sir2∆* cells might accumulate defects that cannot be rescued by the gametogenic rejuvenation program.

### Selective Autophagy is necessary to reset replicative lifespan during gametogenesis

**Fig. 5:**
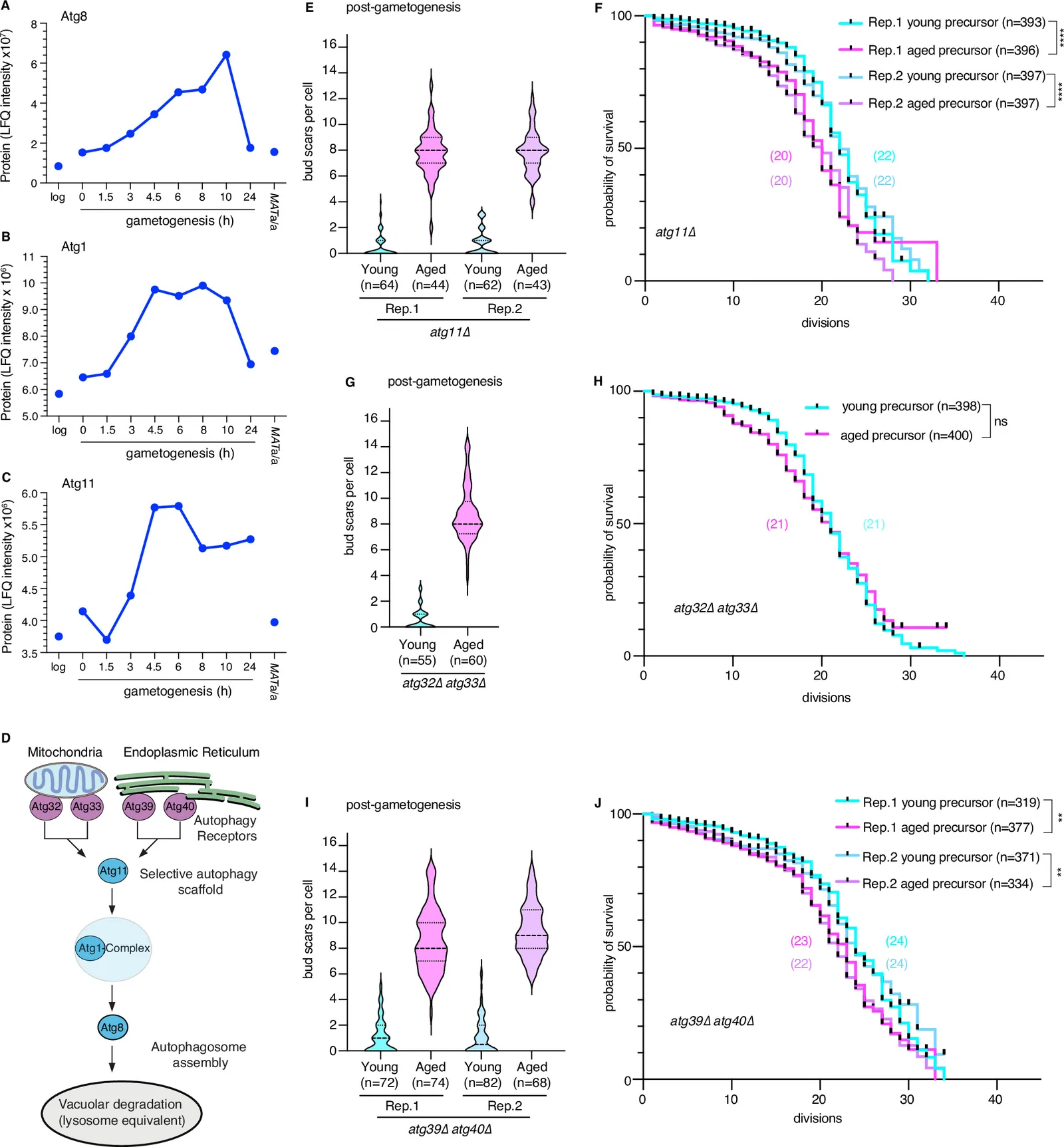
Selective autophagy of the ER but not the mitochondria is necessary to reset replicative lifespan during gametogenesis. **A**–**C** Protein levels (mass spectrometry) of autophagy factors; vegetative growth (log), gametogenesis (h), and gametogenesis incompetent diploids (*MAT***a**/**a**), previously published dataset^44^. **A** Atg8. **B** Atg1. **C** Atg11. **D** Schematic depiction of Atg11-mediated selective autophagy. Atg11 binds selective autophagy receptors on targets, followed by autophagosome assembly and delivery to the vacuole for degradation. **E** Replicative age of *atg11*∆ tetrad populations: Rep. 1 young (0.53 ± 0.91, mean $\pm \,$SD, *n* = 64), aged (7.8 ± 1.8, mean $\pm \,$SD, *n* = 44). Rep. 2 young (0.82 ± 1, mean $\pm \,$SD, *n* = 62), aged (7.9 ± 1.7, mean $\pm \,$SD, *n* = 43). **F** Replicative lifespan of *atg11*∆ gametes: Rep.1 young (median lifespan = 22, *n* = 393, 267 censored subjects); aged (median lifespan = 20, *n* = 396, 255 censored subjects, *p* = 2 × 10^−6^). Rep. 2 young (median lifespan = 22, *n* = 397, 280 censored subjects); aged (median lifespan = 20, *n* = 397, 259 censored subjects, *p* = 3 × 10^−6^ compared to young). **G** Replicative age of young (0.6 ± 0.85, mean $\pm \,$SD, *n* = 55), and aged (8.8 ± 2.1, mean $\pm \,$SD, *n* = 60) *atg32∆ atg33*∆ gamete populations. **H** Replicative lifespan of young (median lifespan = 21, *n* = 398, 154 censored subjects) and aged *atg32∆ atg33*∆ double mutants (median lifespan = 21, *n* = 400, 192 censored subjects, *p* = 0.7414 compared to young) gametes. **I** Replicative age of *atg39∆ atg40*∆ tetrad populations: Rep. 1 young (1.1 ± 1.4, mean $\pm \,$SD, *n* = 72) and aged (8.6 ± 2.5, mean $\pm \,$SD, *n* = 74); Rep. 2 young (1.1 ± 1.5, mean $\pm \,$SD, *n* = 82) and aged (9.4 ± 2, mean $\pm \,$SD, *n* = 68). **J** Replicative lifespan of *atg39∆ atg40*∆ gametes: Rep.1 young (median lifespan = 24, *n* = 319, 195 censored subjects); aged (median lifespan = 23, *n* = 377, 238 censored subjects, *p* = 0.0030 compared to young). Rep. 2 young (median lifespan = 24, *n* = 371, 240 censored subjects); aged (median lifespan = 22, *n* = 334, 219 censored subjects, *p* = 0.0080 compared to young). Violin plots indicate median (solid lines), 25th and 75th percentiles (dotted lines). Statistical significance for replicative lifespan differences was calculated using the Log-rank (Mantel-Cox) test, tick marks on survival curves represent censored events. *P* values are indicated (ns, *P* > 0.05, *P ≤ 0.05, **P ≤ 0.01, ***P ≤ 0.001, ****P ≤ 0.0001) Source data are provided as a Source Data file.

After validating our technique, we sought to determine whether quality control mechanisms known to be active during gametogenesis also promote rejuvenation. Autophagy mediates degradation via the vacuole (lysosome equivalent in yeast) of damaged organelles and proteins^45^. Autophagy pathways decline during aging, leading to loss of proteostasis and accumulation of cellular damage. Artificially increasing autophagy factors can lead to lifespan extension^15,46–48^. Both Atg1, an essential kinase required for initiation of autophagosome formation, and Atg8, which is required for formation of the autophagosome membrane, are upregulated during gametogenesis (Fig. 5A, B; Supplementary Fig. 5A–D)^43,49,50^. In yeast, the general autophagy machinery is essential for meiotic entry as well as progression, which hinders evaluating the effect of bulk autophagy on gamete rejuvenation^51–53^. However, Atg11, the main selective autophagy scaffold protein required for Atg1 recruitment to selective autophagy receptors on specific cargos, is also dynamically upregulated during gametogenesis (Fig. 5C, D; Supplementary Fig. 5E, F) and we found that cells deleted for Atg11 can undergo gametogenesis^43,54,55^. Therefore, we tested whether cellular quality control via selective autophagy plays a direct role in gametogenic rejuvenation by deleting *ATG11* and measuring the rejuvenation potential with our established pipeline.

We sorted pre-gametogenic cells into young and aged cell populations and 24 h after induction verified the efficiency of gametogenesis (Supplementary Fig. 5G–I). Replicative age of tetrad population was confirmed by quantifying bud scars per tetrad and number of biotinylated tetrads in the population (Fig. 5E and Supplementary Fig. 5J). Strikingly, when quantifying the replicative lifespan of gametes, we found a significant difference between gametes from aged and young *atg11*∆ precursor cells that was highly reproducible (Fig. 5F, Rep.1, *p* = 2 × 10^−6^, Rep.2, *p* = 3 × 10^−6^ compared to Fig. 4B). This result suggests that Atg11-mediated selective autophagy is essential for full rejuvenation of gametes derived from aged precursor cells.

### Mitophagy is not necessary to reset replicative lifespan during gametogenesis

Atg11 recruits the autophagy machinery upon binding to specific selective autophagy receptors that can mark select organelles for degradation (Fig. 5D), thus we wondered if selective autophagy of a specific compartment is necessary for gametogenic rejuvenation. To address this, we first investigated a potential role for Atg32, an autophagy receptor localized on the outer mitochondria membrane that mediates selective removal of damaged and excess mitochondria via mitophagy^56–58^. Atg32 is strongly upregulated in mid-meiosis (Supplementary Fig. 6A–C)^43^ and specific mitochondrial remodeling and inheritance during gametogenesis has been described^59,60^.

We deleted *ATG32* homozygously in diploid cells and tested their gametogenic rejuvenation potential. We again sorted pre-gametogenic cells into young and aged cell populations, determined efficiency of gametogenesis and analyzed biotin label and replicative age of post gametogenic cell populations (Supplementary Fig. 6D–H). No significant difference in replicative lifespan was observed between gametes from aged compared to young *atg32*∆ precursor populations (Supplementary Fig. 6I, *p* = 0.2691). Atg33, which is localized on the outer mitochondria membrane also confers selectivity during mitophagy under certain growth conditions and is upregulated during meiosis (Supplementary Fig. 6J–L)^61^. To exclude the possibility that *ATG33-*mediated mitophagy acts in parallel thereby masking a possible rejuvenation phenotype of *atg32*∆ mutants, we constructed *atg32∆ atg33*∆ double mutants and tested their rejuvenation potential. We age-sorted *atg32∆ atg33*∆ pre-gametogenic cells into young and aged cell populations, verified efficiency of gametogenesis and analyzed biotin labeling and replicative age of post gametogenic cell populations (Fig. 5G and Supplementary Fig. 6M–P). As for *atg32*∆ single mutants, no significant difference in replicative lifespan was observed in *atg32∆ atg33*∆ gametes (Fig. 5H, *p* = 0.7414). These experiments suggest that selective mitophagy is dispensable for gametogenic rejuvenation. Failure of *atg32∆ atg33*∆ to phenocopy the rejuvenation defect in *atg11*∆ cells implies that a different specific arm of selective autophagy mediated by Atg11 is necessary for gametogenic rejuvenation.

### ER-phagy is necessary for full gametogenic lifespan resetting

It has been shown that Atg11-dependent selective autophagy mediated by the autophagy receptors Atg40, and to a lesser extent Atg39, control ER inheritance during gametogenesis^62,63^. Atg39 and Atg40 are upregulated during gametogenesis (Supplementary Fig. 7A–F)^43^ and meiotic remodeling of the ER combined with ER-phagy prevent specific subpopulations of the ER from being passed onto gametes. It has been proposed that these pathways constitute a quality control mechanism to ensure gametogenic rejuvenation, however this has not been directly tested^62,63^.

To determine if Atg39, a transmembrane autophagy receptor in the perinuclear ER mediating autophagy, impacts gametogenic rejuvenation, we first sorted diploid *atg39*∆ cells into a young and aged cell population (Supplementary Fig. 7G, H)^64^. In the young and aged populations, 70.0% and 82.0% completed gametogenesis, respectively (Supplementary Fig. 7I). We verified age separation of post-gametogenic populations before measuring lifespan of gametes with our microfluidic devices (Supplementary Fig. 7J, K). We found no significant difference for replicative lifespan of *atg39*∆ gametes derived from aged or young precursors (Supplementary Fig. 7L, *p* = 0.5881).

Next, we checked if gametogenic reset of replicative lifespan is impaired in cells lacking Atg40, an autophagy receptor localized at the cortical and cytoplasmic ER mediating fragmentation and encapsulation of ER subdomains^64^. Pre-gametogenic *atg40*∆ mutant cells were sorted into young and aged cell populations, efficiency of gametogenesis as well as biotin label and replicative age of post-gametogenic cell populations was determined upon gametogenesis (Supplementary Fig. 7M–Q). Similar to *atg39*∆ cells, no significant difference in replicative lifespan was observed between *atg40*∆ gametes derived from aged or young precursors (Supplementary Fig. 7R, *p* = 0.3077).

Because there is known functional redundancy between Atg39 and Atg40^62,64^, we tested if *atg39∆ atg40*∆ cells were still capable of completely resetting replicative lifespan during gametogenesis. We verified purity and replicative age upon sorting *atg39∆ atg40*∆ diploid cells into young and aged populations (Supplementary Fig. 8A, B, Rep.1). Upon gametogenesis, we determined efficiency of gametogenesis and assured sorting purity as well as replicative age (Fig. 5I**;** Supplementary Fig. 8C, D, Rep.1). Excitingly, lifespan analysis of gametes from aged *atg39∆ atg40*∆ precursor cells revealed a significantly reduced median replicative lifespan compared to gametes from young precursor cells (Fig. 5J, Rep.1, *p* = 0.0030). The failure to fully rejuvenate in aged *atg39∆ atg40*∆ gametes was highly reproducible (Fig. 5I, J, Rep.2, *p* = 0.0080; Supplementary Fig. 8A–D). Together, these data revealed that Atg39 and Atg40 both contribute through partially redundant mechanisms to gametogenic rejuvenation, and that full replicative lifespan resetting during gametogenesis depends on the presence of these selective ER-phagy receptors.

### Meiosis specific expression of *ATG40* is sufficient to rescue resetting of replicative lifespan in aged ER-phagy-deficient cells

To determine whether this difference in replicative lifespan between gametes from aged and young *atg39∆ atg40*∆ precursor cells is due to lack of selective ER-phagy specifically during gametogenesis, we tested if ectopic meiotic expression of *ATG40* is sufficient to rescue it. We constructed a *atg39∆ atg40*∆ double mutant with the *ATG40* ORF under the control of a *NDT80* promoter fragment, which displays meiosis-specific expression with a pattern that closely resembles *ATG40* expression (Supplementary Fig. 9A and Supplementary Fig. 7D) as well as a *atg39∆ atg40*∆ double mutant control strain^14^. We confirmed gametogenesis-specific expression of Atg40 by immunoblotting (Supplementary Fig. 9B) and performed rejuvenation assays in independent duplicates.

**Fig. 6:**
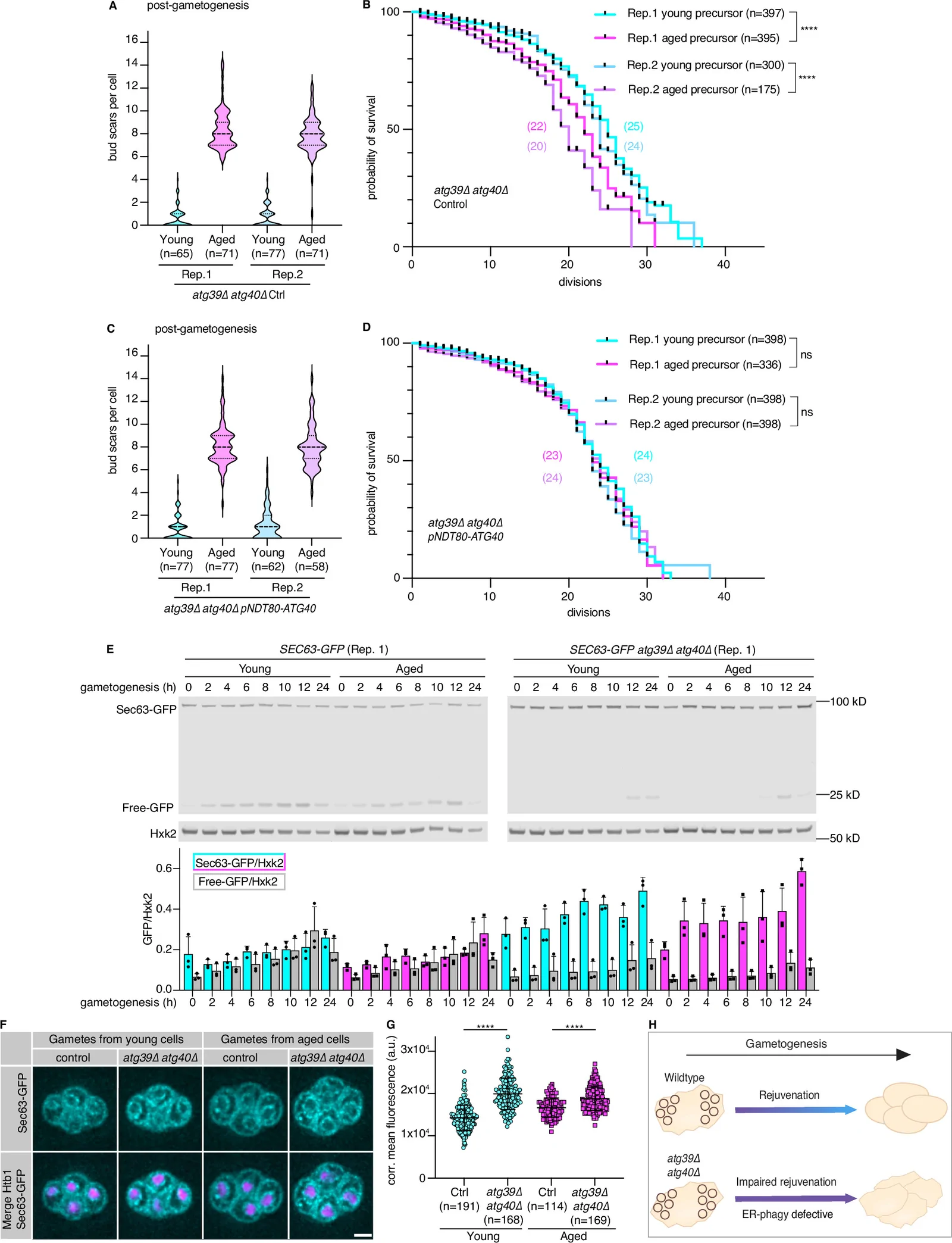
Expression of *ATG40* during gametogenesis rescues replicative lifespan resetting in cells lackcking ER-phagy receptors*.* **A** *atg39∆ atg40*∆ tetrads, replicative age: Rep. 1 young (0.62 ± 0.92, mean $\pm \,$SD, *n* = 65); aged (8.4 ± 1.7, mean $\pm \,$SD, *n* = 71). Rep. 2 young (0.74 ± 0.97, mean $\pm \,$SD, *n* = 77); aged (7.8 ± 1.6, mean $\pm \,$SD, *n* = 71). **B** *atg39∆ atg40*∆ gametes, replicative lifespan: Rep.1 young (median lifespan = 25, *n* = 397, 229 censored), aged (median lifespan = 22, *n* = 395, 277 censored, *p* = 8.6 × 10^−5^ compared to young). Rep. 2 young (median lifespan = 24, *n* = 300, 185 censored, 1 technical replicate), aged (median lifespan = 20, *n* = 175, 122 censored, 1 technical replicate, *p* = 4 × 10^−6^ compared to young). **C** *atg39∆ atg40∆ pNDT80-ATG40* tetrads, replicative age: Rep. 1 young (0.87 ± 1.1, mean $\pm \,$SD, *n* = 77), aged (8.1 ± 1.8, mean $\pm \,$SD, *n* = 77). Rep. 2 young (1.2 ± 1.4, mean $\pm \,$SD, *n* = 62), aged (8.0 ± 1.9, mean $\pm \,$SD, *n* = 58). **D** *atg39∆ atg40∆ pNDT80-ATG40* gametes replicative lifespan: Rep.1 young (median lifespan = 24, *n* = 398, 231 censored), aged (median lifespan = 23, *n* = 336, 242 censored, one technical replica, *p* = 0.4519 compared to young). Rep. 2 young (median lifespan = 23, *n* = 398, 256 censored), aged (median lifespan = 24, *n* = 398, 281 censored, *p* = 0.9538 compared to young). **E** Sec63-GFP and Hxk2 (loading control) immunoblots of young and aged control and *atg39∆ atg40*∆ cells during gametogenesis. Bar graphs show Sec63-GFP (cyan and magenta) and Free-GFP (Gray) levels normalized to Hxk2. Bar graphs depict mean, error bars show ± SD, 3 independent replicas. **F** Representative single z-slices of young and aged control and *atg39∆ atg40*∆ tetrads, respectively, visualizing Sec63-GFP (cyan) and histone Htb1-mCherry (magenta). Scale bar is 2 µm. **G** Background corrected mean fluorescent *SEC63-GFP* signal of young (*p* = 4.68 × 10^−37^) and aged (*p* = 1.46 × 10^−9^) control and *atg39∆ atg40*∆ tetrads respectively. Error bars show mean $\pm \,$SD, statistical significance was tested by two-sided Mann-Whitney *U* test. **H** Schematic depiction of impaired gametogenic rejuvenation in ER-phagy mutants. Violin plots indicate median (solid lines), 25th and 75th percentiles (dotted lines). Statistical significance for replicative lifespan differences was calculated using the Log-rank (Mantel-Cox) test, tick marks on survival curves represent censored events. *P* values are indicated (ns, *P* > 0.05, *P ≤ 0.05, **P ≤ 0.01, ***P ≤ 0.001, ****P ≤ 0.0001). Source data are provided as a Source Data file.

We sorted pre-gametogenic *atg39∆ atg40*∆ control cells into young and aged cell populations and determined efficiency of gametogenesis (Supplementary Fig. 9C–E), then analyzed the percentage of biotin labeled tetrads and replicative age of post-gametogenic cell populations (Fig. 6A, Supplementary Fig. 9F). Lifespan analysis confirmed again a similar significant and reproducible lower median replicative lifespan in gametes from aged precursor *atg39∆ atg40*∆ control cells compared to gametes from young precursor cells (Fig. 6B, Rep. 1, *p* = 8.6 × 10^−5^, Rep. 2, *p* = 4 × 10^−6^). Next, we sorted pre-gametogenic *atg39∆ atg40∆ pNDT80-ATG40* cells into young and aged cell populations, determined efficiency of gametogenesis and confirmed construct expression (Supplementary Fig. 9G–K). After completion of gametogenesis, we confirmed the percentage of biotinylated tetrads and mean replicative age of young and aged populations (Fig. 6C and Supplementary Fig. 9L). Excitingly, there was reproducibly no significant difference of replicative lifespan in gametes from aged precursor *atg39∆ atg40∆ pNDT80-ATG40* cells, (Fig. 6D, Rep.1, *p* = 0.4519, Rep.2, *p* = 0.9538). Finally, we quantified ER component dynamics during gametogenesis in young and aged wildtype and *atg39∆ atg40*∆ mutant cells (Supplementary Fig. 10A, B) by probing for endogenously-tagged Sec63-GFP, an abundant ER membrane protein present in both ER sheets and tubules. We found that Sec63-GFP accumulates during gametogenesis in young and aged cells lacking *atg39∆ atg40*∆, relative to wildtype cells, indicating that ER turnover through ER-phagy significantly impacts the amount of ER inherited by gametes (Fig. 6E–G, Supplementary Fig. 10C, D). Additionally, quantification of free GFP, a readout for ER-phagy, confirmed it to be lower in *atg39∆ atg40*∆ mutants, consistent with the expected ER-phagy defect in this background (Fig. 6E, Supplementary Fig. 10E). Together, these experiments demonstrate that complete resetting of replicative lifespan during gametogenesis is dependent on selective ER-phagy, possibly due to programmed turnover of specific ER species during gametogenesis (Fig. 6H).

## Discussion

Here, we present a high throughput microfluidics-based platform to identify the molecular mechanisms underlying the natural cellular rejuvenation that accompanies gametogenesis in budding yeast cells. This technique accelerates investigations of gametogenic rejuvenation efficiency and will be instrumental in revealing further mechanistic insights into the conserved process of gametogenic rejuvenation. Using our method, we corroborate the previous microdissection-based result that replicative lifespan is completely reset by gametogenesis^12^. Analysis of gamete lifespan in known short- and long-lived mutants reveals a large dynamic range of measurement using this approach. We found that in *sir2*∆ gametes from aged precursor cells, replicative lifespan was not fully reset due to a gametogenesis-independent mechanism. Finally, using this method, we revealed that while mitophagy appears to be dispensable for gametogenic rejuvenation, Atg39- and Atg40-dependent ER-phagy is necessary for gametogenic rejuvenation. The reconstitution of Atg40 during meiosis in this background is sufficient to completely reset replicative lifespan of gametes from aged precursor cells.

The custom microfluidic devices presented here, with an open U-trap design (adapted^21^), can capture a wide dynamic range of replicative lifespan with lower confinement of cells in space compared to other trap designs that can lead to negative effects on longevity due to compression of cells^65^. This open design presents the drawback that cells might undergo ejection prematurely before senescence can be observed, due to either a significant increase of cell size during the last few divisions of replicative life, or due to minor pressure aberrations in the fluidics system over time. To avoid biases when evaluating replicative lifespan of gametes, we counted every gamete that was loaded at the start of our time lapse and classified cells that ejected as censored subjects during analysis (see “Methods”). Following this approach, we can consistently and robustly measure replicative lifespan. We find the quantified increase of 33.33% in median lifespan of young *fob1*∆ gametes and 52% decrease for *sir2*∆ compared to lifespan of young wild-type gametes to be within the range of previous reports (Supplementary Table 1)^40,42,66,67^.

*SIR2* is a well-characterized conserved histone deacetylase of the Sirtuin family and regulates replicative lifespan by silencing the ribosomal DNA locus^40,41^. Deletion of *SIR2* leads to increased rDNA recombination and broad chromosome instabilities due to destabilization of the rDNA locus^68–70^. Gametes from aged *sir2*∆ precursor cells failed to reset replicative lifespan, a phenotype that could not be rescued by gametogenesis-specific expression of Sir2. Even though our rescue strategy may not fully recreate endogenous levels of Sir2 during gametogenesis, these results suggest that Sir2 does not play a direct role during gametogenic rejuvenation itself. We speculate that due to elevated recombination, aged *sir2*∆ cells could accumulate chromosomal aberrations that cannot be corrected during gametogenesis and are propagated to gametes, thus reducing their lifespan. Although, elevated pre-existing damage in *sir2*∆ cells is consistent with the substantially reduced efficiency of gametogenesis in aged cells in this background (Supplementary Fig. 4C)^67^, which was not generally observed in other backgrounds we analyzed, future studies directly investigating chromosome copy number and rDNA instability will be necessary to corroborate this model.

Autophagy of specific cellular components is known to be an important mechanism for cells to counteract age-associated damage and delay senescence^15,46–48^. The general autophagy machinery is upregulated during gametogenesis and is necessary for meiotic entry and progression^51,53^. We found that selective autophagy is a crucial pathway to mediate gametogenic rejuvenation and show specificity of this process, as ER-phagy but not mitophagy is important for gamete rejuvenation. This was a surprising result, given the substantial remodeling seen for mitochondria during gametogenesis^59,60^. It is possible that autophagy-independent mechanisms, such as potential sequestration of damaged mitochondria via the gametogenic uninherited compartment may ensure inheritance of healthy mitochondria^71,72^.

Developmentally programmed ER remodeling followed by selective autophagy of ER components regulates ER inheritance during yeast gametogenesis^62,63^. ER-specific autophagy receptors Atg40 and Atg39, localized on the cortical ER and perinuclear ER, respectively, control this process^64^. *atg40*∆ but not *atg39*∆ cells fail to degrade cortical ER components via autophagy during gametogenesis. However, *atg39*∆ cells show reduced autophagy-mediated loss of cortical and perinuclear ER proteins, suggesting functional redundancy with Atg40. Intriguingly, *atg40∆ atg39*∆ double mutants have an additive effect on ER protein degradation^62^. We show that aged *atg40∆ atg39*∆ precursor cells accumulate ER components during gametogenesis and fail to produce gametes with a fully reset lifespan. Loss of Rer1, a membrane protein retrieving receptor that maintains ER compartmentalization, has been shown to extend replicative lifespan in vegetative yeast through elevated autophagy triggered by ER stress^73,74^. We speculate that the rejuvenating defect of ER-phagy-deficient cells could be caused by a failure of meiotic clearance of damaged and aggregated ER components that accumulated in aged cells^64,75^. Determining the specific ER-associated damage that is cleared by ER-phagy during gametogenic rejuvenation and establishing a causative molecular link to the here described phenotype will be an interesting area to investigate in future studies.

ER-phagy is functionally conserved from yeast to metazoans and essential to maintain ER quality^76^. In mammals, mutations in ER-phagy receptor FAM134B cause hereditary sensory neuropathy and impaired ER turnover has been linked to neurodegeneration, including Parkinson’s disease^77,78^. Thus, ER quality control mediated by autophagy is a conserved mechanism to preserve cellular fitness. Our findings suggests that meiotic ER-phagy contributes to gamete rejuvenation by assuring ER integrity.

The technique presented here significantly increases throughput and makes it feasible to identify molecular factors that mediate cellular rejuvenation during the conserved process of gametogenesis. To gain more mechanistic and temporal understanding, this assay can easily be combined with auxin-inducible degradation of factors of interest and simultaneous imaging of established aging biomarkers in gametes that fail to completely rejuvenate. An important future goal will be to determine whether the rejuvenation factors that this platform reveals to control gamete quality are sufficient to extend replicative lifespan in mitotic cells, and to test if they have a conserved function in combating aging in other systems.

## Methods

### Yeast strains and plasmids

Standard molecular methods were used to transform and generate all yeast strains, which were derived from the SK1 background. Specific genotypes, plasmids and primers used to generate strains are listed in Supplementary information (Supplementary Tables 2–4). Deletions were generated by homology directed replacement of ORFs with selection cassettes^79,80^. The *pPMA2-PMA2-GFP* transgene plasmid was generated by Gibson assembly of the *PMA2* promoter and ORF region, amplified from genomic DNA, with an in frame *GFP*-containing *TRP1* single integration vector^81^. The *pTetO7.1-STE5* promoter swap was generated by amplifying a *NatR- pTetO7.1* fragment using primers that introduced homology for the *STE5* locus^39,79^. The *pRIM4-SIR2-V5* rescue construct was generated by Gibson assembly of the *SIR2* ORF region with *pRIM4* and *V5* containing *LEU2* single integration vector^81^. The *pNDT80-ATG40-V5* rescue construct was generated by Gibson assembly of the *ATG40* ORF region with *pNDT80* and *V5* containing *LEU2* single integration vector^81^. Generated plasmids were assembled using HiFi DNA assembly mix (NEbuilder HiFi DNA Assembly Master Mix, E2621L). *pRNR2-TetR-TUP1, PTetO7.1-TetR* adapted from^82^ was cloned into a *URA3* single integration vector by Gibson assembly^81^. Single integration plasmids were linearized with PmeI (New England Biolabs) mediated restriction digest before transformations and integrations were verified by PCR and sanger sequencing. Generated strains and plasmids are available upon request. The following alleles were constructed in previous studies: *atg39∆, atg40∆, ATG40-3V5, SEC63-eGFP*^62^*, HTB1-mCherry*^83^*, flo8∆, amn1*^*D368V*^.

### Media and growth conditions

Unless otherwise indicated, diploid cells were grown in YPD (1% yeast extract, 2% peptone, 2% glucose, 22.4 mg/L uracil, and 80 mg/L tryptophan). To induce gametogenesis cells were transferred to conditioned sporulation media containing 2% potassium acetate, amino acid supplements and ampicillin (40 mg/L adenine, 40 mg/L uracil, 10 mg/L histidine, 10 mg/L leucine and 10 mg/L tryptophan, 100 μg/mL ampicillin) at an OD_600_ of 0.8. All meiotic cultures were incubated on a shaker for 24 h at 30 °C to complete gametogenesis. To increase efficiency of gametogenesis, conditioned sporulation media was obtained by filter sterilizing a meiotic culture at 1.85 OD_600_ after 5 h at 30 °C and was stored at 4 °C. For immunoblotting saturated YPD 2% cultures were re-diluted to 0.2 OD_600_ in BYTA (1% yeast extract, 2% bacto tryptone, 1% potassium acetate, and 50 mM potassium phthalate) containing acetate as a carbon source and then grown overnight to saturation at 30 °C before transfer to unconditioned sporulation media without ampicillin at an OD_600_ of 1.85.

### Fluorescent microscopy

Bud scar staining and Pma2-GFP expression patterns during meiosis and gamete maturation were visualized using a DeltaVision Elite wide-field fluorescence microscope (GE Healthcare), equipped with a 60×/1.42 NA oil-immersion objective. Images were deconvolved with softWoRx imaging software (GE Healthcare). Time lapse images were acquired at 30 °C in an environmental chamber using a CellASIC ONIX Microfluidic Platform (Millipore) system.

Replicative lifespan timelapse imaging was performed at 30 °C in an environmental chamber using an ECHO Revolution (RON-K) microscope equipped with an Olympus 40x/1.4 NA oil-immersion objective.

### Age sorting of pre-gametogenic yeast cells

Young and aged cell populations were sorted by biotin-labeling of a founder population followed by bead-mediated magnetic sorting^34^. First, a patch of diploid cells was inoculated in YPD (2%) and grown overnight shaking at room temperature until saturation. Cells were then re-diluted in YPD (2%) to an OD_600_ of 0.2 and grown at 30 °C to an OD_600_ of 0.6–1. To increase yield, 2 × 8 OD_600_ units were harvested per genotype, washed 3x with PBS (pH 8), and then were transferred into PBS (pH 8) containing 2 mg/mL EZ-Link Sulfo-NHS-LC-biotin (ThermoFisher Scientific). Cells were vortexed for 1 min and incubated for 30 min at 4 °C on a nutator, washed 4 times with PBS + glycine (pH 8 + 100 mM glycine), and then washed once with YPD 2% before being added to two separate 500 mL flasks containing 50 mL YPD 2% + ampicillin (100 μg/mL) each. Biotin labeled cultures were aged for 15-19 h shaking at 30 °C. Aged cell cultures were pelleted, washed once with 20 mL PBS (pH 7.4 + 0.5% BSA), and then resuspended in 20 mL PBS (pH 7.4 + 0.5%) BSA containing 100 μL of anti-biotin magnetic beads (Miltenyi Biotechnology). Cells were incubated for 15 min at 4 °C and mixed regularly by inversion, washed once with 20 mL PBS (pH 7.4 + 0.5% BSA) and then resuspended in 5 mL PBS (pH 7.4 + 0.5% BSA). Using a QuadroMacs sorter, both suspensions were applied to a column each. The first flowthrough from each column was collected, combined, and used as the young cell population. Each column was washed 4 times with 3 mL PBS (pH 7.4 + 0.5% BSA) before eluting into a second column, to increase purity. The second columns were washed three times with 3 mL PBS (pH 7.4 + 0.5% BSA), cells were eluted, combined, and subsequently used as the aged cell population.

### Immunostaining and gametogenesis

Young and aged cell suspensions were washed twice with 12 mL PBS (pH 7) and resuspended in 4 mL PBS (pH 7). To visualize bud scars and biotinylated cells 4 μL of Wheat Germ Agglutinin, Alexa Fluor^TM^ 350 conjugate (1 μg/mL, ThermoFisher Scientific) and 4 μL streptavidin Alexa Fluor^TM^ 546 conjugate (1 μg/mL, ThermoFisher Scientific) was added respectively and incubated for 15 min at room temperature in the dark. Cells where washed (3x) with 12 mL of unconditioned sporulation media and resuspended to an OD_600_ of 0.8 in conditioned sporulation media + ampicillin (100 μg/mL). 0.1 OD_600_ units of cells was fixed in 3.7% formaldehyde to assess age before gametogenesis. Cultures were incubated for 24 h at 30 °C in the dark to complete gametogenesis and a 0.1 OD_600_ units of cells were used directly to count tetrad formation and another fraction of cells were fixed in 3.7% formaldehyde to assess age post-gametogenesis by imaging and counting biotinylated cells as well as bud scars.

### Tetrad separation into single gametes and re-growth

Post-gametogenic age sorted cultures were pelleted and resuspended in 900 μL H_2_O, 100 μL Zymolyase (1 mg/mL) and 2 μL B-mercaptoethanol and the suspension was incubated for 3 h on a nutator at 30 °C. Cells were washed (1×) with H_2_O and resuspended in 1 mL H_2_O followed by repeated sonication (6 × 20 s) incubating the cells 2–3 min on ice in-between sonication cycles. Upon tetrad separation, separated gametes were filtered (30 µm Pre-separation filter, militenyibiotec) and diluted in YPD 2% + ampicillin (100 μg/mL) to an OD_600_ of 0.8. After 3 h at incubation on a nutator at 30 °C, 1 mL of cell suspension was aliquoted, sonicated briefly for 5 s and filtered before loading into the microfluidics system.

### Microfluidics loading and timelapse imaging of replicative lifespan

Microfluidics devices (Supplementary Fig 2A–D adapted from^21^;18++ chips from iBiochips) were setup using the manufacturer’s recommendations on an ECHO Revolution fluorescence microscope in the inverted position with the environmental chamber set to 30˚C. Briefly, 20 mL syringes (Becton, Dickinson and Company) were filled with ~15 mL of YPD 2% + ampicillin (100 μg/mL) + 1% Pluronic F-127 (P2443, Sigma), and then connected to a Clay Adams Intramedic Luer-Stub Adapter (Becton, Dickinson and Company). Non-DEHP Medical Grade Tubing (iBiochips, MTB-100, “ID = 0.202”, “OD = 0.060”, “wall = 0.20”) equipped with a hollow stainless-steel pin (iBiochips, HSP-200) was then used to connect the media syringe to the imaging units using the “media loading” ports. “Cell loading” ports were plugged with solid stainless-steel pins (iBiochips, SSP-200) while imaging units were primed with media for at least 2 h with a flow rate of 2.4 μL/min using micropumps (^kd^Scientific, 788200 and 788300). After all units were filled with media, pumps were stopped an hour prior to cell loading. For cell loading, 5 mL syringes (Becton, Dickinson and Company) with Clay Adams Intramedic Luer-Stub Adapter (Becton, Dickinson and Company) were filled with 1 mL of cells diluted to an OD_600_ of 0.8 in YPD 2% + ampicillin (100 μg/mL). Solid stainless-steel pins were then removed from the “cell loading” ports and tubing and open stainless-steel pins were used to connect the syringe containing cells with the microfluidic device. Cells were loaded manually and, once sufficient cells were loaded, the “cell loading” ports were plugged with solid stainless-steel pins and media flow was resumed at 2.4 μL/min.

Timelapse imaging was performed at 30 °C in an environmental chamber using an Olympus 40×/1.4 NA oil-immersion objective with intervals of 15–20 min for 72 h. The x-, y-, and z-motors were set to medium speed; however, varying stage speeds caused sporadic differences in imaging intervals. Thus, the recorded acquisition time provides accurate timing information. For each chamber, 2 × 27 images and three z-slices per position with 3–8 μm spacing, to compensate for focal plane differences across a single unit, were acquired. Autofocus was set across a 20 μm range with 0.2 μm spacing using the “Course-Fine” setting (Threshold = 4, Contrast Limit = 4). The XY Stage and Z Motor were both set to medium speed and Hyperscan was enabled for the “BF” channel using “Y-Axis” Axis Priority and “Bi” Scanning Pattern.

### Quantification of replicative age and purity of sorted cells

Z-stacks (36 slices, 0.2 µm spacing) of fixed young and aged cells/tetrads were acquired on a DeltaVision Elite wide-field fluorescence microscope (GE Healthcare), using a 60×/1.42 NA oil-immersion objective and deconvolved with softWoRx imaging software (GE Healthcare). To reliably count number of bud scars, images were cropped, 3D projected and counted using a custom semi-automated Fiji/ImageJ script (ImageJ2, V. 2.14.0/1.54 m)^84,85^. Max intensity Z-projections were generated and displayed as examples in figures.

### Quantification of gamete replicative lifespan

After the movie was completed, the ECHO batch exporter tool was used to export stitched TIFF images. Individual TIFF images were converted to 8-bit using Fiji/ImageJ (ImageJ2, V. 2.14.0/1.54 m)^84^, then full imaging unit time-lapse could be opened using the “Bio Formats Import” tool with the “Group files with similar names” box checked. Single Pma2-GFP positive cells (gametes) were identified by eye and cropped both in the FITC image and the brightfield time course using a custom semi-automated Fiji/ImageJ script^85^. Next using a semi-automated Fiji/ImageJ script^85^, the replicative lifespan of Pma2-GFP positive cells (gametes) identified in the FITC image were counted, verifying identity by comparing the FITC and brightfield time-lapse for each single gamete. If a cell ejected from the U-trap before senescence was observed, this was noted and treated as a censored subject for quantification. For most experiments, counts from two technical replicates were concatenated unless otherwise indicated. Although throughput to quantify gametogenic reset of lifespan is significantly higher than with previous methods, cell divisions of each gamete must still be counted manually by a trained scientist rendering data analysis as a current bottleneck of the presented method.

### Quantification of mean fluorescence

Fluorescence images were quantified using a custom semi-automated Fiji/ImageJ (ImageJ2, V. 2.14.0/1.54 m)^84,85^ workflow. Z-stacks were converted into sum-intensity projections and the outline of each tetrad was manually defined using the polygon selection tool. Background fluorescence signal was determined by averaging measurements of multiple cell-free regions for each image. Background-corrected mean fluorescence was calculated as mean fluorescence minus average background fluorescence. Statistical significance was assessed using a two-tailed Mann-Whitney *U* test.

### Immunoblotting

For each sample, 1.85-10 OD_600_ units were harvested and pelleted from either log-phase YPD 2%, saturated YPD 2%, saturated BYTA or indicated timepoints upon transfer to sporulation media. For each sample, 1–2 mL 5% trichloroacetic acid was added and cells were incubated for ≥10 min at 4 °C min, washed (1x) with acetone, and pellets was dried overnight. For protein extraction, ~100 μL glass beads and 100 μL of lysis buffer (50 mM Tris-HCl pH 8.0, 1 mM EDTA, 3 mM DTT, 2.2 mM PMSF, 1x complete EDTA-free inhibitor cocktail (Roche)) were added to the dried pellets and transferred to a Mini-Beadbeater-96 (BioSpec) for 5 min. Fifty μL of 3X SDS sample buffer (187.5 mM Tris pH 6.8, 6% β-mercaptoethanol, 30% glycerol, 9% SDS, 0.05% bromophenol blue) was added and samples were for boiled for 5 min at 95 °C. Polyacrylamide gel electrophoresis (4–12% Bis-Tris Bolt gels, Thermo Fisher) and semi-dry transfer (Trans-Blot Turbo Transfer System, Bio-Rad) onto nitrocellulose membranes apparatus was used to separate and visualize proteins respectively. Odyssey PBS Blocking Buffer (LI-COR Biosciences) was used to block membranes (90 min, room temperature), and membranes were incubated overnight at 4 °C with either a mouse anti-3v5 (R960-25; RRID: AB_2556564, Thermo Fisher) antibody at 1:2000 in PBS + 0.1% Tween-20 or a mouse anti-GFP (JL8, Takara) at 1:2000 in PBS + 0.1% tween and a rabbit anti-Hx2k (RRID:AB_219918, 100–4159, Rockland) at 1:20,000 in PBS + 0.1% tween. Membranes were washed with PBS + 0.1% Tween-20 (4x, 5 min each) and incubated with secondary antibody solutions containing anti-mouse conjugated to IRDye 800CW at a 1:15,000 dilution (RRID:AB_621847, 926–32212, LI-COR Biosciences) and an anti-rabbit conjugated to IRDye 680RD at a 1:15,000 dilution (RRID:AB_10956166, 926–68071, LI-COR Biosciences) in PBS + 0.1% Tween-20. Membranes were imaged using an Odyssey CLx system (LI-COR Biosciences).

### Statistical analysis

Prism Graphpad (version 10.1.0) was used to plot and analyze data. Prism Graphpad was used to calculated mean and standard deviation of replicative ages and to assess statistical significance in differences between replicative lifespans using the Log-rank (Mantel-Cox) test. Two-sided Mann-Whitney *U* test was used to calculate statistical significance of background corrected mean fluorescent signal. A Python script was used to calculate 95% confidence intervals using the Wilson score method.

### Reporting summary

Further information on research design is available in the Nature Portfolio Reporting Summary linked to this article.

## Supplementary information

Supplementary Information

Reporting Summary

Transparent Peer Review file

## Source data

Source Data File

## Data Availability

The quantitative data generated in this study and underlying the figures are provided in the Supplementary Information and the Source Data file. Raw microscopy data are available from the corresponding authors upon request due to the large file sizes and number of files involved. Data previously published and used in this study are available in the original publication (Cheng et al.)^43^. Source data are provided with this paper.

## Code availability

Custom Fiji/ImageJ macros used for microscopy image processing and analysis in this study have been deposited in Zenodo and are publicly available at https://doi.org/10.5281/zenodo.22004193^85^.
