# Supplementary material for: A role for selective ER autophagy in gametogenic rejuvenation revealed by high-throughput lifespan profiling
Source: Nat Commun. 2026 Sep 29;17:11215. doi: 10.1038/s41467-026-77993-0 (PMC13720134; doi:10.1038/s41467-026-77993-0)
Supplement: Supplementary file 1 [file 41467_2026_77993_MOESM1_ESM.pdf]

Supplementary information *Spiri et al. 2026*:

A role for selective ER autophagy in gametogenic rejuvenation revealed by high-throughput lifespan profiling

Silvan Spiri[1]*, Tina Lynn Sing[1], Nhi Phung[1], Jay S Goodman[1], Elçin Ünal[1,2,3]* & Gloria Ann Brar[1,2,3]*

[1]Department of Molecular and Cell Biology, University of California, Berkeley, Berkeley, CA, 94720, USA
[2]California Institute for Quantitative Biosciences (QB3), University of California, Berkeley, CA, 94720, USA
[3]Center for Computational Biology, University of California, Berkeley, Berkeley, CA 94720, USA

*Correspondence: silvan.spiri@berkeley.edu, elcin@berkeley.edu, gabrar@berkeley.edu

Content:

Supplementary Figure 1
Supplementary Figure 2
Supplementary Figure 3
Supplementary Figure 4
Supplementary Figure 5
Supplementary Figure 6
Supplementary Figure 7
Supplementary Figure 8
Supplementary Figure 9
Supplementary Figure 10
Supplementary Table 1
Supplementary Table 2
Supplementary Table 3
Supplementary Table 4
Supplementary Figures Source Data
Supplementary References

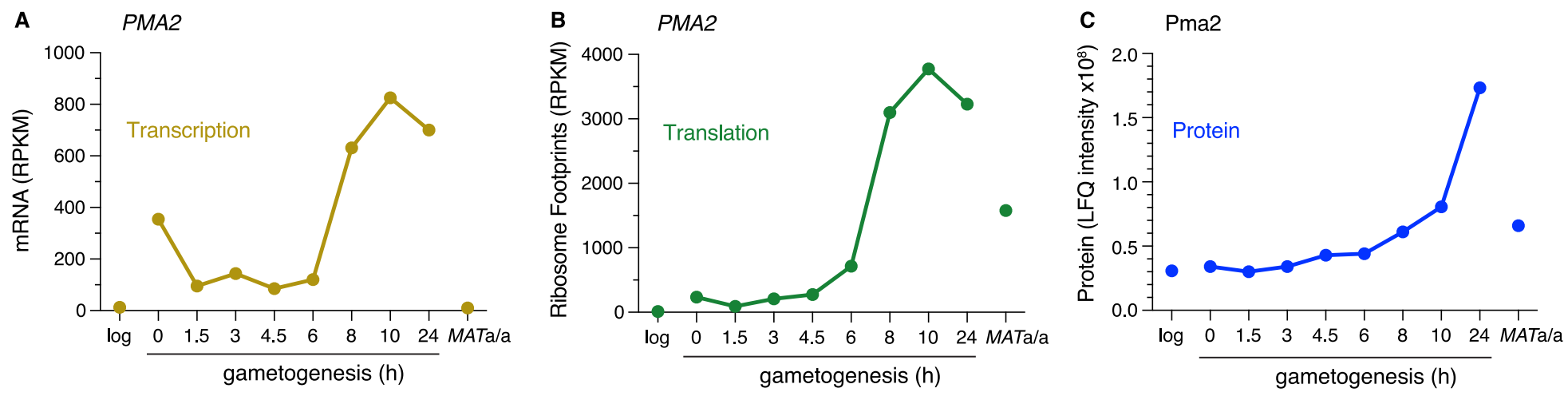

Supplementary Figure 1. *PMA2* translation and protein levels are upregulated during late gametogenesis. Expression of *PMA2* either during log-phase vegetative growth in 2% glucose containing media (log), the indicated time in gametogenesis inducing media (h), or in control diploids that cannot undergo gametogenesis (*MAT*a/a) plotted from previously generated meiotic datasets[1]. **A** *PMA2* transcript levels (RNA-seq). **B** *PMA2* translation levels (ribosome-protected footprints). **C** Pma2 protein levels (mass spectrometry).

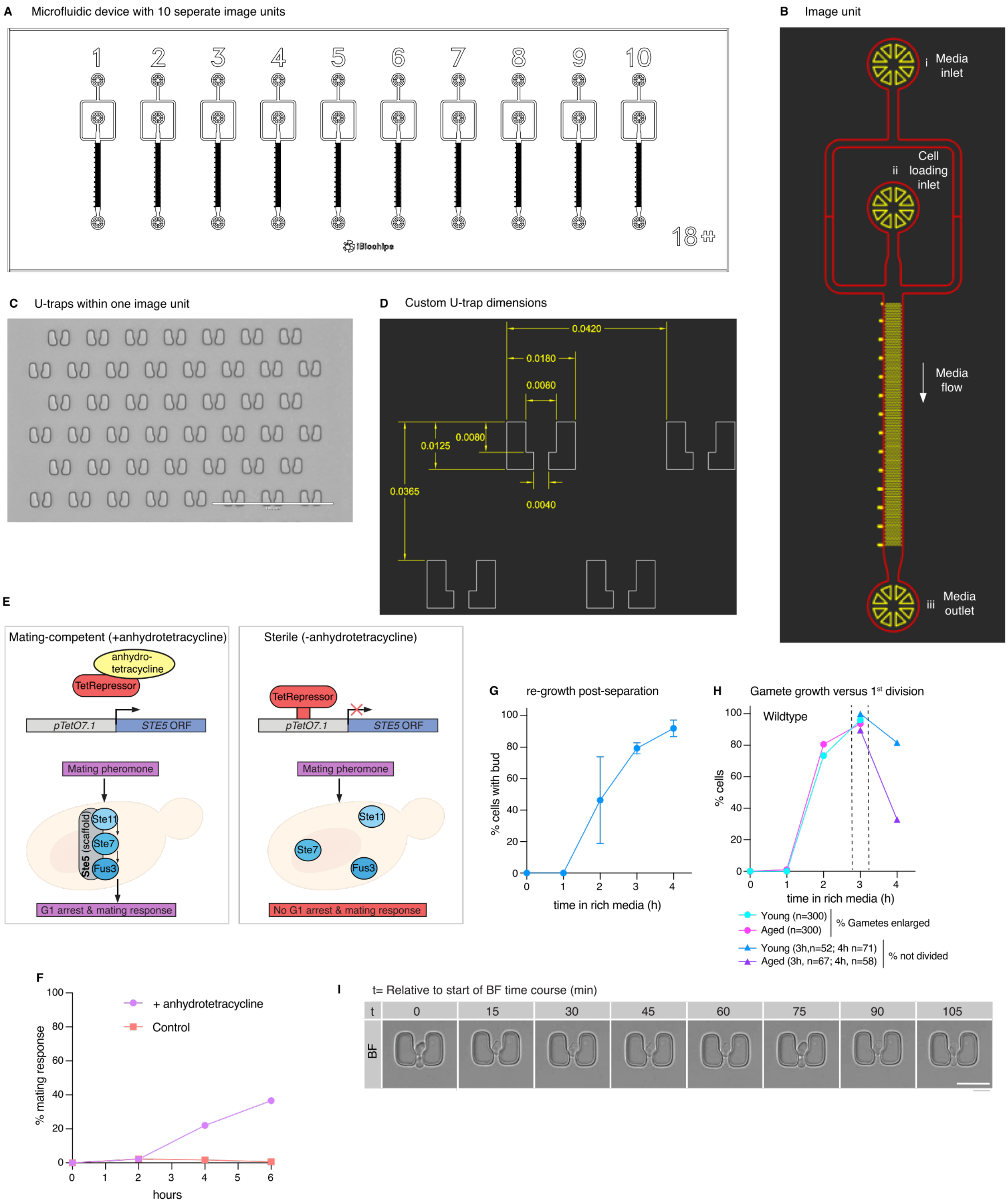

Supplementary Figure 2. Custom-built U-trap microfluidic devices with specific dimensions for vegetatively growing haploid SK1 cells. **A** Schematic view of one microfluidic device with 10 separate imaging units. **B** Schematic overview of one unit with (i) media inlet; cell loading inlet (ii) and waste outlet (iii). During the experiment media flows from media inlet (i) to media outlet (iii) while cell loading inlet is plugged with a solid stainless-steel pin. **C** Image of U-traps and spacing in one unit, scale bar is 100 μm. **D** Custom developed SK1 specific dimensions of trap width and height (8 μm), exit channel width (4 μm), and spacing between traps (24 μm). **E** Schematic depiction of genetic system to achieve constitutive sterility. **F** Quantification of mating response scoring zygote formation upon 1:1 mixing of *MAT***a** and *MAT*α cells (n=300 for each timepoint). **G** Monitoring re-growth of separated gametes in glucose containing liquid culture by scoring emergence of buds over time (n=100 each, 3 replicas), dot shows mean, error bars show SD. **H** Monitoring of growth and cell divisions of separated gametes in glucose

containing liquid culture by scoring enlarged gametes (graphs depicted with dots, n=300 each) and assessing number of bud scars (graphs depicted with triangles, n=52 (3h), 71 (4h) for young and n=67 (3h) and 58 (4h for aged cells). **I** Representative trapped gamete dividing twice over the time of two hours, scale bar is 5 μm.
Source data are provided as a Source Data file.

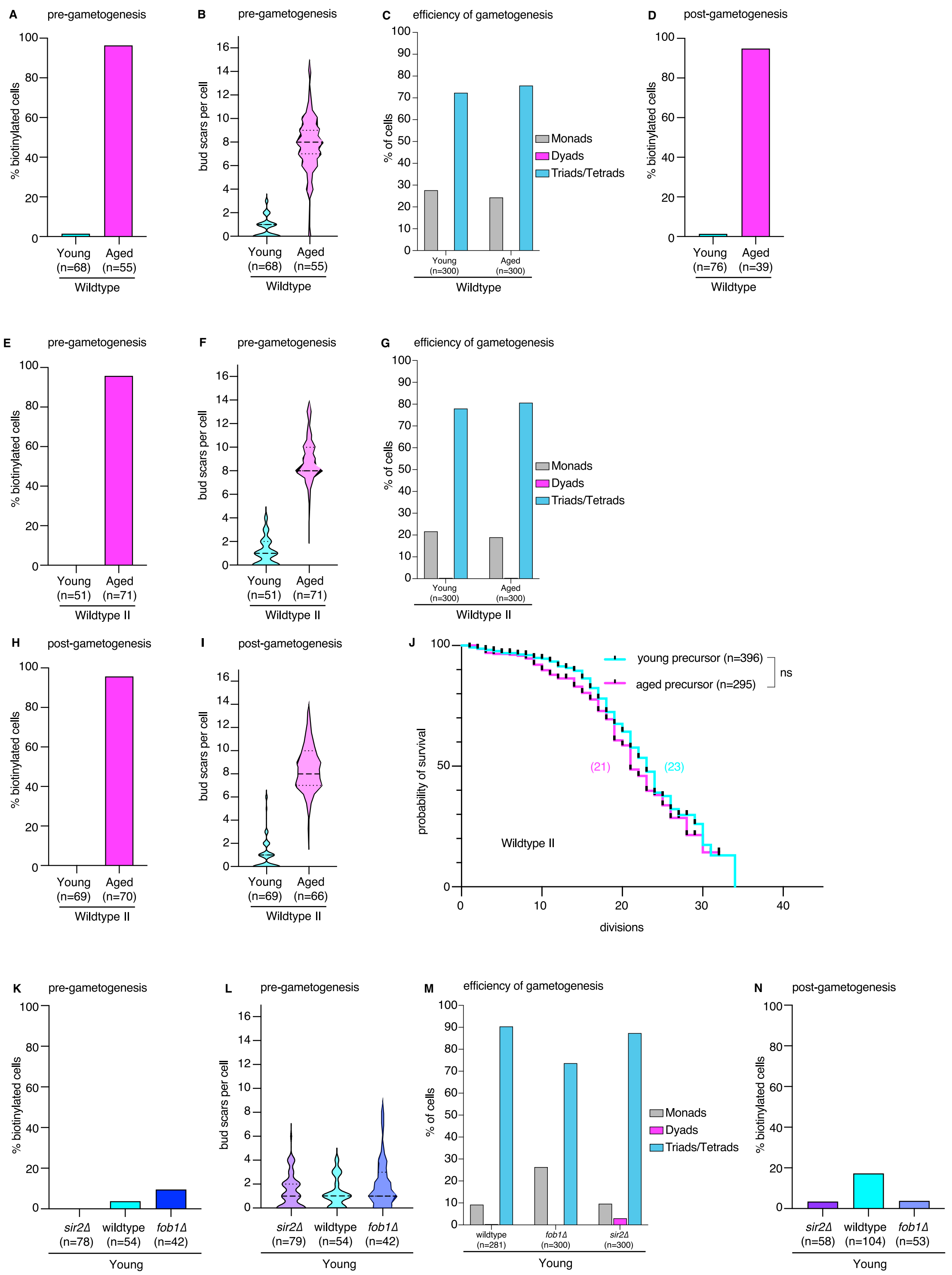

Supplementary Figure 3. Supporting data for wildtype gametes completely reset replicative lifespan during gametogenesis. **A** Fraction of biotinylated cells in young (1.5%, 95% CI: 0.3–7.9%, n=68) and aged (96.4%, 95% CI: 87.7–99.0%, n=55) wildtype cell populations, same data as shown in Fig. 2F for Rep. 3. **B** Quantification of replicative age by assessing number of bud scars in young (0.72$\pm$ 0.84, mean $\pm$ SD, n=68) and aged (7.8 $\pm$ 2.2, mean $\pm$ SD, n=55) wildtype cell populations, same data as shown in Fig. 2E for Rep. 3. **C** Fraction of tetrads in young (72.3%, 95% CI: 67.0–77.1%, n=300) and aged (75.7%, 95% CI: 70.5–80.2% n=300) post-gametogenic cell populations, same data as shown in Fig. 2G for Rep. 3. **D** Fraction of biotinylated tetrads in young (1.3%, 95% CI: 0.2–7.1%, n=76) and aged (94.9%, 95% CI: 83.1–

98.6%, n=39) wildtype populations after completion of gametogenesis, same data as shown in Fig. 2H for Rep. 3. **E** Fraction of biotinylated cells in young (none, n=51) and aged (95.8%, 95% CI: 88.3–98.6%, n=71) wildtype II cell populations. **F** Quantification of replicative age by assessing number of bud scars in young (1 ± 1.1, mean ± SD, n=51) and aged (8.8 ± 1.7, mean ± SD, n=71) wildtype II cell populations. **G** Fraction of tetrads in young (78%, 95% CI: 73.0–82.3%, n=300) and aged (80.7%, 95% CI: 75.8–84.7% n=300) post-gametogenic wildtype II cell populations. **H** Fraction of biotinylated tetrads in young (none, n=69) and aged (95.7%, 95% CI: 88.1–98.5%, n=70) wildtype II populations after completion of gametogenesis. **I** Quantification of replicative age by assessing number of bud scars in young (0.94± 1.3, mean ± SD, n=69) and aged (8.4± 1.9, mean ± SD, n=66) wildtype II gamete populations. **J** Quantification of replicative lifespan of young (median lifespan=23, n=396, 274 censored subjects) and aged (median lifespan=21, n=295, 205 censored subjects, p=0.1341 compared to young) wildtype II gametes. **K** Fraction of biotinylated cells in young *sir2Δ* (none, n=78), wildtype (3.7%, 95% CI: 1–12.5%, n=54) and *fob1Δ* (9.5%, 95% CI: 3.8– 22.1%, n=42) cell populations. **L** Quantification of replicative age by assessing number of bud scars in young *sir2Δ* (1.3± 1.4, mean ± SD, n=79), wildtype (1.2 ± 1.2, mean ± SD, n=54) and *fob1Δ* (1.9 ± 1.8, mean ± SD, n=42) cell populations. **M** Fraction of tetrads in young *sir2Δ* (87.3 %, 95% CI: 83.1– 90.6%, n=300), wildtype (90.4 %, 95% CI: 86.4 – 93.3%, n=281) and *fob1Δ* (73.7%, 95% CI: 68.4–78.3%, n=300) post-gametogenic cell populations. **N** Fraction of biotinylated tetrads in young *sir2Δ* (3.4%, 95% CI: 1.0–11.7%, n=58), wildtype (17.3%, 95% CI: 11.2–25.7%, n=104) and *fob1Δ* (3.8%, 95% CI: 1.0–12.8%, n=53) populations after completion of gametogenesis.

Bar graphs show mean, confidence intervals were calculated using the Wilson score method. Solid lines in violin plots indicate the median, dotted lines indicate the 25th and 75th percentiles. Statistical significance for differences between replicative lifespans was calculated using the Log-rank (Mantel-Cox) test, tick marks on survival curves represent censored events. P values are indicated (ns, P>0.05, *P≤0.05, **P≤0.01, ***P≤0.001, ****P≤0.0001). Source data are provided as a Source Data file.

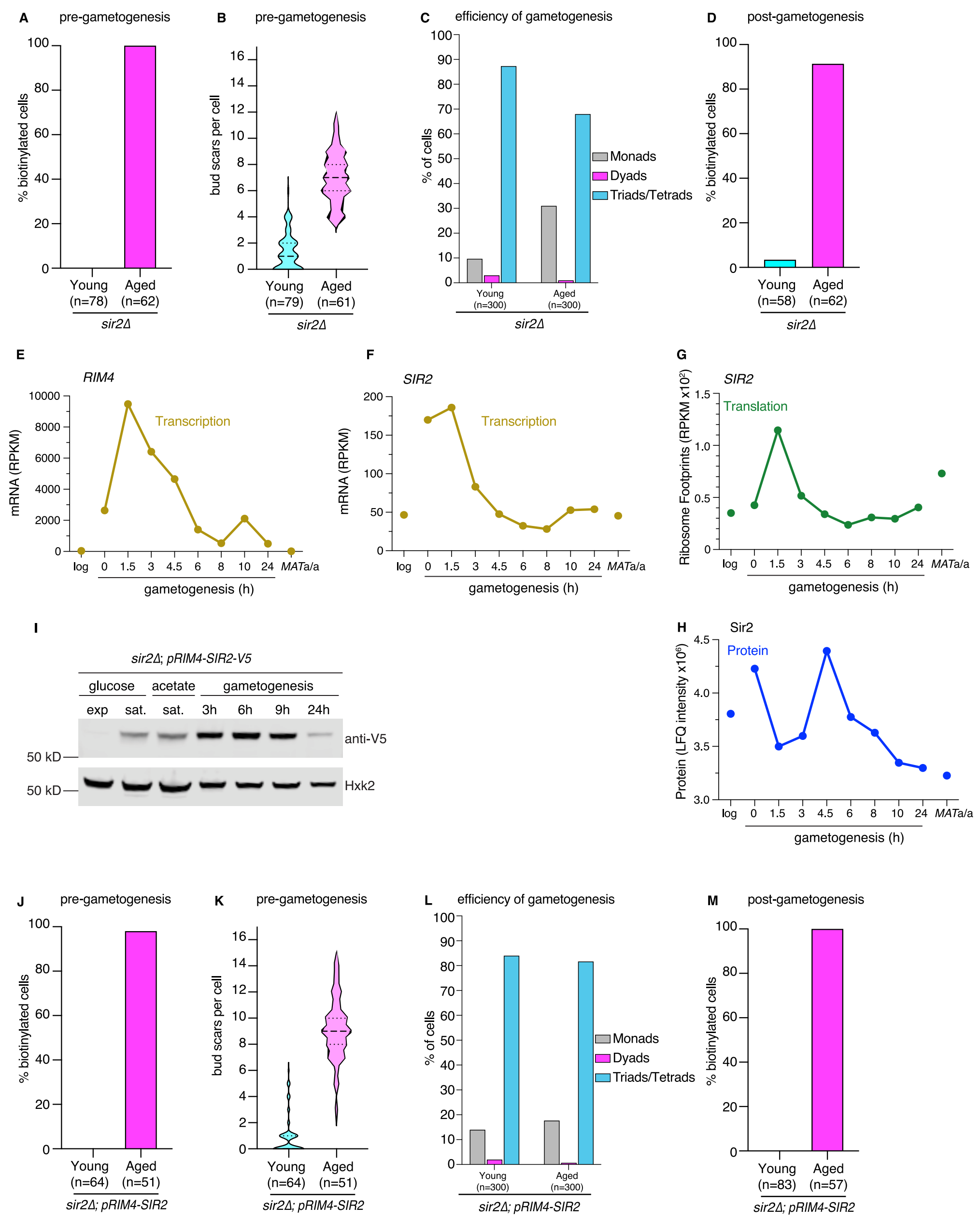

Supplementary Figure 4. Supporting data for *sir2Δ* mutants and gametogenesis specific Sir2 expression both fail to reset replicative lifespan of gametes. **A** Fraction of biotinylated cells in young (none, n=78, same data as shown in Supplementary Fig. 3K) and aged (all, n=78) *sir2Δ* cell populations. **B** Quantification of replicative age by assessing number of bud scars in young (1.3± 1.4, mean ± SD, n=79, same data as shown in Supplementary Fig. 3L) and aged (7± 1.9, mean ± SD, n=61) *sir2Δ* cell populations. **C** Fraction of tetrads in young (87.3% 95% CI: 83.1–90.6% n=300, same data as shown in Supplementary Fig. 3M) and aged (68.0% (95% CI: 62.5–73.0%, n=300) post-gametogenic *sir2Δ* cell populations. **D** Fraction of biotinylated tetrads in young (3.4%, 95% CI: 1.0–11.7%, n=58, same data as shown in Supplementary Fig. 3N) and aged (91.9%, 95% CI: 82.5–96.5%, n=62) *sir2Δ* populations after completion of gametogenesis. **E-H** Expression levels either during log-phase vegetative growth in 2% glucose containing media (log), the indicated time in gametogenesis inducing media (h), or in control diploids that cannot undergo gametogenesis (*MAT***a/a**) plotted from previously generated meiotic datasets[1]. **E** *RIM4* transcript levels (RNA-seq) **F** *SIR2* transcript levels (RNA-seq). **G** *SIR2* translation levels (ribosome-protected footprints). **H** Sir2 protein levels

(mass spectrometry). **I** Immunoblot to quantify Sir2-V5 levels driven by *RIM4* promoter fragment (anti-V5) and loading control (Hxk2) in growth media containing ether 2% glucose (glucose) or acetate (acetate) and at different timepoints upon transfer to gametogenesis-inducing media (gametogenesis). **J** Fraction of biotinylated cells in young (none, n=64) and aged (98.0%, 95% CI: 89.7–99.7%, n=51) *pRIM4-SIR2 sir2Δ* cell populations. **K** Quantification of replicative age by assessing number of bud scars in young (0.98$\pm$ 1.5, mean $\pm$ SD, n=64) and aged (9.1$\pm$ 2.2 (mean $\pm$ SD, n=51) *pRIM4-SIR2 sir2Δ* cell populations. **L** Fraction of tetrads in young (84 %, 95% CI: 79.4–87.7%, n=300) and aged (81.7%, 95% CI: 76.9–85.6%, n=300) post-gametogenic *pRIM4-SIR2 sir2Δ* cell populations. **M** Fraction of biotinylated tetrads in young (none, n=83) and aged (all, n=57) *pRIM4-SIR2 sir2Δ* populations after completion of gametogenesis.
Bar graphs show mean, confidence intervals were calculated using the Wilson score method. Solid lines in violin plots indicate the median, dotted lines indicate the 25th and 75th percentiles. Source data are provided as a Source Data file.

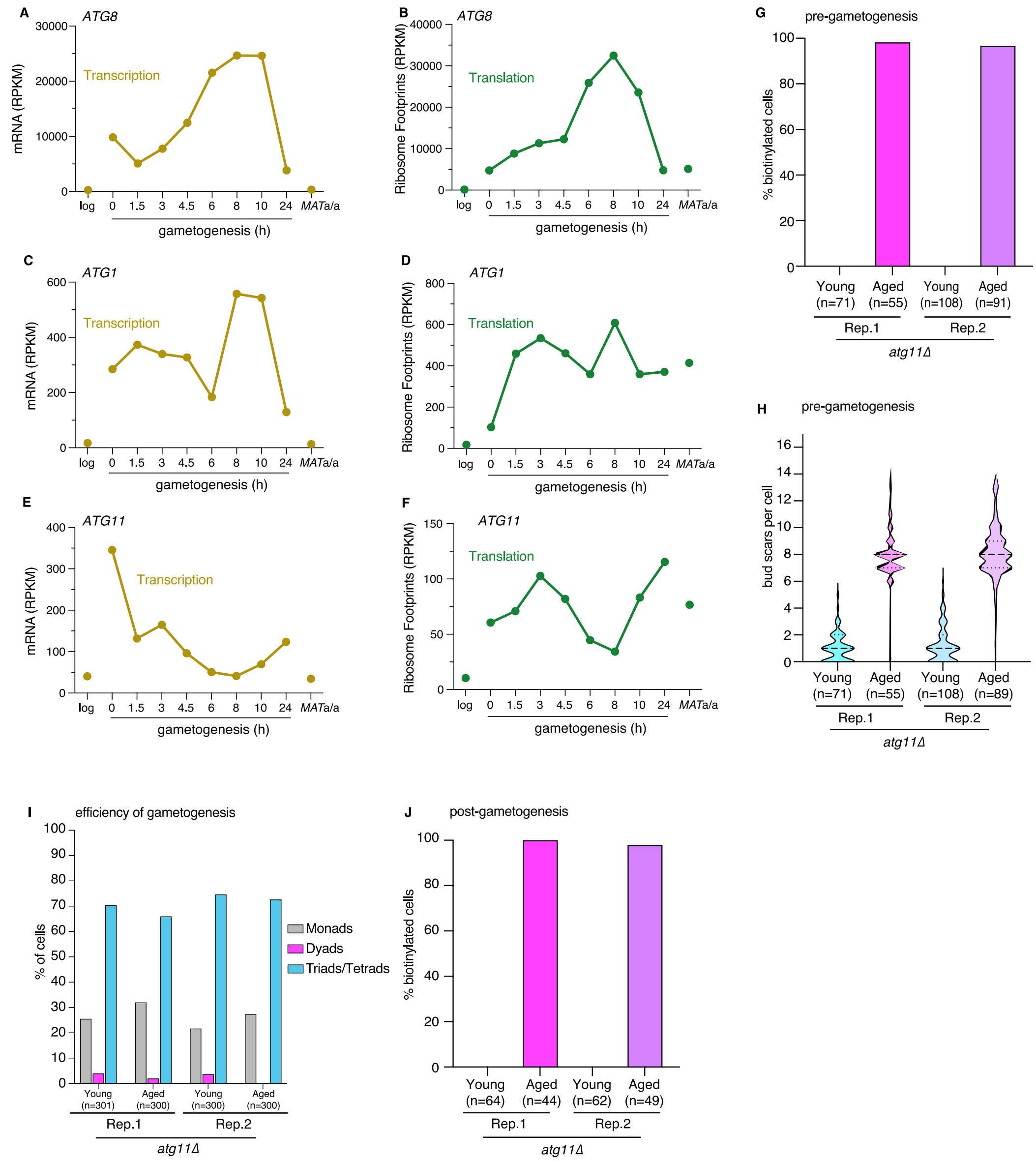

Supplementary Figure 5. Supporting data for *atg11Δ* mutants fail to reset replicative lifespan of gametes. **A-F** Expression of autophagy factors either during log-phase vegetative growth in 2% glucose containing media (log), the indicated time in gametogenesis inducing media (h), or in control diploids that cannot undergo gametogenesis (*MAT***a**/**a**) plotted from previously generated meiotic datasets[1]. **A** *ATG8* transcript levels (RNA-seq). **B** *ATG8* translation levels (ribosome-protected footprints). **C** *ATG1* transcript levels (RNA-seq). **D** *ATG1* translation levels (ribosome-protected footprints). **E** *ATG11* transcript levels (RNA-seq). **F** *ATG11* translation levels (ribosome-protected footprints). **G** Fraction of biotinylated cells in *atg11Δ* cell populations: Replicate 1 (Rep. 1) young (none, n=71) and aged (98.2%, 95% CI: 90.4–99.7%, n=55); Replicate 2 (Rep. 2) young (none, n=108) and aged (96.7%, 95% CI: 90.8–98.9%, n=91). **H** Quantification of replicative age by assessing number of bud scars in *atg11Δ* cell populations: Rep. 1 young (1 ± 1.1, mean ± SD, n=55) and aged (7.9± 1.9, mean ± SD, n=71); Rep. 2 young (1.2 ± 1.3, mean ± SD, n=108) and aged (8.2± 1.9, mean ± SD, n=89). **I** Fraction of tetrads in post-gametogenic *atg11Δ* cell populations: Rep.1 young (70.4%, 95% CI: 65.0–75.3%), n=301) and aged (66.0%, 95% CI: 60.5–71.1%, n=300); Rep. 2 young (74.7%, 95% CI: 69.5–79.3%, n=300) and aged (72.7%, 95% CI: 67.4–77.4%, n=300). **J** Fraction of biotinylated tetrads in *atg11Δ* populations after completion of gametogenesis:

Rep.1 young (none, n=64) and aged (all, n=44; Rep. 2 young (none, n=62) and aged (98%, 95% CI: 89.3–99.6%, n=91all, n=49).
Bar graphs show mean, confidence intervals were calculated using the Wilson score method.
Solid lines in violin plots indicate the median, dotted lines indicate the 25th and 75th percentiles.
Source data are provided as a Source Data file.

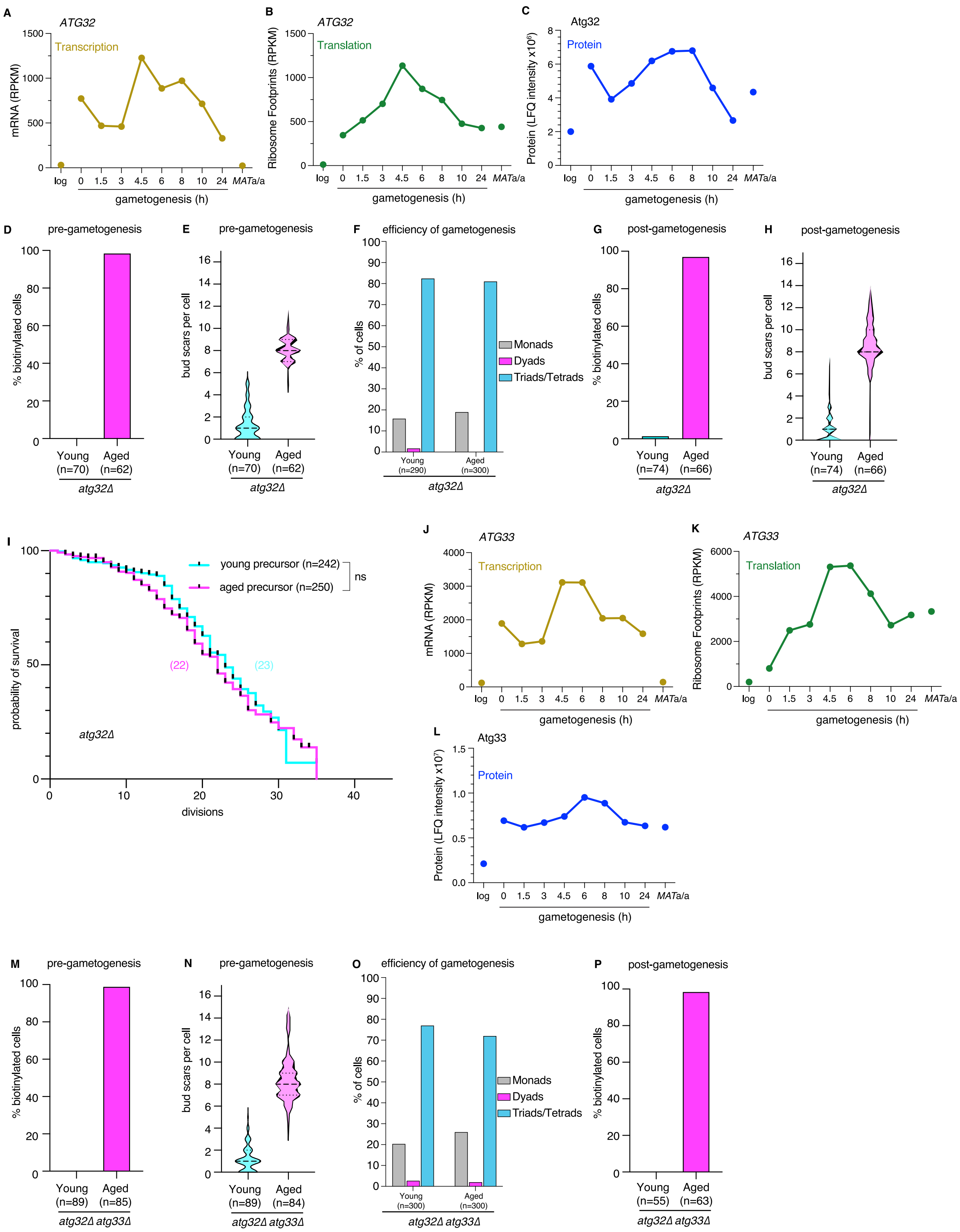

Supplementary Figure 6. Supporting data for *atg32Δ* mutants and *atg32Δ atg33Δ* double mutants completely reset replicative lifespan of gametes. **A-C & J-L** Expression levels either during log-phase vegetative growth in 2% glucose containing media (log), the indicated time in gametogenesis inducing media (h), or in control diploids that cannot undergo gametogenesis (*MAT***a**/**a**) plotted from previously generated meiotic datasets[1]. **A** *ATG32* transcript levels (RNA-seq). **B** *ATG32* translation levels (ribosome-protected footprints). **C** Atg32 protein levels (mass spectrometry). **D** Fraction of biotinylated cells in young (none, n=70) and aged (98.4%, 95% CI: 91.4–99.7%, n=62) *atg32Δ* cell populations. **E** Quantification

of replicative age by assessing number of bud scars in young (1.3± 1.4, mean ± SD, n=70) and aged (8.1± 0.98, mean ± SD, n=62) *atg32Δ* cell populations. **F** Fraction of tetrads in young (82.4%, 95% CI: 77.6–86.4%), n=290) and aged (81.0%, 95% CI: 76.2–85.0%, n=300) post-gametogenic *atg32Δ* cell populations. **G** Fraction of biotinylated tetrads in young (1.4%, 95% CI: 0.2–7.3%, n=74) and aged 97.0% (95% CI: 89.6–99.2%, n=66) *atg32Δ* populations after completion of gametogenesis. **H** Quantification of replicative age by assessing number of bud scars in young (0.93± 1.3, mean ± SD, n=74) and aged (8.7± 2, mean ± SD, n=66) *atg32Δ* gamete populations. **I** Quantification of replicative lifespan of young (median lifespan=23, n=242, 151 censored subjects, 1 technical replicate) and aged (median lifespan=22, n=250, 151 censored subjects, p=0.2691) *atg32Δ* gametes. **J** *ATG33* transcript levels (RNA-seq). **K** *ATG33* translation levels (ribosome-protected footprints). **L** Atg33 protein levels (mass spectrometry). **M** Fraction of biotinylated cells in young (none, n=89) and aged (98.8%, 95% CI: 93.6–99.8%, n=85) *atg32Δ atg33Δ* double mutant cell populations. **N** Quantification of replicative age by assessing number of bud scars in young (1.2± 1.1, mean ± SD, n=89) and aged (8.4± 2, mean ± SD, n=84) *atg32Δ atg33Δ* double mutants. **O** Fraction of tetrads in young (77.0%, 95% CI: 71.9–81.4%, n=300) and aged (72.0%, 95% CI: 66.7–76.8%, n=300) post-gametogenic *atg32Δ atg33Δ* cell populations. **P** Fraction of biotinylated tetrads in young (none, n=55) and aged (98.4%, 95% CI: 91.5–99.7%, n=63) *atg32Δ atg33Δ* populations after completion of gametogenesis.
Bar graphs show mean, confidence intervals were calculated using the Wilson score method. Solid lines in violin plots indicate the median, dotted lines indicate the 25th and 75th percentiles. Statistical significance for differences between replicative lifespans was calculated using the Log-rank (Mantel-Cox) test, tick marks on survival curves represent censored events. P values are indicated (ns, P>0.05, *P≤0.05, **P≤0.01, ***P≤0.001, ****P≤0.0001). Source data are provided as a Source Data file.

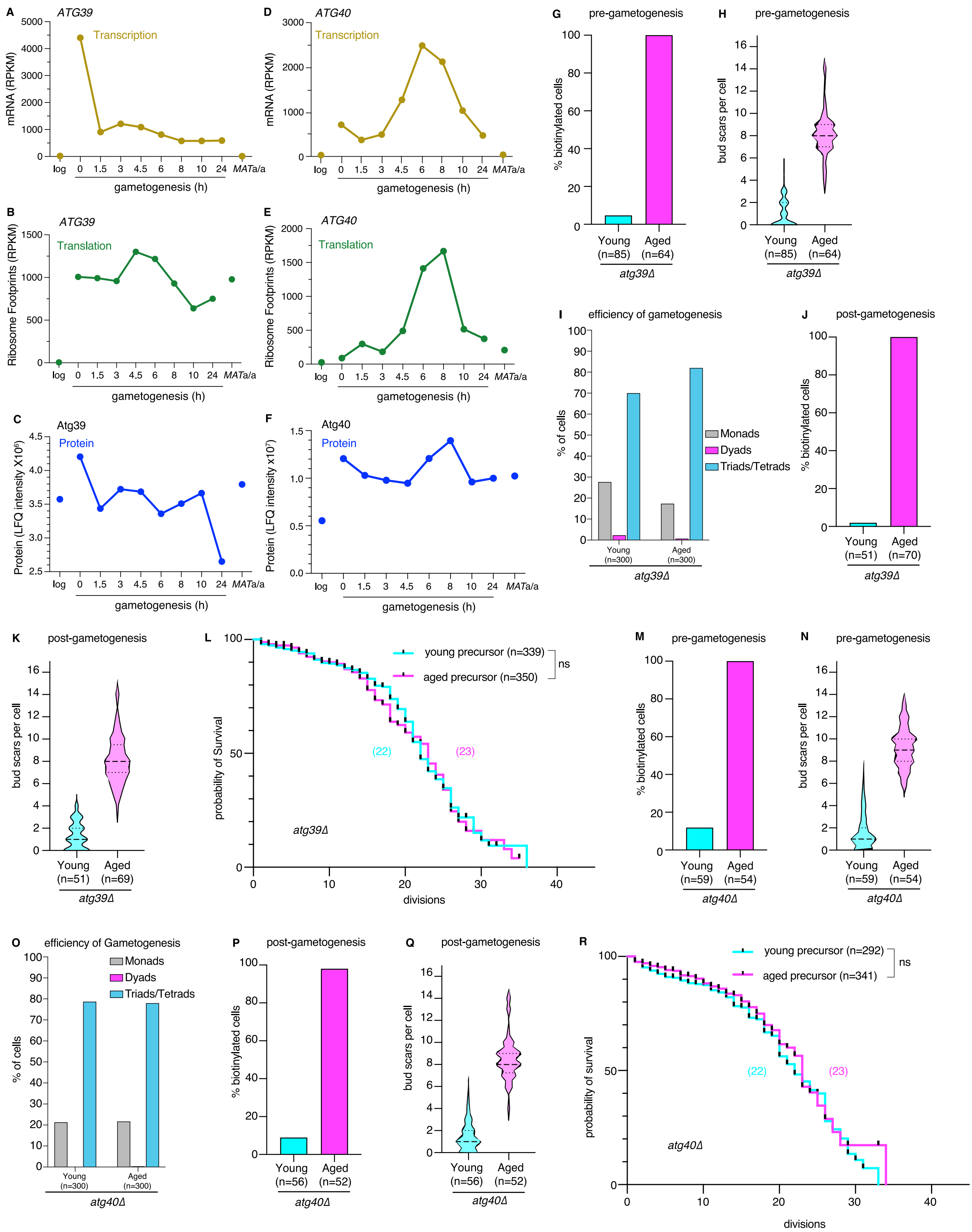

Supplementary Figure 7. Supporting data for *atg39Δ* and *atg40Δ* single mutants reset replicative lifespan during gametogenesis. **A-F** Expression of *ATG39* and *ATG40* either during log-phase vegetative growth in 2% glucose containing media (log), the indicated time in gametogenesis inducing media (h), or in control diploids that cannot undergo gametogenesis (*MAT***a**/**a**) plotted from previously generated meiotic datasets[1]. **A** *ATG39* transcript levels (RNA-seq). **B** *ATG39* translation levels (ribosome-protected footprints). **C** Atg39 protein levels (mass spectrometry). **D** *ATG40* transcript levels (RNA-seq). **E** *ATG40* translation levels (ribosome-protected footprints). **F** Atg40 protein levels (mass spectrometry). **G** Fraction of biotinylated cells in young (4.7%, 95% CI: 1.8–11.5%, n=85) and aged (all, n=64) *atg39Δ* cell

populations. **H** Quantification of replicative age by assessing number of bud scars in young (0.98 ± 1.2, mean ± SD, n=85) and aged (8.1 ± 1.8, mean ± SD, n=64) *atg39Δ* cell populations. **I** Fraction of tetrads in young (70.0%, 95% CI: 64.6–74.9%, n=300) and aged (82.0% (95% CI: 77.3–85.9%, n=300) post-gametogenic *atg39Δ* cell populations. **J** Fraction of biotinylated tetrads in young (2.0%, 95% CI: 0.3–10.3%, n=51) and aged (all, n=70) *atg39Δ* populations after completion of gametogenesis. **K** Quantification of replicative age by assessing number of bud scars in young (1.3± 1.2, mean ± SD, n=51) and aged (8.1± 2.1, mean ± SD, n=69) *atg39Δ* gamete populations. **L** Quantification of replicative lifespan of young (median lifespan=22, n=339, 188 censored subjects) and aged *atg39Δ* (median lifespan=23, n=350, 226 censored subjects, p=0.5881 compared to young) gametes. **M** Fraction of biotinylated cells in young (11.9%, 95% CI: 5.9–22.5%, n=59) and aged (all, n=54) *atg40Δ* cell populations. **N** Quantification of replicative age by assessing number of bud scars in young (1.6± 1.6, mean ± SD, n=59) and aged (9.3± 1.8, mean ± SD, n=54) *atg40Δ* cell populations. **O** Fraction of tetrads in young (78.7%, 95% CI: 73.7–82.9%, n=300) and aged (78.0%, 95% CI: 73.0–82.3%, n=300) post-gametogenic *atg39Δ* cell populations. **P** Fraction of biotinylated tetrads in young (8.9%, 95% CI: 3.9–19.3%, n=56) and aged (98.1%, 95% CI: 89.9–99.7%, n=52) *atg40Δ* populations after completion of gametogenesis. **Q** Quantification of replicative age by assessing number of bud scars in young (1.4± 1.4, mean ± SD, n=56), and aged (8.6± 1.9, mean ± SD, n=52) *atg40Δ* gamete populations. **R** Quantification of replicative lifespan of young (median lifespan=22, n=292, 176 censored subjects) and aged *atg40Δ* (median lifespan=23, n=341, 256 censored subjects, p=0.3077 compared to young) gametes.
Bar graphs show mean, confidence intervals were calculated using the Wilson score method. Solid lines in violin plots indicate the median, dotted lines indicate the 25th and 75th percentiles. Statistical significance for differences between replicative lifespans was calculated using the Log-rank (Mantel-Cox) test, tick marks on survival curves represent censored events. P values are indicated (ns, P>0.05, *P≤0.05, **P≤0.01, ***P≤0.001, ****P≤0.0001). Source data are provided as a Source Data file.

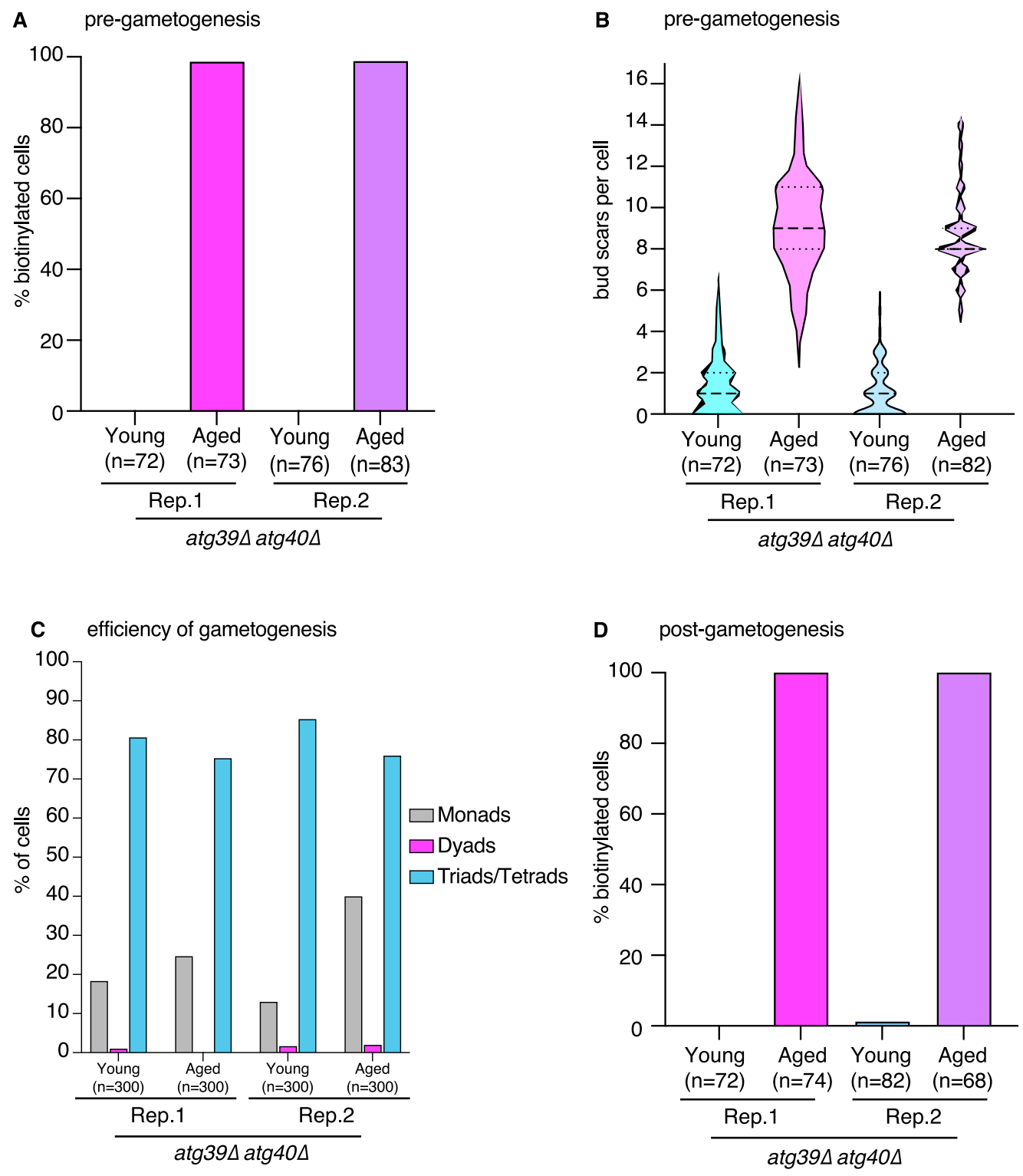

Supplementary Figure 8. Supporting data for *atg39Δ atg40Δ* double mutants fail to reset replicative lifespan during gametogenesis. **A** Fraction of biotinylated cells in *atg39Δ atg40Δ* cell populations: Replicate 1 (Rep. 1) young (none, n=72) and aged (98.6%, 95% CI: 92.6–99.8%, n=73); Replicate 2 (Rep. 2) young (none, n=76) and aged (98.8%, 95% CI: 93.5–99.8%, n=83). **B** Quantification of replicative age by assessing number of bud scars in *atg39Δ atg40Δ* cell populations: Rep. 1 young (1.4± 1.4, mean ± SD, n=72) and aged (9.1± 2.3, mean ± SD, n=73); Rep. 2 young (1 ± 1.2, mean ± SD, n=76) and aged (8.7± 1.9, mean ± SD, n=82). **C** Fraction of tetrads in post-gametogenic *atg39Δ atg40Δ* cell populations: Rep.1 young (80.7%, 95% CI: 75.8–84.7%, n=300) and aged (75.3%, 95% CI: 70.2–79.9%, n=300); Rep. 2 young (85.3%, 95% CI: 80.9–88.9%, n=300) and aged (76.0%, 95% CI: 70.9–80.5%, n=300). **D** Fraction of biotinylated tetrads in *atg39Δ atg40Δ* populations after completion of gametogenesis: Rep.1 young (none, n=72) and aged (all, n=74); Rep. 2 young (1.2%, 95% CI: 0.2–6.6%, n=82) and aged (all, n=68).
Bar graphs show mean, confidence intervals were calculated using the Wilson score method. Solid lines in violin plots indicate the median, dotted lines indicate the 25th and 75th percentiles. Source data are provided as a Source Data file.

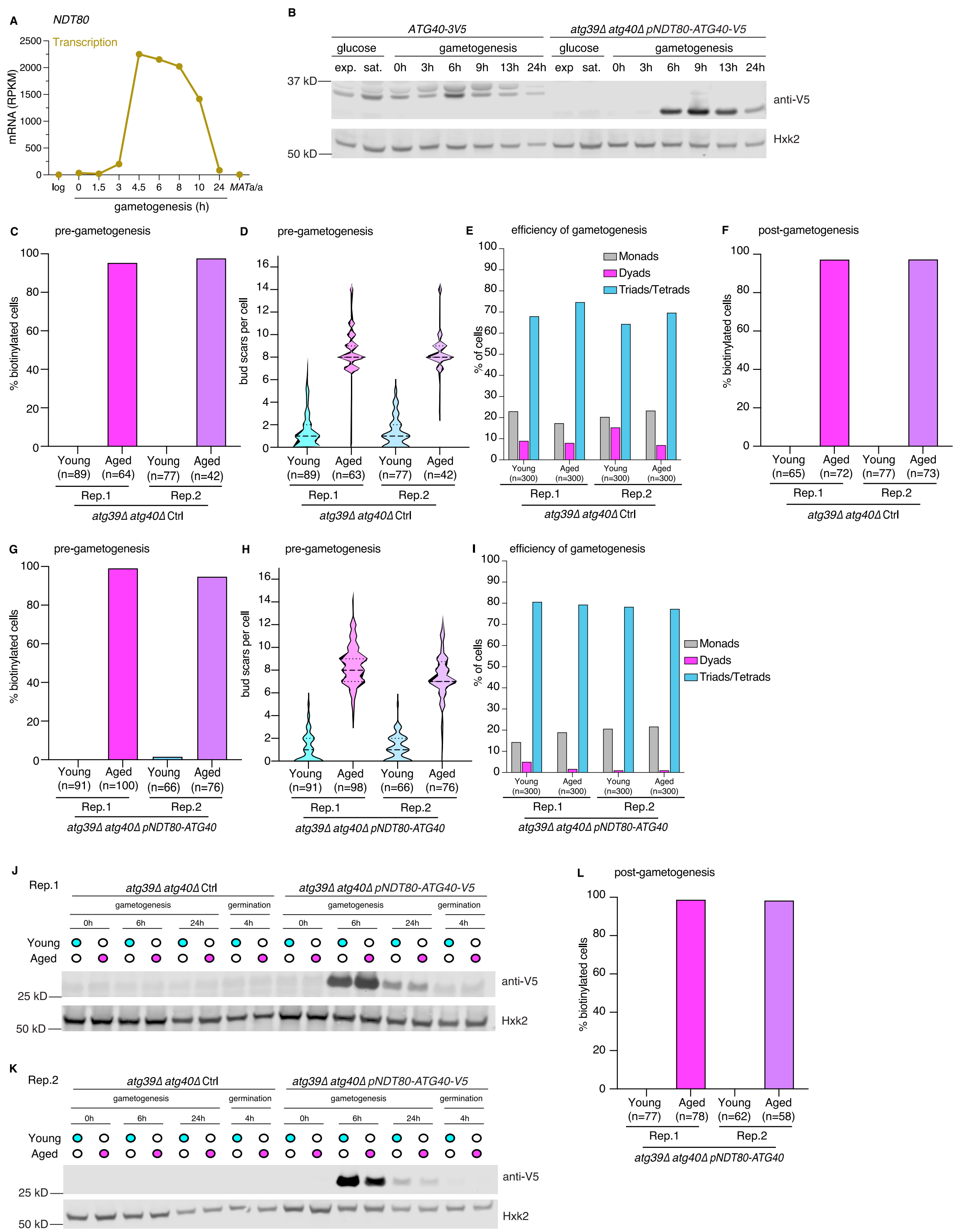

Supplementary Figure 9. Supporting data for meiosis specific expression of *ATG40* is sufficient to rescue reset of replicative lifespan in cells that lack *ATG40 and ATG39.* **A** *NDT80* transcript levels (RNA-seq) either during log-phase vegetative growth in 2% glucose containing media (log), the indicated time in gametogenesis inducing media (h), or in control diploids that cannot undergo gametogenesis (*MAT***a**/**a**) plotted from previously generated meiotic datasets[1]. **B** Immunoblot comparing endogenous Atg40-3V5 to pNDT80-Atg40-V5 rescue construct protein levels (anti-V5) and loading control (Hxk2) during exponential and saturated growth in media containing 2% glucose (glucose) and at different timepoints upon transfer to gametogenesis-inducing media (gametogenesis). **C** Fraction of biotinylated cells in *atg39Δ atg40Δ* control cell populations: Replicate 1 (Rep. 1) young (none, n=89) and aged

(95.3%, 95% CI: 87.1–98.4%, n=64); Replicate 2 (Rep. 2) young (none, n=77) and aged (97.6%, 95% CI: 87.7–99.6%, n=44). **D** Quantification of replicative age by assessing number of bud scars in *atg39Δ atg40Δ* control cell populations: Rep. 1 young (1.2± 1.4, mean ± SD, n=89) and aged (8.5± 1.9, mean ± SD, n=63); Rep. 2 young (1.3 ± 1.3, mean ± SD, n=77) and aged (8.5± 1.9, mean ± SD, n=42). **E** Fraction of tetrads in post-gametogenic *atg39Δ atg40Δ* control cell populations: Rep.1 young (68.0%, 95% CI: 62.5–73.0%, n=300) and aged (74.7%, 95% CI: 69.5–79.3%, n=300); Rep. 2 young (64.3%, 95% CI: 58.8–69.5%, n=300) and aged (69.7%, 95% CI: 64.2–74.6%, n=300). **F** Fraction of biotinylated tetrads in *atg39Δ atg40Δ* control populations after completion of gametogenesis: Rep.1 young (none, n=65) and aged (97.2%, 95% CI: 90.4–99.2%, n=72); Rep. 2 young (none, n=77) and aged (97.3%, 95% CI: 90.5–99.2%, n=73). **G** Fraction of biotinylated cells in *atg39Δ atg40Δ pNDT80-ATG40* cell populations: Replicate 1 (Rep. 1) young (none, n=91) and aged (99.0%, 95% CI: 94.6–99.8%, n=100); Replicate 2 (Rep. 2) young (1.5%, 95% CI: 0.3–8.1%, n=66) and aged (94.7%, 95% CI: 87.2–97.9%, n=76). **H** Quantification of replicative age by assessing number of bud scars in *atg39Δ atg40Δ pNDT80-ATG40* cell populations: Rep. 1 young (1.0± 1.3, mean ± SD, n=91) and aged (8.4± 1.3, mean ± SD, n=98); Rep. 2 young (1.1 ± 1.1, mean ± SD, n=66) and aged (7.3± 2.1, mean ± SD, n=76). **I** Fraction of tetrads in post-gametogenic *atg39Δ atg40Δ pNDT80-ATG40* cell populations: Rep.1 young (80.7%, 95% CI: 75.8–84.7%, n=300) and aged (79.3%, 95% CI: 74.4–83.5%, n=300); Rep. 2 young (78.3%, 95% CI: 73.3–82.6%, n=300) and aged (77.3%, 95% CI: 72.3–81.7%, n=300). **J&K** Immunoblot of young and aged control and pNDT80-Atg40-V5 samples to quantify protein levels (anti-V5) and loading control (Hxk2) at different timepoints upon transfer to gametogenesis-inducing media (gametogenesis) and after re-growth in media containing 2% glucose (germination). (**J**) Samples collected from Rep.1. **(K**) Samples collected from Rep.2. **L** Fraction of biotinylated tetrads in *atg39Δ atg40Δ pNDT80-ATG40* cell populations after completion of gametogenesis: Rep.1 young (none, n=77) and aged (98.7%, 95% CI: 93.1–99.8%, n=78); Rep. 2 young (none, n=62) and aged (98.3%, 95% CI: 90.9–99.7%, n=58).

Bar graphs show mean, confidence intervals were calculated using the Wilson score method. Solid lines in violin plots indicate the median, dotted lines indicate the 25th and 75th percentiles. Source data are provided as a Source Data file.

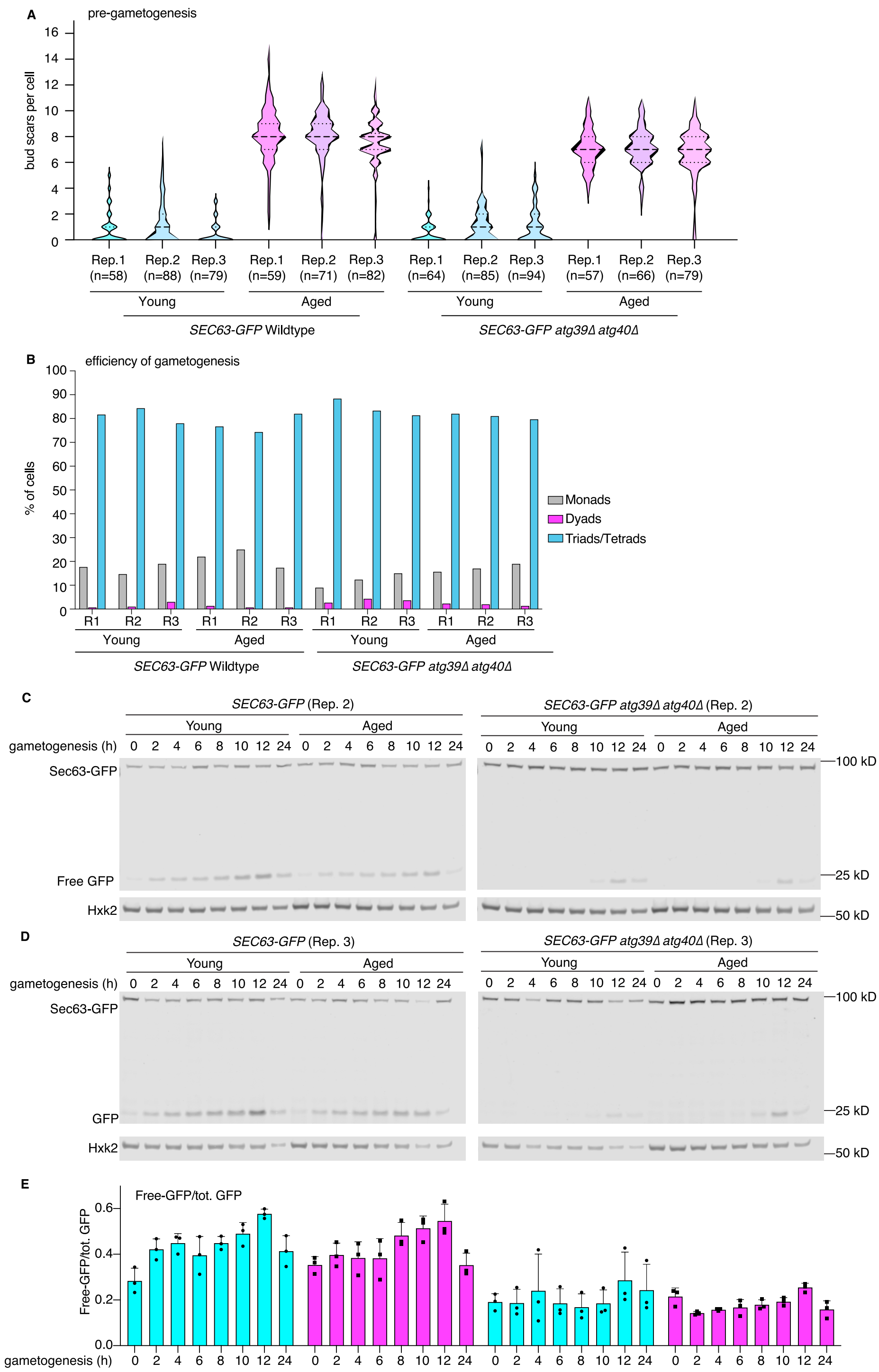

Supplementary Figure 10. Supporting data for accumulation of ER components during gametogenesis in cells that lack *ATG40 and ATG39.* **A** Quantification of replicative age by assessing number of bud scars in *SEC63-GFP* and *SEC63-GFP atg39Δ atg40Δ* cell populations. *SEC63-GFP* cell populations: Rep. 1 young (0.9± 1.3, mean ± SD, n=58) and aged (8.2± 2, mean ± SD, n=59); Rep. 2 young (1.3 ± 1.7, mean ± SD, n=88) and aged (8± 2, mean ± SD, n=71); Rep. 3 young (0.51 ± 0.89, mean ± SD, n=79) and aged (7.5± 2, mean ± SD, n=82). *SEC63-GFP atg39Δ atg40Δ* cell populations: Rep. 1 young (0.55± 0.85, mean ± SD, n=64) and aged (7± 1.4, mean ± SD, n=57); Rep. 2 young (1.1 ± 1.4, mean ± SD, n=85) and aged (7.2± 1.4, mean ± SD, n=66); Rep. 2 young (1.1 ± 1.4, mean ± SD, n=94) and aged (6.7± 1.9, mean ± SD, n=79). **B** Fraction of tetrads in post gametogenic *SEC63-*

GFP and *SEC63-GFP atg39Δ atg40Δ* cell populations. *SEC63-GFP* cell populations: Rep.1 young (81.7%, 95% CI: 76.9–85.6%, n=300) and aged (76.7%, 95% CI: 71.6–81.1%, n=300); Rep. 2 young (84.3%, 95% CI: 79.83–88.0%, n=300) and aged (74.3%, 95% CI: 69.1–78.9%, n=300); Rep. 3 young (78.0%, 95% CI: 73.0–82.3%, n=300) and aged (82.0%, 95% CI: 77.3–85.9%, n=300). *SEC63-GFP atg39Δ atg40Δ* cell populations: Rep.1 young (88.3%, 95% CI: 84.2–91.5%, n=300) and aged (82.0%, 95% CI: 77.3–85.9%, n=300); Rep. 2 young (83.3%, 95% CI: 78.7–87.1%, n=300) and aged (81.0%, 95% CI: 76.2–85.0%, n=300); Rep. 3 young (81.3%, 95% CI: 76.5–85.3%, n=300) and aged (79.7%, 95% CI: 74.8–83.8%, n=300). Bar graphs show mean, confidence intervals were calculated using the Wilson score method. **C** Immunoblot of protein samples form young and aged *SEC63-GFP* (left blot) and *atg39Δ atg40Δ SEC63-GFP* cells (right blot) to quantify protein levels (anti-GFP) and loading control (Hxk2) at different timepoints upon transfer to gametogenesis-inducing media (gametogenesis (h)), second replica used for quantification of Sec63-GFP levels normalize to loading control (Hxk2) (Fig. 6E). **D** Immunoblot of protein samples form young and aged *SEC63-GFP GFP* (left blot) and *atg39Δ atg40Δ SEC63-GFP* cells (right blot) cells to quantify protein levels (anti-GFP) and loading control (Hxk2) at different timepoints upon transfer to gametogenesis-inducing media (gametogenesis (h)), third replica used for quantification of Sec63-GFP levels normalize to loading control (Hxk2) (Fig. 6E). **E** Quantification of Free-GFP originating from degraded Sec63-GFP as readout for ER-phagy. Bar graphs in cyan & magenta indicate quantification of Free-GFP levels normalize to total GFP (Sec63-GFP+Free-GFP). Bar graphs depict mean and error bars show ± SD from 3 independent replicas.
Solid lines in violin plots indicate the median, dotted lines indicate the 25th and 75th percentiles. Source data are provided as a Source Data file.

Supplementary Table 1. Lifespan comparisons

Genotype	Strain background	Method	Mean Replicative lifespan	% Change relative to Wildtype	Reference
Wildtype	SK1	Microfluidics	21	0	This study
sir2Δ	SK1	Microfluidics	11	-47.6	This study
fob1Δ	SK1	Microfluidics	28	+33.3	This study
Wildtype	SK1	Microdissections	19.55	0	[2]
sir2Δ	SK1	Microdissections	8.87	-54.6	[2]
fob1Δ	SK1	Microdissections	23.57	+20.6	[2]
Wildtype	W303R	Microdissections	21.4	0	[3]
sir2Δ	W303R	Microdissections	11.6	-45.8	[3]
Wildtype	W303R	Microdissections	19.5	0	[4]
fob1Δ	W303R	Microdissections	24.4	+25.12	[4]

Supplementary Table 2. Strain Table

Strain number yÜB[1]	MAT	Genotype[2,3]	Source[4]
SK1	Strain background	*ho::LYS2, lys2, leu2::hisG, ura3, his3::hisG, trp1::hisG*	
13	*MAT**a***	WT	
14	*MAT**α***	WT	
15	*MAT**a/α*** (diploid)	WT	
29559	diploid	*trp1::pPMA2-PMA2-eGFP::TRP1, HTB1-mCherry-HISMX6* (heterozygote with *his3::hisG*)	this study
42275	diploid	*trp1::pPMA2-PMA2-eGFP::TRP1, ste5::NatR-pTet07.1-STE5, ura3::pRNR2-TetR-Tup1, pTetO7.1-TetR::URA3, flo8Δ, Amn1^{D368V}*	this study
42274	diploid	*trp1::pPMA2-PMA2-eGFP::TRP1, ste5::NatR-pTet07.1-STE5, ura3::pRNR2-TetR-Tup1, pTetO7.1-TetR::URA3, flo8Δ, Amn1^{D368V}*	this study
43791	diploid	*sir2Δ::KanMX* (heterozygote with *sir2Δ::HISMX6), trp1::pPMA2-PMA2-eGFP::TRP1, ste5::NatR-pTet07.1-STE5, ura3::pRNR2-TetR-Tup1, pTetO7.1-TetR::URA3, flo8Δ, Amn1^{D368V}*	this study
43766	diploid	*fob1Δ::KanMX, trp1::pPMA2-PMA2-eGFP::TRP1, ste5::NatR-pTet07.1-STE5, ura3::pRNR2-TetR-Tup1, pTetO7.1-TetR::URA3, flo8Δ, Amn1^{D368V}*	this study
45418	diploid	*sir2Δ::KanMX, sir2Δ::HISMX6, leu2::pRIM4-SIR2-V5::LEU2* (heterozygote with *leu2::hisG*), *trp1::pPMA2-PMA2-eGFP::TRP1, ste5::NatR-pTet07.1-STE5, ura3::pRNR2-TetR-Tup1, pTetO7.1-TetR::URA3, flo8Δ, Amn1^{D368V}*	this study
48561	diploid	*atg11Δ::KanMX, trp1::pPMA2-PMA2-eGFP::TRP1, ste5::NatR-pTet07.1-STE5, ura3::pRNR2-TetR-Tup1, pTetO7.1-TetR::URA3, flo8Δ, Amn1^{D368V}*	this study
45501	diploid	*atg32Δ::KanMX, trp1::pPMA2-PMA2-eGFP::TRP1, ste5::NatR-pTet07.1-STE5, ura3::pRNR2-TetR-Tup1, pTetO7.1-TetR::URA3, flo8Δ, Amn1^{D368V}*	this study
48175	diploid	*atg33Δ::KanMX, trp1::pPMA2-PMA2-eGFP::TRP1, ste5::NatR-pTet07.1-STE5, ura3::pRNR2-TetR-Tup1, pTetO7.1-TetR::URA3, flo8Δ, Amn1^{D368V}*	this study
48216	diploid	*atg32Δ::KanMX, atg33Δ::KanMX, trp1::pPMA2-PMA2-eGFP::TRP1, ste5::NatR-pTet07.1-STE5, ura3::pRNR2-TetR-Tup1, pTetO7.1-TetR::URA3, flo8Δ, Amn1^{D368V}*	this study
44411	diploid	*atg39Δ::NatMX, trp1::pPMA2-PMA2-eGFP::TRP1, ste5::NatR-pTet07.1-STE5, ura3::pRNR2-TetR-Tup1, pTetO7.1-TetR::URA3, flo8Δ, Amn1^{D368V}*	this study

42753	diploid	*atg40Δ::KanMX, trp1::pPMA2-PMA2-eGFP::TRP1, ste5::NatR-pTet07.1-STE5, ura3::pRNR2-TetR-Tup1, pTetO7.1-TetR::URA3, flo8Δ, Amn1*^{D368V}	this study
45176	diploid	*atg39Δ::NatMX, atg40Δ::KanMX, trp1::pPMA2-PMA2-eGFP::TRP1, ste5::NatR-pTet07.1-STE5, ura3::pRNR2-TetR-Tup1, pTetO7.1-TetR::URA3, flo8Δ, Amn1*^{D368V}	this study
47417	diploid	*leu2::LEU2, atg39Δ::NatMX, atg40Δ::KanMX, trp1::pPMA2-PMA2-eGFP::TRP1, ste5::NatR-pTet07.1-STE5, ura3::pRNR2-TetR-Tup1, pTetO7.1-TetR::URA3, flo8Δ, Amn1*^{D368V}	this study
48312	diploid	*leu2::pNDT80-ATG40-V5::LEU2, atg39Δ::NatMX, atg40Δ::KanMX, trp1::pPMA2-PMA2-eGFP::TRP1, ste5::NatR-pTet07.1-STE5, ura3::pRNR2-TetR-Tup1, pTetO7.1-TetR::URA3, flo8Δ, Amn1*^{D368V}	this study
14554	diploid	*atg40::ATG40-3V5-KanMX*	[5]
49061	diploid	*atg39Δ::NatMX, atg40Δ::KanMX, HTB1-mCherry-HISMX6, sec63::SEC63-EGFP::KanMX, flo8Δ, Amn1*^{D368V}	this study
49061	dipoid	*HTB1-mCherry-HISMX6, sec63::SEC63-EGFP::KanMX, flo8Δ, Amn1*^{D368V}	this study

[1]Strains are listed according to Figure order.
[2]Alleles are separated by commas.
[3]All indicated alleles in diploids are homozygous, unless otherwise specified.
[4]Previously generated alleles are further described elsewhere (see Methods).

Supplementary Table 3. Plasmids

Plasmid number (pÜB)	Construct[1]	Backbone	Bacterial selection	Source / Reference
1	*KanMX6*	pFA6a	Ampicilin	[6]
153	*NatMX4*	pFA6a	Ampicilin	[6]
182	*linker-eGFP_KanMX6*	pFA6a		Adapted from[6]
2693	*NatMX4_ pTetO7.1*	pFA6a	Ampicilin	Adapted from[7]
2255	*kanMX6-ins1*	pFA6a	Ampicilin	[8]
2309	*his3MX6-ins3*	pFA6a	Ampicilin	[8]
1905	*pPMA2-PMA2-eGPF_LEU2*	pNH604	Ampicilin	This study
2991	*pRIM4-SIR2-V5_LEU2*	pLC605	Ampicilin	This study
3076	*pNDT80-ATG40-V5_LEU2*	pÜB2991	Ampicilin	This study
2371	*pRNR2-TetR-TUP1_pTetO7.1-TetR_URA3*	pSV606	Ampicilin	Adapted from[7]

[1]Underscores indicate separation between plasmid elements (e.g. construct and selection cassette).

Supplementary Table 4. Primers

Allele	Forward primer	Reverse primer	Template
pTetO-STE5	CTAAAAAAGGAAGATACAGGATACAGCGGAAACAACTTTTAAcgtacgctgcaggtcgac	TGTGAAAAGGGGAAACTATATTGTCTGTAGGAGTTTCCATcatcgatgaattctctgtcg	pÜB2693
sir2Δ	CATTCAAACCATTTTTCCCTCATCGGCACATTAAAGCTGGcggatccccgggttaattaa	TATTAATTTGGCACTTTTAAATTATTAAATTGCCTTCTACgaattcgagctcgtttaaac	pÜB2255 & pÜB2309
fob1Δ	TTAACGATTGTGTGAGTGTGAATTTGTGCTGAGGATAACAcggatccccgggttaattaa	TGGAGATCAAACAAGAGACTTTGGCACATCAATGGAATTGcggatccccgggttaattaa	pÜB1
atg11Δ	GTTGTTCGGAAAGTACTTCTTTTATTTTCTTTTATGCATCcggatccccgggttaattaa	AATTAAAATCTTGTCATTTGTGACAAACGTTTAGCACTGTgaattcgagctcgtttaaac	pÜB2255
atg33Δ	TACTACTTCACGATATACAGAAGTAAAAACATATAAAAAGcggatccccgggttaattaa	CTACAAATTCTTGTAGAGATAAATACGTGAACGAGAAACAgaattcgagctcgtttaaac	pÜB2255
SEC63-EGFP	CGATACGGATACAGAAGCTGAAGATGATGAATCACCAGAAGGTGACGGTGCTGGTTTA	TCTAAGAGCTAAAATGAAAAACTATACTAATCACTTATATTCGATGAATTCGAGCTCG	pÜB182

[1] Lowercase letters indicate common primer adaptor sequences[6,8], uppercase letters indicate primer tail introducing homology to target gene locus.
[2] pÜB plasmids can be found in Table S3.

Supplementary Figures Source Data

Supplementary Figure 4I - Uncropped Scans

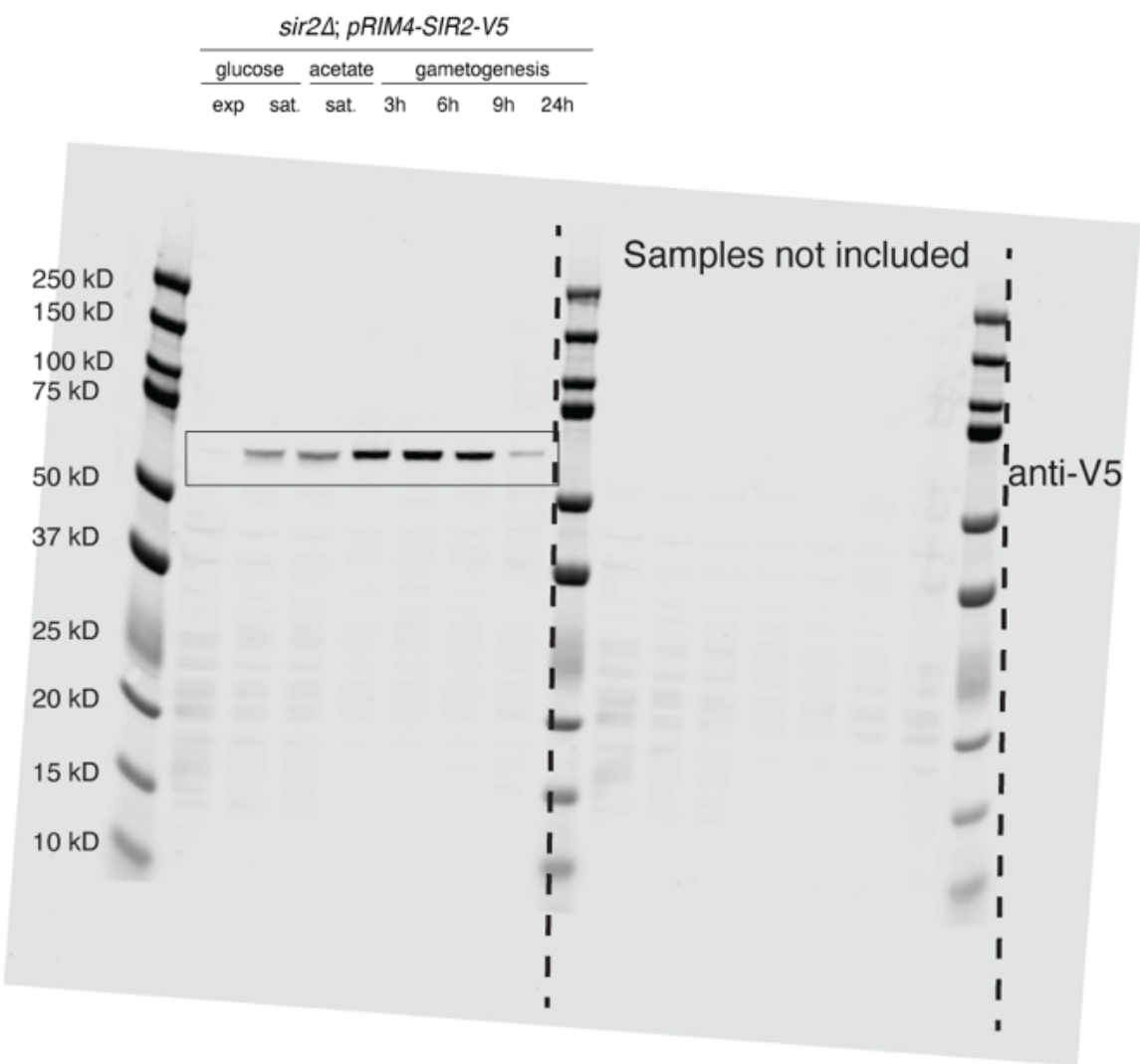

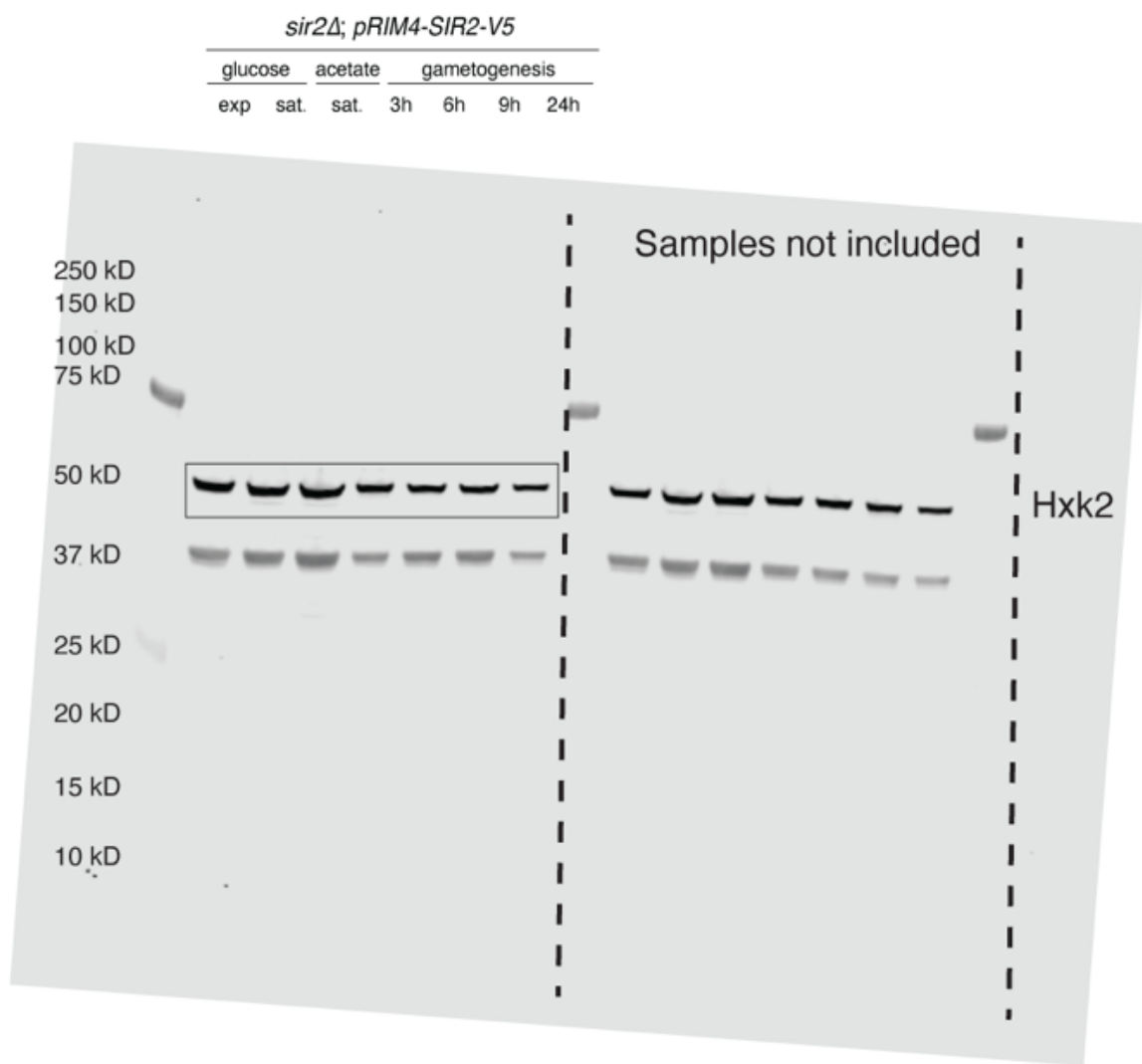

Supplementary Figure 9B, 9J&K - Uncropped Scans

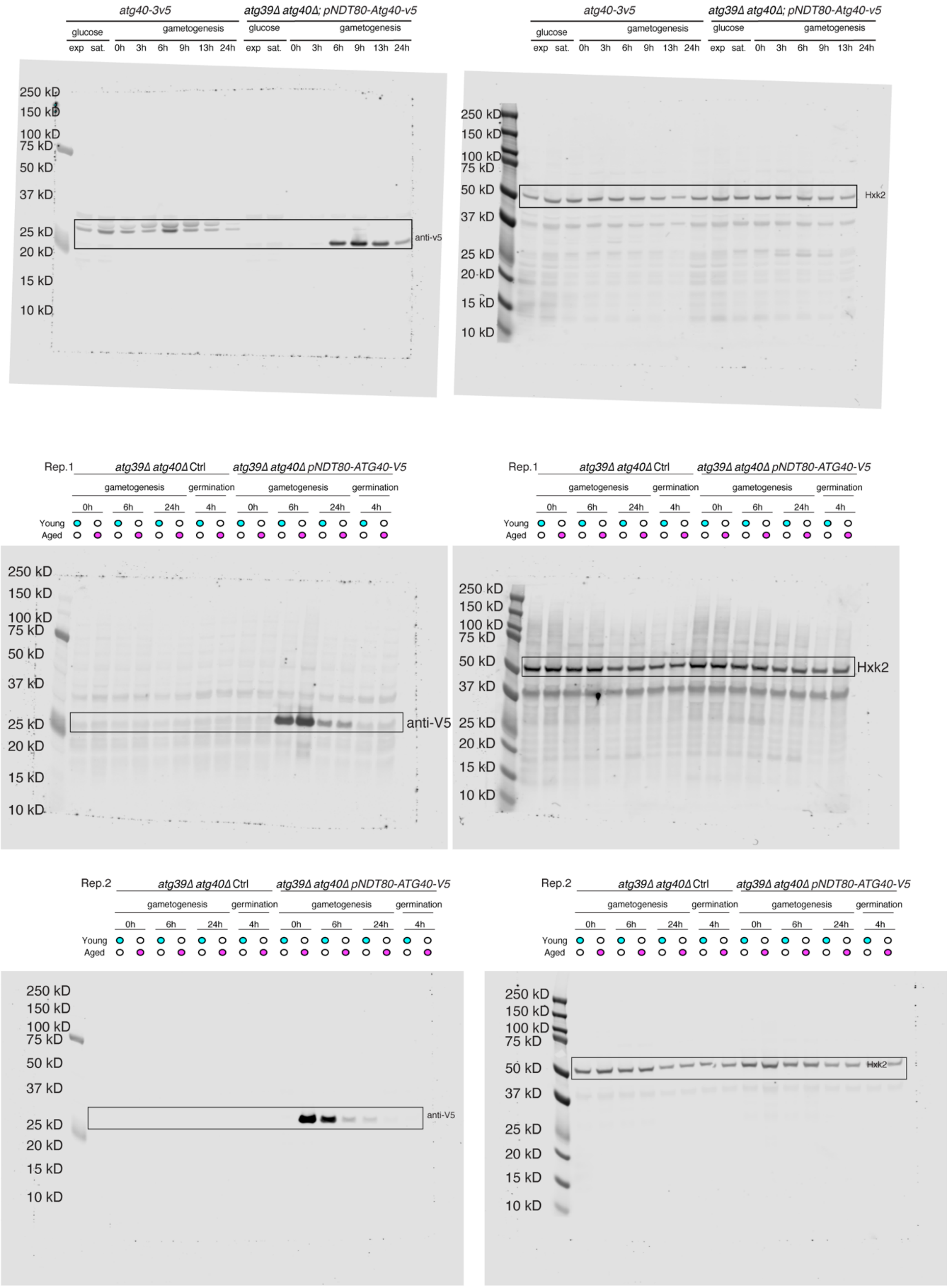

Supplementary Figure 10D&E - Uncropped Scans

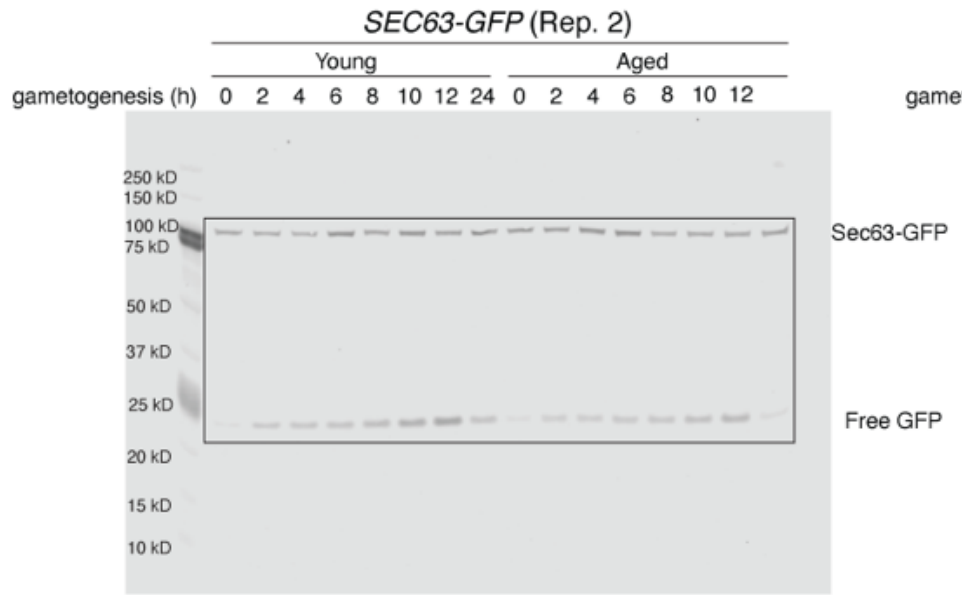

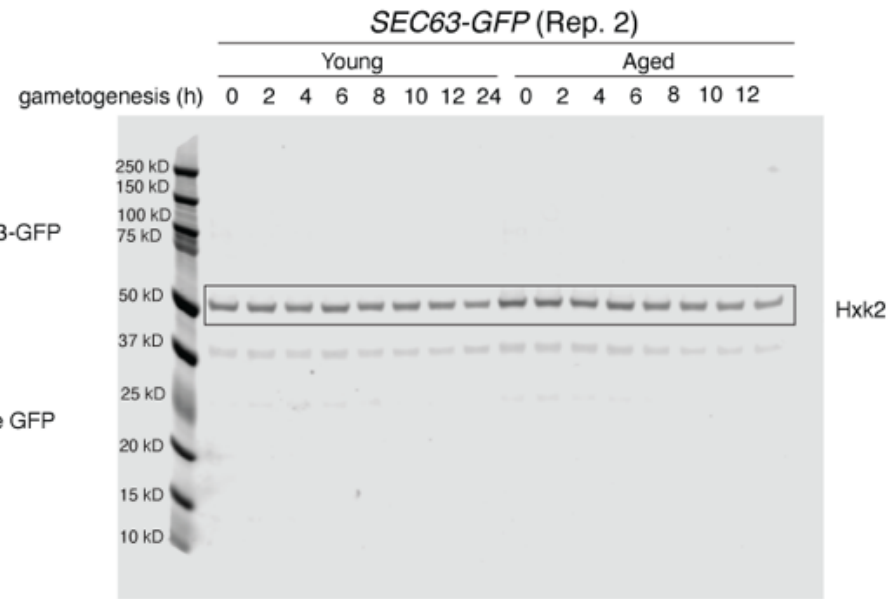

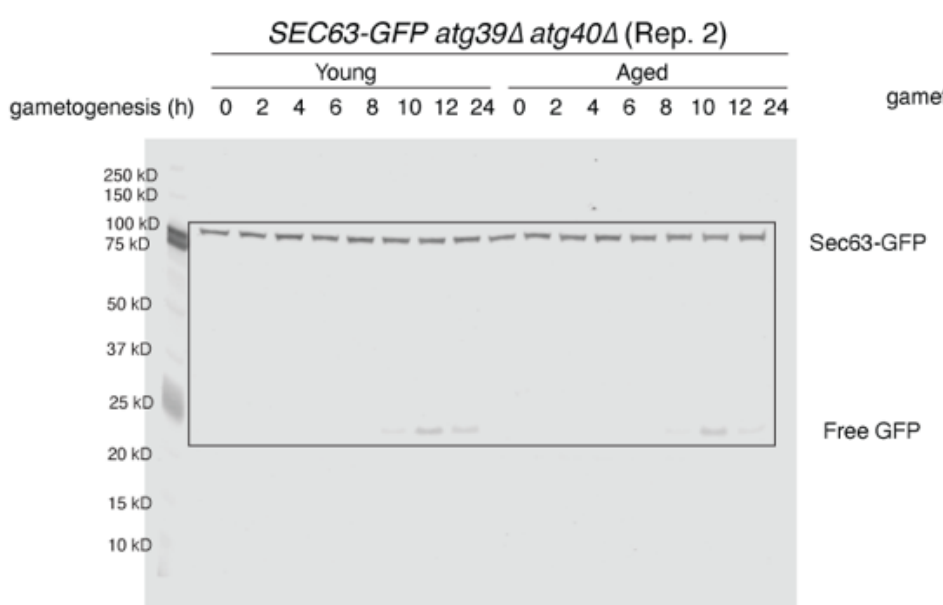

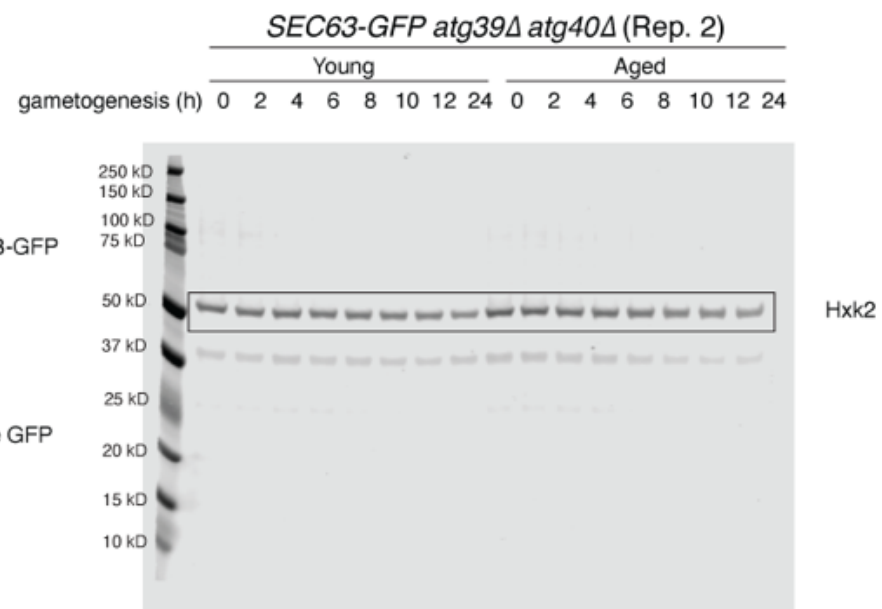

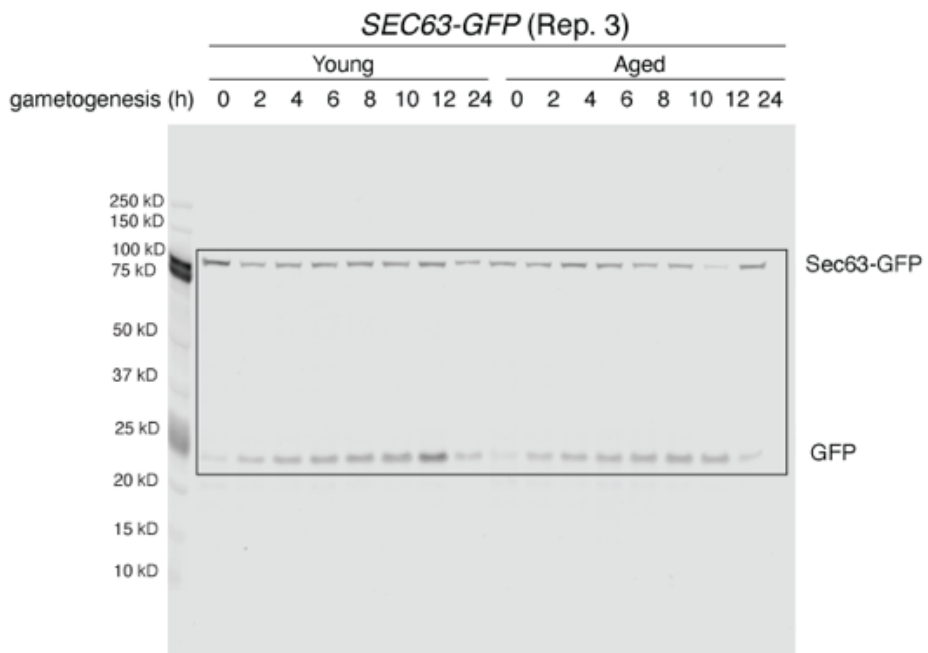

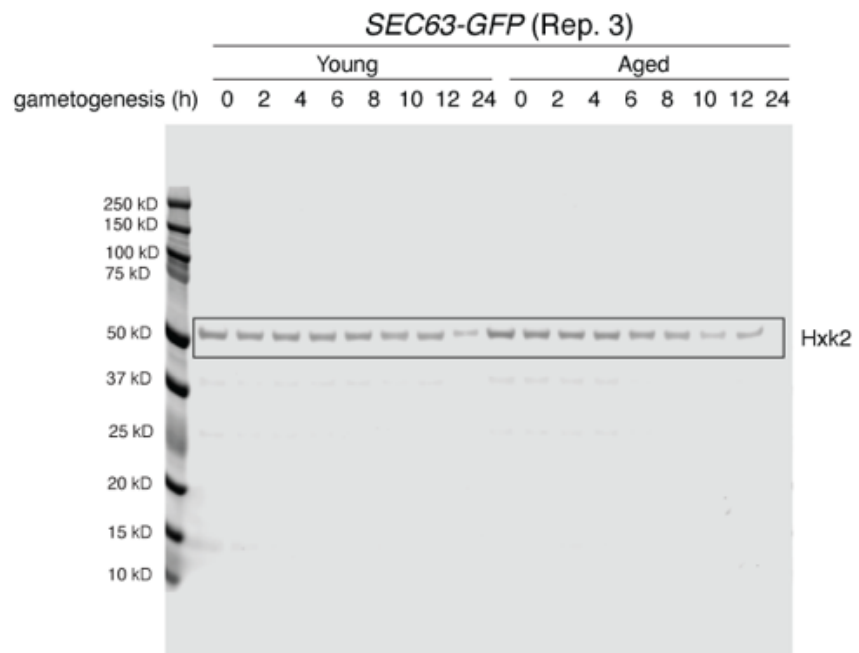

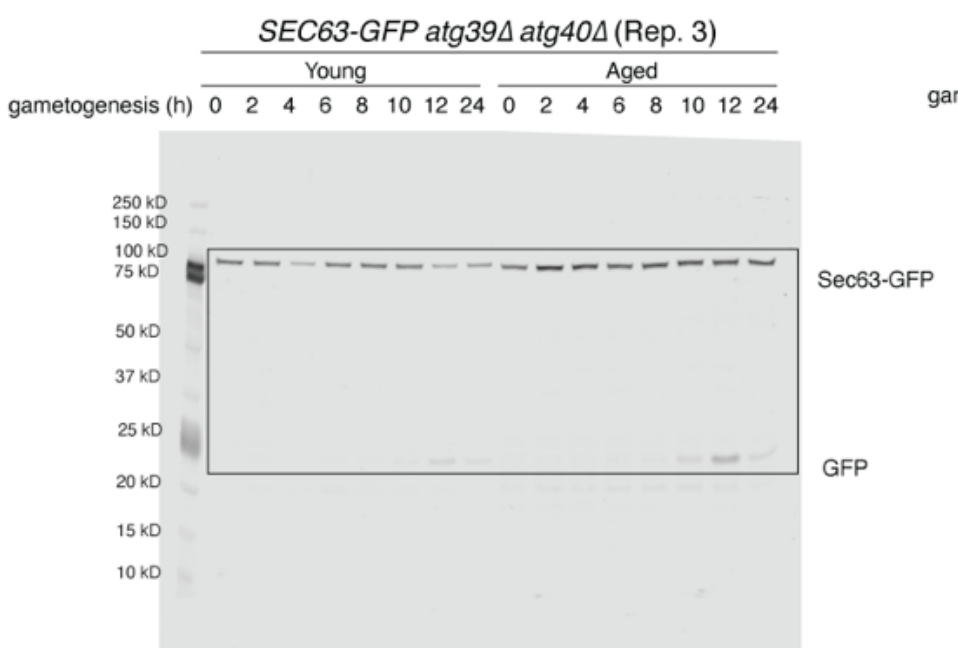

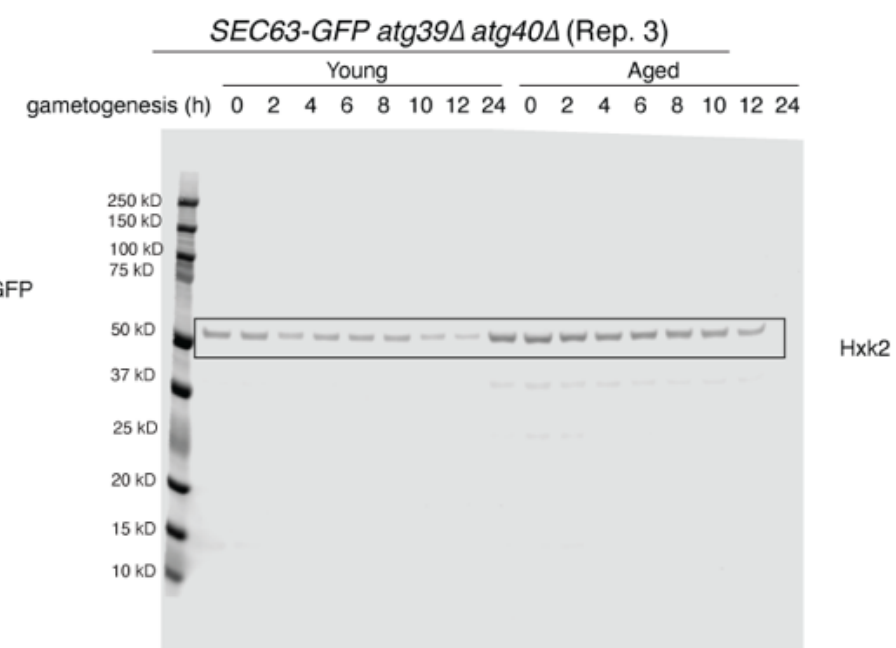

Supplementary References

1. Cheng, Z. *et al.* Pervasive, Coordinated Protein-Level Changes Driven by Transcript Isoform Switching during Meiosis. *Cell* **172**, 910-923.e16 (2018).
2. Boselli, M., Rock, J., Ünal, E., Levine, S. S. & Amon, A. Effects of Age on Meiosis in Budding Yeast. *Developmental Cell* **16**, 844–855 (2009).
3. Kaeberlein, M., McVey, M. & Guarente, L. The SIR2/3/4 complex and SIR2 alone promote longevity in Saccharomyces cerevisiae by two different mechanisms. *Genes Dev* **13**, 2570–2580 (1999).
4. Defossez, P.-A. *et al.* Elimination of Replication Block Protein Fob1 Extends the Life Span of Yeast Mother Cells. *Molecular Cell* **3**, 447–455 (1999).
5. Otto, G. M., Cheunkarndee, T., Leslie, J. M. & Brar, G. A. Programmed cortical ER collapse drives selective ER degradation and inheritance in yeast meiosis. *Journal of Cell Biology* **220**, e202108105 (2021).
6. Longtine, M. S. *et al.* Additional modules for versatile and economical PCR-based gene deletion and modification in Saccharomyces cerevisiae. *Yeast* **14**, 953–961 (1998).
7. Azizoglu, A., Brent, R. & Rudolf, F. A precisely adjustable, variation-suppressed eukaryotic transcriptional controller to enable genetic discovery. *eLife* **10**, e69549 (2021).
8. Powers, E. N. *et al.* Bidirectional promoter activity from expression cassettes can drive off-target repression of neighboring gene translation. *eLife* **11**, e81086 (2022).
