# Supplementary material for: A role for selective ER autophagy in gametogenic rejuvenation revealed by high-throughput lifespan profiling
Source: Nat Commun. 2026 Sep 29;17:11215. doi: 10.1038/s41467-026-77993-0 (PMC13720134; doi:10.1038/s41467-026-77993-0)
Supplement: Supplementary file 3 [file 41467_2026_77993_MOESM3_ESM.pdf]

nature portfolio

Peer Review File

A role for selective ER autophagy in gametogenic rejuvenation revealed by high-throughput lifespan profiling

Corresponding Author: Professor Gloria Brar

This file contains all reviewer reports in order by version, followed by all author rebuttals in order by version.

Version 0:

Reviewer comments:

Reviewer #1

(Remarks to the Author)
This very interesting paper by Spiri et al. primarily describes a new technique for measuring the reset of replicative lifespan in budding yeast after meiosis: microfluidic trapping of individual gametes for observation by microscopy. Although microfluidics had been previously used for measuring replicative lifespan in yeast during vegetative growth, it had not been applied to gametes after meiosis. The application of microfluidics allowed the authors much higher throughput than had been achieved with previous methods that required micromanipulation of the gametes. Using microfluidics, they were able to verify the previously reported reset of replicative lifespan by meiosis with a larger sample size. In addition, they were able for the first time to identify some of the factors required for this reset: the ER-phagy receptors Atg39 and Atg40, along with the selective autophagy adaptor Atg11. This is an exciting finding that also demonstrates the power of this approach.

This appears to be a very rigorous study, whose conclusions are well-supported by clear and conclusive data. The cell-sorting techniques used to separate young from old gametes are thoroughly validated and very efficient, and quality-control data about the cell populations (e.g. efficiency of gametogenesis and assessment of average population age via bud-scar counting) are presented for every experiment in the supplemental data. The methods are thorough and well-described, including the key genetic and experimental manipulations that were necessary to apply microfluidics techniques to the study of meiosis.

There are only a few things I would like the authors to address before I am ready to recommend this for publication:

1) In figure 6F, the accumulation of the Sec63-GFP is very subtle to see in the fluorescent microcopy images, and indeed the naked eye is not very good at judging the brightness of fluorescent images. To better support the conclusion that “Sec63-GFP accumulates during gametogenesis in young and aged cells lacking atg39Δ atg40Δ”, please quantitate the average Sec63-GFP signal intensity in these strains and include that data as a graph in figure 6.

2) Also in figure 6, but in panel E: The authors have very nicely included both the Sec63-GFP and the free GFP signal on their blot. As they are presumably aware, the level of free GFP is a readout of ER-phagy. There appears to be less free GFP in the atg39Δ/atg40Δ mutant, consistent with the expected defect in ER-phagy. This is a nice control supporting their interpretation of these mutants, but does not seem to be mentioned. I would recommend adding a sentence or two to the results and to the figure legend making note of this. It would also be nice to see the quantification of free GFP/Hxk2 in their blots to further support this – this would ideally go in Figure 6 if space can be found, or else in a supplemental figure.

3) Although the methods were generally quite thorough, one thing I could not find was what dye was used for staining the bud scars, or a cited reference for this procedure. Please include this in the methods (perhaps after line 576), and in the legend for figure 2D (line 715)

4) Here are a few typos or presentation issues that I noticed that should be fixed:
a. In the supplemental data, the yellow line graphs with white background are very hard to read (e.g. Figure S1A and S4E, and others). Please change the yellow to a darker color, such as orange or brown, for more contrast in every place where this occurs.
b. Similarly, when either yellow or especially cyan are used in the microscopy images, the colored label on the gray background (e.g. “Biotin” and “Pma2” in Figure 2D) is difficult to read. The color of the actual microscopy images doesn’t

need to be changed, just the color of the label itself (or perhaps outline it in black of something of that sort to make it more visible).
c. There is a typo in figure S6L: the graph title says “Atg32”, but the figure legend indicates this is for Atg33.
d. Typo in line 392: The reference should be to Fig. S9G-K, not S8G-K.
e. Typo in line 836 – should be “from,” not “form”
f. In line 282, it would be better to say “vacuolar degradation”, since yeast have vacuoles instead of lysosomes. (Figure 5D is clear in this regard, so just the text needs to be updated.)
g. In line 322, no references are cited regarding Atg33 and its role in mitophagy. Please cite an appropriate paper here.

Reviewer #2

(Remarks to the Author)
This manuscript is well written. The experimental procedures are well controlled and documented, and the data interpretation is convincing. Using microfluidics and automated light microscopy, the authors made significant technical advancements in determining the lifespan of gametes from budding yeast in a high-throughput fashion.

With this technique, the authors confirmed some of their previous work, demonstrating that gametogenesis is a process that resets the replicative lifespan. They further validated their method using known factors, Sir2 and Fob1, which regulate the replicative lifespan.

Additionally, the authors discovered that Atg30 and Atg40, which are involved in ER-phagy, appear to be crucial for resetting the lifespan of gametes. Therefore, selective autophagy likely plays a crucial role in resetting the lifespan of gametes. In my view, this work will advance the field and provide a powerful tool for dissecting the mechanisms of gamete rejuvenation.

The authors used genetic tricks to isolate gametes that are suitable for microfluidic analysis. They observed that SIR2 deletion shortens lifespan as expected, but it is intriguing that meiotic SIR2 expression partially rescues the replicative lifespan in young but not old cells undergoing meiosis. This suggests that meiotically produced Sir2 can reset the replicative lifespan of gametes, but the underlying mechanism remains unclear.

Similarly, the involvement of ER-phagy in resetting lifespan alone does not fully explain the mechanism of gamete rejuvenation.

Given these intriguing observations, it is unclear what the connection is between selective autophagy and Sir2, if there is any.

Minor issues:
1. Since the gametes trapped in the microfluidics have already been incubated in a rich medium for 3 hours, how do the authors determine whether these cells have undergone cell division?
2. What is the logic behind expressing meiotic SIR2 and ATG40 with different promoters, RIM4 and NDT80, respectively?
3. In Fig 6E, if the authors intend to demonstrate autophagy, it would be more appropriate to compare the ratio of GFP to Sec63-GFP.

Reviewer #3

(Remarks to the Author)
This manuscript by Spiri et al. aims to address the long-standing question of how replicative lifespan is reset during gametogenesis in Saccharomyces cerevisiae. The authors develop an assay that enables quantitative measurement of replicative lifespan in individual yeast gametes.

Using this system, they confirm that gametogenesis fully resets replicative lifespan even in aged precursor cells. Among the central biological findings are: a. selective autophagy is required for gametogenic rejuvenation; b. mitophagy is dispensable; c. ER-phagy is essential; d. demonstration of a temporally specific requirement for ER-phagy during gametogenesis.

Overall, the study positions ER quality control via selective autophagy as a key determinant of germline rejuvenation, while introducing a platform for systematic discovery.

Major strengths:
While there are still concerns about the reported lifespan outcomes (see below), if further validated, the manuscript addresses a fundamental question in aging biology — how cellular age is reset across generations — and identifies ER-phagy as a specific mechanistic driver. This is a significant conceptual refinement beyond prior general links between autophagy and rejuvenation.

Major weaknesses:

1. Mechanistic depth is limited: While ER-phagy is identified as necessary, the downstream mechanism remains largely descriptive. What specific ER-associated damage is being cleared? Is the phenotype due to proteostasis failure, lipid

imbalance, or ER stress? The manuscript does not directly link ER turnover to a molecular aging phenotype.

2. Lack of direct aging biomarkers: The conclusions rely almost entirely on replicative lifespan as a readout. There is no direct demonstration that protein aggregates are cleared, or ER stress markers are reduced, or damaged ER is selectively excluded from gametes.

3. Potential confounding factors associated with the microfluidics workflow: certain steps could introduce bias, including 3-hour regrowth step before loading and selection of cells that successfully load into traps. Although some controls are presented, it is not fully demonstrated that these steps do not select for fitter gametes.

4. Misplaced novelty claim: while the authors wrote their manuscript around the claim of novelty of using a microfluidic platform for their assay, it's not the microfluidics setup (which is a commercial platform not optimized for these types cells with different ploidy). The assay development part is the novel part including Pma2-GFP being shown as a specific and retained marker of gametes exhibiting gamete-specific retention beyond germination, introduction of certain mutations to prevent sticking/flocculation, prevention of unwanted mating and the use of zymolase to weaken the ascus. While none of these modification were unknown, the authors used these known modifications smartly. These are the novel aspects and the assay that combines all these, not the obvious microfludics part. The authors need to edit their claims properly to reflect the real "technologcial" novelties.

5. For older cells, the entrapped cell is pulled out of the trap by its daughter cells that are not detaching properly after cytokinesis, cutting short the otherwise full lifepan of the mother cell under observation. This is ackowledged by the authors but what if this "daisy chaining" or improper cytokinesis is affected by the meiosis outcome, which would introduce a bias? For a study that heavily relies on lifespan measurements, inaccuracy in lifespan reporting could make the biological results questionable.

6. All cells seem to die after 32 generations with the average lifespan being around 22 generations. SK1 is not a mainstream s. cereviseae strain and it is not clear what is the expected average lifespan of SK1 cells. Also keep in mind that the SK1 cells used by the authors are not wild type. One needs to validate the chip performance first to see if it accurately reports SK1 lifespan when sir2 and fob1 was intact and when either of them was missing.

7. The fact that this assay was developed in a non-cononical strain background (SK1) and in a non-wild type manner (Amn1, Flo8 and Ste5 are not wild type) is a major concern. >90% of the yeast aging field uses the BY4741/42 background. This makes cross-comparison and generalization of the results difficult. The authors should show that their assay also works for the BY genetic background. As BY cells do not flocculate, they would not need to delete Flo8, for example. If there are reasons why miosis/rejuvenation outcomes are not "ideal" in the By background, the authors show prove those experimentally. Weaker efficiency is not a major bottleneck for BY. Perhaps the authors have tried to implement this assay in the By background and things did not work out; if so, they should show their "negative" results clearly and comprehensively.

Minor weakness: While not claimed to be a main focus, but SIR2 analysis is underdeveloped. The conclusion that sir2Δ defects are "gametogenesis-independent" is plausible but not fully resolved. There is no direct measurement of rDNA instability or DNA damage transmission. The rescue strategy may not fully recapitulate endogenous regulation.

I would be happy to reevaluate the manuscript after the above points are sufficiently addressed by the authors.

Version 1:

Reviewer comments:

Reviewer #1

(Remarks to the Author)
The revised manuscript fully satisfies all of the concerns I raised in my review.

Reviewer #2

(Remarks to the Author)
The authors have addressed my concerns, and I have no further questions.

Reviewer #3

(Remarks to the Author)
The authors sufficiently addressed my comments and I warmly recommend the publication of this revised work at Nat Comm.

Open Access This Peer Review File is licensed under a Creative Commons Attribution 4.0 International License, which permits use, sharing, adaptation, distribution and reproduction in any medium or format, as long as you give appropriate credit to the original author(s) and the source, provide a link to the Creative Commons license, and indicate if changes were made.
In cases where reviewers are anonymous, credit should be given to 'Anonymous Referee' and the source.
The images or other third party material in this Peer Review File are included in the article's Creative Commons license, unless indicated otherwise in a credit line to the material. If material is not included in the article's Creative Commons license and your intended use is not permitted by statutory regulation or exceeds the permitted use, you will need to obtain permission directly from the copyright holder.
To view a copy of this license, visit https://creativecommons.org/licenses/by/4.0/

Reviewer #1 (Remarks to the Author)

This very interesting paper by Spiri et al. primarily describes a new technique for measuring the reset of replicative lifespan in budding yeast after meiosis: microfluidic trapping of individual gametes for observation by microscopy. Although microfluidics had been previously used for measuring replicative lifespan in yeast during vegetative growth, it had not been applied to gametes after meiosis. The application of microfluidics allowed the authors much higher throughput than had been achieved with previous methods that required micromanipulation of the gametes. Using microfluidics, they were able to verify the previously reported reset of replicative lifespan by meiosis with a larger sample size. In addition, they were able for the first time to identify some of the factors required for this reset: the ER-phagy receptors Atg39 and Atg40, along with the selective autophagy adaptor Atg11. This is an exciting finding that also demonstrates the power of this approach.

This appears to be a very rigorous study, whose conclusions are well-supported by clear and conclusive data. The cell-sorting techniques used to separate young from old gametes are thoroughly validated and very efficient, and quality-control data about the cell populations (e.g. efficiency of gametogenesis and assessment of average population age via bud-scar counting) are presented for every experiment in the supplemental data. The methods are thorough and well-described, including the key genetic and experimental manipulations that were necessary to apply microfluidics techniques to the study of meiosis.

We are thrilled by the reviewer's enthusiasm for our work and greatly appreciate the time and thought that they dedicated to assessing this manuscript!

There are only a few things I would like the authors to address before I am ready to recommend this for publication:

1) In figure 6F, the accumulation of the Sec63-GFP is very subtle to see in the fluorescent microcopy images, and indeed the naked eye is not very good at judging the brightness of fluorescent images. To better support the conclusion that "Sec63-GFP accumulates during gametogenesis in young and aged cells lacking atg39Δ atg40Δ", please quantitate the average Sec63-GFP signal intensity in these strains and include that data as a graph in figure 6.

We thank the reviewer for this great suggestion and agree, quantification of Sec63-GFP signal is needed to robustly support our conclusion "Sec63-GFP accumulates during gametogenesis in young and aged cells lacking *atg39Δ atg40Δ*". To this end average GFP intensity was quantified as mean fluorescence after subtraction of image specific background signal. The quantification was added as new graph (**Fig. 6G**) and our method section and figure text was updated accordingly (**line 626-632, 663-664 & 837-839**).

2) Also in figure 6, but in panel E: The authors have very nicely included both the Sec63-GFP and the free GFP signal on their blot. As they are presumably aware, the level of free GFP is a readout of ER-phagy. There appears to be less free GFP in the atg39Δ/atg40Δ mutant, consistent with the expected defect in ER-phagy. This is a nice control supporting their interpretation of these mutants, but does not seem to be mentioned. I would recommend adding a sentence or two

to the results and to the figure legend making note of this. It would also be nice to see the quantification of free GFP/Hxk2 in their blots to further support this – this would ideally go in Figure 6 if space can be found, or else in a supplemental figure.

We appreciate this valuable feedback by the reviewer and agree that free-GFP quantification as readout for ER-phagy serves as a control to support our interpretation of these mutants. Therefore, as suggested by the reviewer we included quantification of free-GFP normalized to loading control (**Fig. 6E**, bar graphs in grey) and added a sentence in the result section and figure text to clarify this (**line 423-425 & 829-833**). In addition, we also included quantification of Free-GFP normalized to total GFP (Free-GFP+Sec63-GFP) as supplementary figure (Supplementary **Fig. 10E**) and updated the figure text (Supplementary information **line 340-344**).

3) Although the methods were generally quite thorough, one thing I could not find was what dye was used for staining the bud scars, or a cited reference for this procedure. Please include this in the methods (perhaps after line 576), and in the legend for figure 2D (line 715)

We are grateful to the reviewer for pointing out this missing information. We updated our methods section to clarify which dyes where used to stain bud scars and biotinylated cells respectively (**line 551-554**) additionally we also included the information in figure legend **2D** (**line 706-709**)

4) Here are a few typos or presentation issues that I noticed that should be fixed:

a. In the supplemental data, the yellow line graphs with white background are very hard to read (e.g. Figure S1A and S4E, and others). Please change the yellow to a darker color, such as orange or brown, for more contrast in every place where this occurs.

b. Similarly, when either yellow or especially cyan are used in the microscopy images, the colored label on the gray background (e.g. “Biotin” and “Pma2” in Figure 2D) is difficult to read. The color of the actual microscopy images doesn’t need to be changed, just the color of the label itself (or perhaps outline it in black of something of that sort to make it more visible).

c. There is a typo in figure S6L: the graph title says “Atg32”, but the figure legend indicates this is for Atg33.

d. Typo in line 392: The reference should be to Fig. S9G-K, not S8G-K.

e. Typo in line 836 – should be “from,” not “form”

f. In line 282, it would be better to say “vacuolar degradation”, since yeast have vacuoles instead of lysosomes. (Figure 5D is clear in this regard, so just the text needs to be updated.)

g. In line 322, no references are cited regarding Atg33 and its role in mitophagy. Please cite an appropriate paper here.

We thank the reviewer for such a careful assessment of our manuscript. We have adopted all the recommended changes in wording and color and fixed the typos (**line 273-275**). Additionally, we added the missing citation for Atg33 and its role in mitophagy (**line 314**) citing Kanki et al.,2009a[1].

Reviewer #2 (Remarks to the Author)

This manuscript is well written. The experimental procedures are well controlled and documented, and the data interpretation is convincing. Using microfluidics and automated light microscopy, the authors made significant technical advancements in determining the lifespan of gametes from budding yeast in a high-throughput fashion.

With this technique, the authors confirmed some of their previous work, demonstrating that gametogenesis is a process that resets the replicative lifespan. They further validated their method using known factors, Sir2 and Fob1, which regulate the replicative lifespan.

Additionally, the authors discovered that Atg30 and Atg40, which are involved in ER-phagy, appear to be crucial for resetting the lifespan of gametes. Therefore, selective autophagy likely plays a crucial role in resetting the lifespan of gametes. In my view, this work will advance the field and provide a powerful tool for dissecting the mechanisms of gamete rejuvenation.

The authors used genetic tricks to isolate gametes that are suitable for microfluidic analysis. They observed that SIR2 deletion shortens lifespan as expected, but it is intriguing that meiotic SIR2 expression partially rescues the replicative lifespan in young but not old cells undergoing meiosis. This suggests that meiotically produced Sir2 can reset the replicative lifespan of gametes, but the underlying mechanism remains unclear.

Similarly, the involvement of ER-phagy in resetting lifespan alone does not fully explain the mechanism of gamete rejuvenation.

Given these intriguing observations, it is unclear what the connection is between selective autophagy and Sir2, if there is any.

We thank the reviewer for their thoughtful assessment of our manuscript! We agree that ER-phagy does not fully explain gamete rejuvenation, which is expected given the complexity of proteostatic changes associated with aging. Nonetheless, we are excited to report the first gametogenic rejuvenation factors and to use our new approach to identify the other factors involved. We share the reviewer's interest in the surprising Sir2 result. Although this factor is established as relevant to aging in some contexts, a result that we recapitulate, it does not seem to be involved in the rejuvenation that accompanies gametogenesis. Rather, its loss seems to impact the fitness of cells prior to meiosis. As we agree that this set of results is interesting, we have expanded our discussion of it in the paragraph beginning at line 464 of the revised manuscript

Minor issues:

1. Since the gametes trapped in the microfluidics have already been incubated in a rich medium for 3 hours, how do the authors determine whether these cells have undergone cell division?

We are grateful for this question and the opportunity to clarify this important point with an additional figure (Supplementary **Fig. 2H**). During assay development we carefully monitored re-growth and determined the ideal timing to load and trap gametes to be when most of them had a chance to enlarge in size, but few had already completed their first division. We found that this "sweet spot" is at three hours in rich media (Supplementary **Fig. 2H**). At this time, almost all gametes are enlarged, thus decreasing the risk of any loading biases leading to loading only of the "fitter" gametes that initiated regrowth faster. On the other hand, to avoid gross underestimation of replicative lifespans, we chose a time when only a very small fraction of gametes had already completed their first division, which fortunately is also true of the three hour span. For every experiment included in this manuscript we kept the three hour re-growth time constant to exclude experiment to experiment variability. We tested several additional genotypes used in this study to show robustness of the "sweet spot" for the tested genotypes **(Revision Fig. 1A-D)**. Although, a small fraction of gametes possibly already completed their first cell division when time-laps imaging starts, given our robust results over many experiments we can exclude that this interferes with interpretation of gamete lifespan experiments. To illustrate this point better, as we agree that it is important, we added Supplementary **Figure 2H,** modified a sentence in the result section and added figure text (**line 180-183 &** Supplementary information **line 61-64**).

2. What is the logic behind expressing meiotic SIR2 and ATG40 with different promoters, RIM4 and NDT80, respectively?

For each rescue construct we choose promoter fragments whose transcription pattern closest resemble the pattern of the gene that was intended to be rescued during meiosis. *SIR2* expression levels peak early in meiosis during meiotic entry and decrease gradually during gametogenesis (Supplementary **Fig. 4F-H**). *RIM4* transcription closely resembles this pattern (compare Supplementary **Fig. E** to **F**), thus we choose RIM4 promoter fragment to drive SIR2 rescue during meiosis. *ATG40* expression levels on the other hand peak later around 6h in meiosis (Supplementary **Fig. 7D-F**) a pattern that is closer resembled by *NDT80* transcription levels (Supplementary **Fig. 9A** compared to Supplementary **Fig. 7D**). We added additional supplementary figures (Supplementary **Fig. 4F-H**) to illustrate *SIR2* transcription, translation, and protein levels during meiosis and clarified the logic behind promoter fragment selection in the result section (**line 253-256, 370-371 &** Supplementary information **line 123-129**).

3. In Fig 6E, if the authors intend to demonstrate autophagy, it would be more appropriate to compare the ratio of GFP to Sec63-GFP.

We thank the reviewer for this valuable feedback and agree that quantification of GFP ("Free-GFP") is more appropriate to demonstrate autophagy. Therefore, we compared the ration of Free-GFP to total GFP (Free-GFP+Sec63-GFP) and included a graph visualizing this quantification as supplementary figure (Supplementary **Fig. 10E**). In them main figure (**Fig. 6E**) we added quantification of free-GFP normalized to loading control (bar graphs in gray) in

addition to the pre-existing quantification of Sec63-GFP (bar graphs in cyan & magenta) and updated the result section and figure text for clarification (**line 293-296, 829-833 &** Supplementary information **line 340-343**).

Reviewer #3 (Remarks to the Author):

This manuscript by Spiri et al. aims to address the long-standing question of how replicative lifespan is reset during gametogenesis in Saccharomyces cerevisiae. The authors develop an assay that enables quantitative measurement of replicative lifespan in individual yeast gametes.

Using this system, they confirm that gametogenesis fully resets replicative lifespan even in aged precursor cells. Among the central biological findings are: a. selective autophagy is required for gametogenic rejuvenation; b. mitophagy is dispensable; c. ER-phagy is essential; d. demonstration of a temporally specific requirement for ER-phagy during gametogenesis.

Overall, the study positions ER quality control via selective autophagy as a key determinant of germline rejuvenation, while introducing a platform for systematic discovery.

Major strengths:
While there are still concerns about the reported lifespan outcomes (see below), if further validated, the manuscript addresses a fundamental question in aging biology — how cellular age is reset across generations — and identifies ER-phagy as a specific mechanistic driver. This is a significant conceptual refinement beyond prior general links between autophagy and rejuvenation.

We thank the reviewer for their thoughtful assessment of our manuscript and are delighted that they view our work as a significant addition to the body of work focused on explaining the factors that contribute to cellular aging.

Major weaknesses:

1. Mechanistic depth is limited: While ER-phagy is identified as necessary, the downstream mechanism remains largely descriptive. What specific ER-associated damage is being cleared? Is the phenotype due to proteostasis failure, lipid imbalance, or ER stress? The manuscript does not directly link ER turnover to a molecular aging phenotype.

We absolutely share the reviewer's interest in gaining a better molecular understanding for the role of ER-phagy in rejuvenating aged cells during gametogenesis. We agree, determining whether the observed rejuvenation phenotype is due to the failure to clear specific ER associated damage and what sort of damage might be targeted during this process as necessary to determine molecular mechanisms of ER-phagy during gametogenic rejuvenation and as an important major future research direction. However, such experiments may require extensive additional experimentation, and after discussing further with the editor, we believe that this would fall beyond the scope of the present study, which is focused on establishing and validating a platform to discover factors involved in gametogenic rejuvenation rather than elucidating downstream

molecular mechanisms. Importantly, we present robust evidence for our central conclusion that selective ER-phagy is necessary for gametogenic rejuvenation, which does not rely on the proposed experiments. We adapted our discussion (**line 463-465**) to clarify the scope of our conclusion and to point out this important future direction more clearly.

2. Lack of direct aging biomarkers: The conclusions rely almost entirely on replicative lifespan as a readout. There is no direct demonstration that protein aggregates are cleared, or ER stress markers are reduced, or damaged ER is selectively excluded from gametes,

We agree that characterizing the faith of specific aging markers during gametogenic rejuvenation is a valuable tool that provides relevant mechanistic insights for specific forms of cellular damage. Clearance of aging biomarkers during yeast gametogenesis has been established and demonstrated before with a protein aggregate marker for nuclear and cytosolic proteins (Hsp104-GFP) and a marker for extrachromosomal rDNA circles in the nucleus[2]. Unfortunately, these well-established age-associated markers report only on nuclear and cytosolic age associated damage while an established and broadly accepted ER specific aging marker is currently lacking for our system. Based on the core discoveries of this study, we are now focusing on developing such markers and are actively interrogating the molecular consequences of lacking ER-phagy during gametogenic rejuvenation through new purification and mass spectrometry approaches. We anticipate that this will yield exciting mechanistic insights in the future but will likely take years to accomplish. Based on this, and conferral with the editor, we believe this is a very exciting future direction but beyond the scope of this manuscript, the primary objective of which is to establish a robust experimental approach to interrogate replicative lifespan as the integrated direct physiological outcome of gametogenic rejuvenation. The core discovery here that ER-phagy is necessary for this makes the reviewer's suggestion a clear and important goal for our future work.

3. Potential confounding factors associated with the microfluidics workflow: certain steps could introduce bias, including 3-hour regrowth step before loading and selection of cells that successfully load into traps. Although some controls are presented, it is not fully demonstrated that these steps do not select for fitter gametes.

We thank the reviewer for pointing this out and for the opportunity to mitigate concerns about possible biases associated with our workflow and apologize that we did not fully explain this issue in the original manuscript. When developing this assay, we carefully monitored re-growth, as we shared the reviewer's exact concern about possible biases that could be introduced by this necessary step. We set out to find if there was a time when most of the gametes were sufficiently enlarged following regrowth, but few had already completed their first division. Such a time window would be ideal to allow loading and trapping of gametes while minimizing introduction of possible biases. We were thrilled to find that such a "sweet spot" did exist, and this was the basis for our choice of three hours of regrowth in rich media. We added an additional supplementary figure to the revised manuscript to illustrate this point better (Supplementary **Fig. 2H**) and adopted result and figure text accordingly (**line 180-183 & 61-64**). We show that at three hours almost all gametes are enlarged, thus decreasing the risk of bias leading to loading of only "fitter" gametes that initiated re-growth faster. For every experiment included in this manuscript we kept the three our re-growth time constant to avoid experiment to experiment

variability. To further confirm robustness of this timing across genotypes we monitored re-growth in different deletion backgrounds and found the three hour "sweet spot" to be robust for all tested genotypes **(Revision Fig. 1A-D)**. In addition, we monitored general gamete viability in these genotypes via manual microdissection of tetrads and confirmed that there are no viability defects excluding possible cofounding biases for the tested genotypes **(Revision Fig. 1E)**.

Revision Figure 1

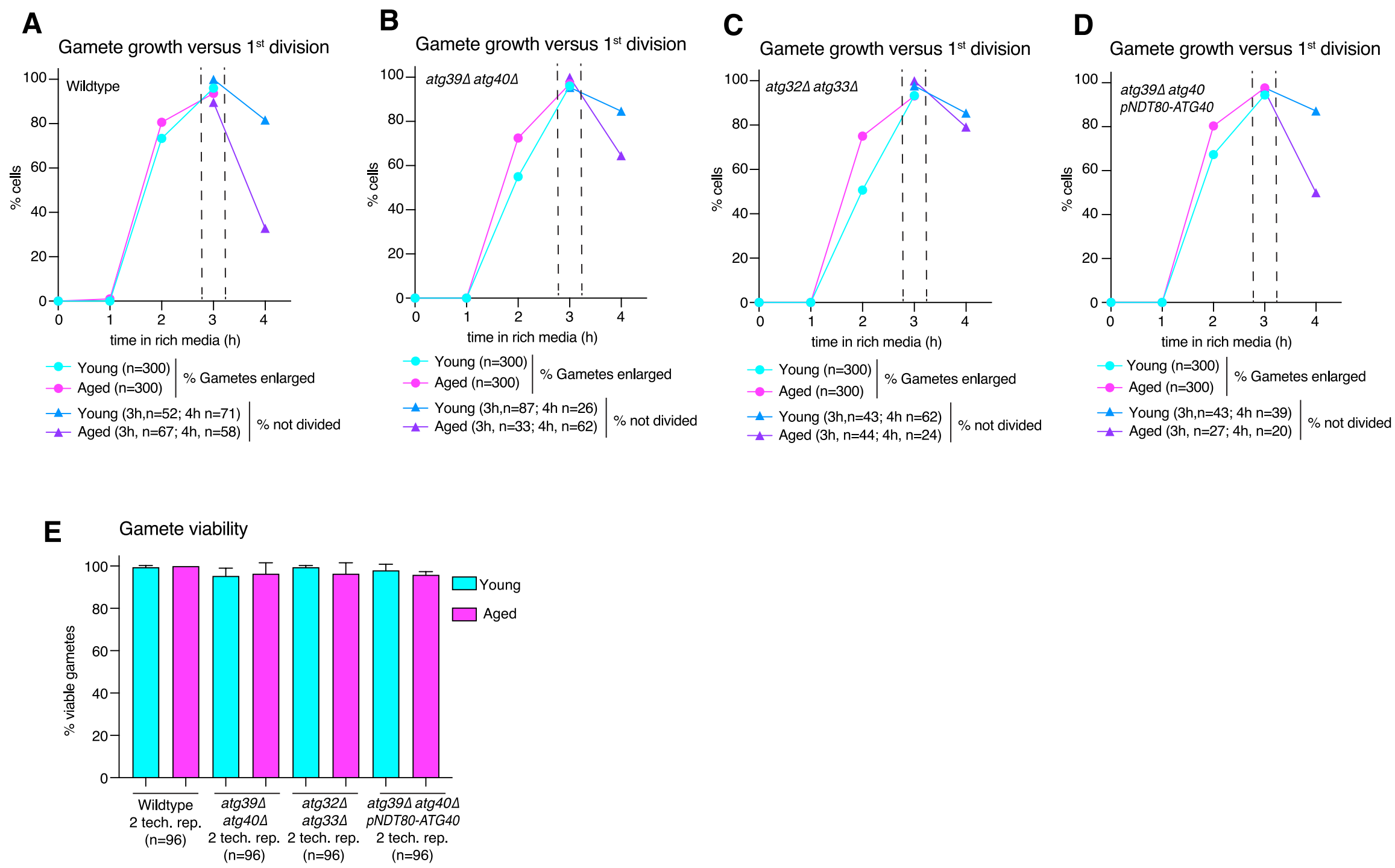

Revision Figure 1. Gamete regrowth and health: (**A-D**) Monitoring of growth and cell divisions of separated gametes in glucose containing liquid culture by scoring enlarged gametes (graphs depicted with dots) and assessing number of bud scars (graphs depicted with triangles).
A Growth and cell divisions of separated wildtype gametes.
B Growth and cell divisions of separated *atg39Δ atg40Δ* gametes.
C Growth and cell divisions of separated *atg32Δ atg33Δ* gametes.
D Growth and cell divisions of separated *atg39Δ atg40Δ pNDT80-ATG40* gametes.
C Quantification of gamete viability for young and aged wildtype, *atg39Δ atg40Δ, atg32Δ atg33Δ* mutants and *atg39Δ atg40Δ pNDT80-ATG40* mutants, showing percentage of gametes that form visible colonies (mean ± SD, 2 technical replicas n=96 each).

4. Misplaced novelty claim: while the authors wrote their manuscript around the claim of novelty of using a microfluidic platform for their assay, it's not the microfluidics setup (which is a commercial platform not optimized for these types cells with different ploidy). The assay development part is the novel part including Pma2-GFP being shown as a specific and retained marker of gametes exhibiting gamete-specific retention beyond germination, introduction of certain mutations to prevent sticking/flocculation, prevention of unwanted mating and the use of zymolase to weaken the ascus. While none of these modification were unknown, the authors

used these known modifications smartly. These are the novel aspects and the assay that combines all these, not the obvious microfludics part. The authors need to edit their claims properly to reflect the real "technologcial" novelties.

We agree with the reviewer and thank them for raising this point giving us the opportunity to correct any confusion caused by our initial wording. We edited the wording throughout the revised manuscript accordingly to clarify this point and to avoid any misplaced novelty claims. To assure readers do not get mislead to think we developed the adapted but previously established microfluidic devices we carefully changed our wording throughout the manuscript (**lines: 2, 25-26, 72, 84, 147, 179 & 414**).

5. For older cells, the entrapped cell is pulled out of the trap by its daughter cells that are not detaching properly after cytokinesis, cutting short the otherwise full lifepan of the mother cell under observation. This is ackowledged by the authors but what if this "daisy chaining" or improper cytokinesis is affected by the meiosis outcome, which would introduce a bias? For a study that heavily relies on lifespan measurements, inaccuracy in lifespan reporting could make the biological results questionable.

We thank the reviewer for raising this important point, that genotype dependent "daisy-chaining" induced cell ejection could bias lifespan quantification. All strains used in this study carried an inert point mutation at the *AMN1* locus that promotes efficient mother-daughter cell separation, reducing "daisy-chaining"[3]. Premature cell ejection still occurs during long-term timelapse experiments; therefore, if we did not observe a terminal phenotype, cells were censored rigorously. As mentioned by the reviewer, we acknowledge and report censor rates for each experiment in corresponding figure legends. To exclude any possible biases towards certain genotypes or experimental groups, we plotted the percentage of all censored cells across all replicative lifespan experiments included in this manuscript, as reported in each figure legend. We found that censoring rates are comparable between genotypes (**Revision Figure 2.** censored subjects=62%± 8.4, mean ± SD), with no systematic relationship between ejection frequency and observed lifespan phenotypes. Thus, although cell ejection represents a substantial source of censoring inherent to the assay, partly because we count every loaded gamete even if they eject within the first few divisions, we see no evidence that differential ejection between genotypes accounts for the reported lifespan differences.

Revision Figure 2

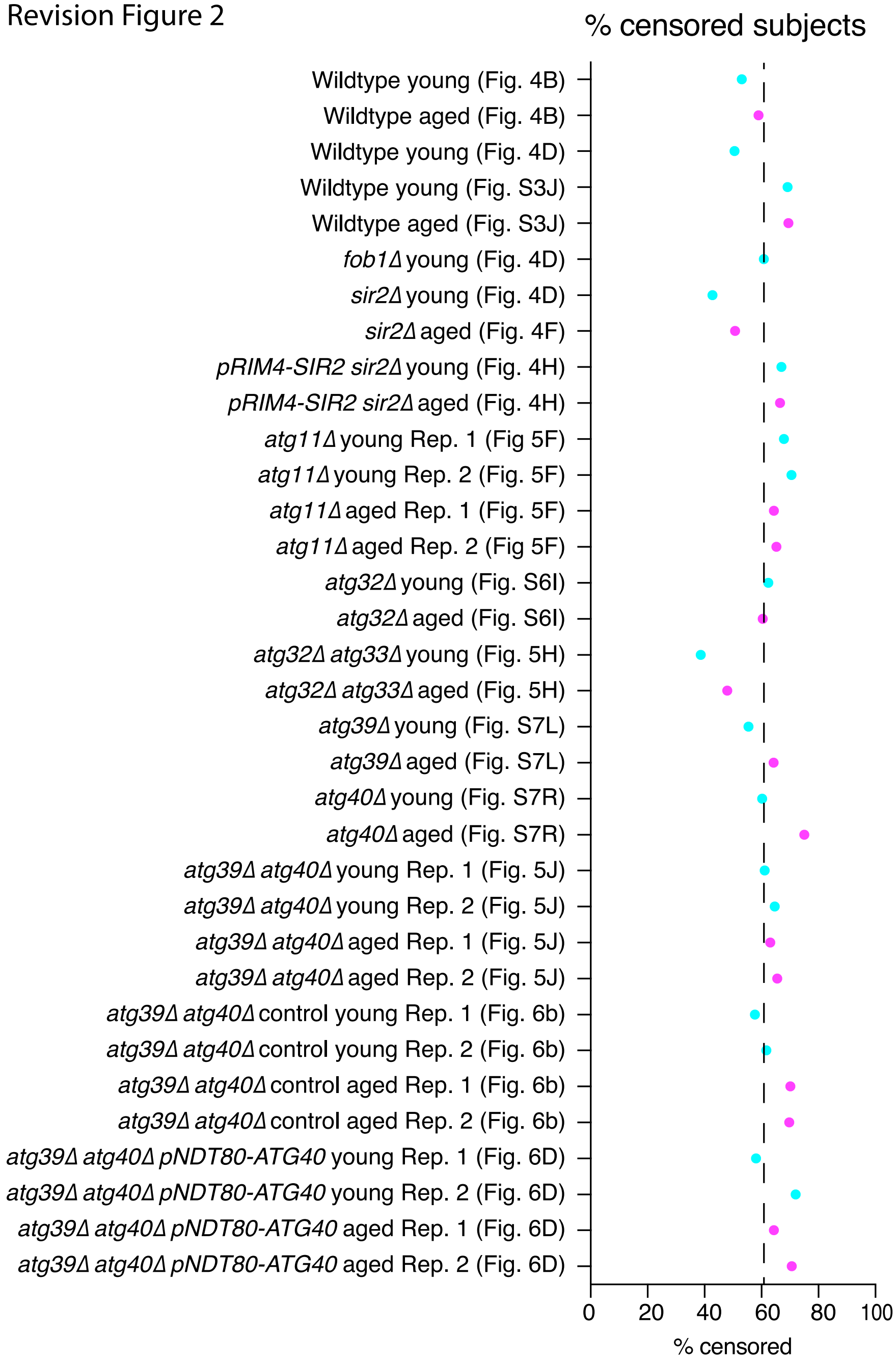

Revision Figure 2. Percentage of censored events over all lifespan experiments: Reported censored subjects from all lifespan experiments compiled. Dashed line indicates mean (62%± 8.4, mean ± SD) of censored subjects from n=34 experiments.

6. All cells seem to die after 32 generations with the average lifespan being around 22 generations. SK1 is not a mainstream s. cereviseae strain and it is not clear what is the expected average lifespan of SK1 cells. Also keep in mind that the SK1 cells used by the authors are not wild type. One needs to validate the chip performance first to see if it accurately reports SK1 lifespan when sir2 and fob1 was intact and when either of them was missing.

Although there are several lifespan experiments where the longest-lived cell recorded falls around 30-35 divisions, we are uncertain why the reviewer believes that all cells die after 32 generations. There are several lifespan experiments with cells that complete more than 32 divisions before reaching senescent, with up to 52 divisions in **Fig. 4D** (**Fig. 4B**, **Fig. 4D**, **Fig. 4H**, Supplementary **Fig. 6I**, Supplementary **Fig. 7L**, Supplementary **Fig. 7R**, **Fig. 6B**, **Fig. 6D**).

That said, we strongly agree with the reviewer that it is important to validate chip performance and to make sure that our system accurately captures lifespan dynamics. To this end, we compared *sir2*Δ, wildtype and *fob1*Δ lifespan measurement using our setup and genetically modified background (**Fig. 4D** and **Revision Fig. 3A**) with published data from studies in which lifespan profiling was performed via microdissection in SK1 and W303R backgrounds respectively (**Revision Fig. 3B-3D and** Supplementary **Table. 1**). The results obtained with our setup are highly comparable to published results. The degree of similarity is actually remarkable given that lifespan is highly context dependent and can vary between measurement methods[4]. Thus, the fact that the dynamic range of lifespan measurements between these known long- and short-lived mutants are so similar across measurements methods and strain backgrounds gives us confidence in the approach we applied and trust that this strategy accurately reports SK1 gamete lifespan in this context. To make this important point clearer, we added a new supplementary table (attached below) (Supplementary **Table. 1,** Supplementary information **line 345**) and edited the text in the result section (**line 232-236**).

Revision Figure 3

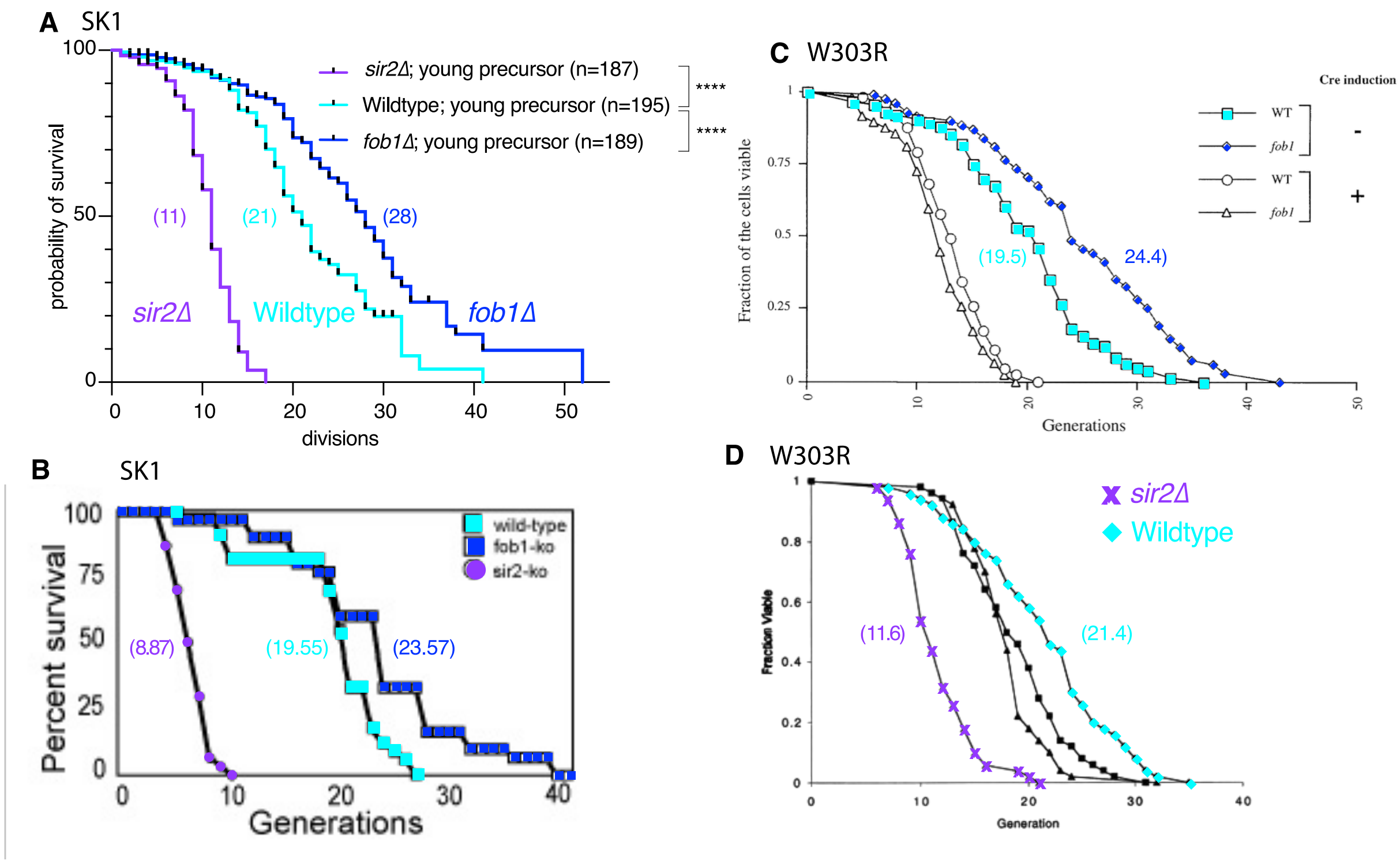

Revision Figure 3. Comparison of previously reported microdissection-based lifespans of known long and short-lived mutants in SK1 and W303R.

A Data from this study (**Fig. 4D**). Lifespan measurements acquired with SK1 gametes from young precursor cells using microfluidics-based lifespan profiling. Quantification of replicative lifespan of young *sir2*Δ (median lifespan=11, n=187, 80 censored subjects, p<0.0001 compared to wildtype), wildtype (median lifespan=21, n=195, 103 censored subjects) and *fob1*Δ (median lifespan=28, n=286, 174 censored subjects, p<0.0001 compared to wildtype) gametes.

B Data and graph from Boselli et al. 2009 (adapted from Fig. S7A[5]). Lifespan measurements acquired with SK1 haploid cells using micromanipulation-based lifespan profiling. Quantification of replicative lifespan in *sir2*Δ (lifespan=8.87±1.8, mean±SD, n=30), wildtype (lifespan=19.55=±5.53, mean±SD, n=33) and *fob1*Δ (lifespan=23.57±7.91, mean±SD, n=30).

C Data and Graph from Defossez et al. 1999 (adapted from Fig. 4[6]). Lifespan measurements acquired with W303R haploid cells using micromanipulation-based lifespan profiling. Quantification of replicative lifespan in *fob1*Δ (mean lifespan=24.4, n=40-50), wildtype (mean lifespan=19.5, n=40-50).

D Data and graph from Kaeberlein et al. 1999 (adapted from Fig. 1A[7]. Lifespan measurements acquired with W303R haploid cells using micromanipulation-based lifespan profiling. Quantification of replicative lifespan in *sir2*Δ (mean lifespan=11.6, n=50), wildtype (mean lifespan=21.4, n=50).

Supplementary Table 1. Lifespan comparisons

Genotype	Strain background	Method	Mean Replicative lifespan	% Change relative to Wildtype	Reference
Wildtype	SK1	Microfluidics	21	0	This study
sir2Δ	SK1	Microfluidics	11	-47.6	This study
fob1Δ	SK1	Microfluidics	28	+33.3	This study
Wildtype	SK1	Microdissections	19.55	0	[8]
sir2Δ	SK1	Microdissections	8.87	-54.6	[8]
fob1Δ	SK1	Microdissections	23.57	+20.6	[8]
Wildtype	W303R	Microdissections	21.4	0	[7]
sir2Δ	W303R	Microdissections	11.6	-45.8	[7]
Wildtype	W303R	Microdissections	19.5	0	[6]
fob1Δ	W303R	Microdissections	24.4	+25.12	[6]

7. The fact that this assay was developed in a non-cononical strain background (SK1) and in a non-wild type manner (Amn1, Flo8 and Ste5 are not wild type) is a major concern. >90% of the yeast aging field uses the BY4741/42 background. This makes cross-comparison and generalization of the results difficult. The authors should show that their assay also works for the BY genetic background. As BY cells do not flocculate, they would not need to delete Flo8, for example. If there are reasons why miosis/rejuvenation outcomes are not "ideal" in the By background, the authors show prove those experimentally. Weaker efficiency is not a major bottleneck for BY. Perhaps the authors have tried to implement this assay in the By background and things did not work out; if so, they should show their "negative" results clearly and comprehensively.

We appreciate the reviewer's point about differences in phenotypes in some cases that varies with strain background. However, we believe SK1 is the ideal strain background to study gametogenic rejuvenation as it naturally initiates and executes gametogenesis reliably and with high efficiency. We have yet to observe any negative effects of the inert mutations introduced into the SK1 strain background (*flo8*Δ, *amn1*^{D368V} variant and constitutive *ste5*Δ). In fact, during the planning of this project, we were surprised to learn that both *flo8* and *amn1* modifications are copied from and present in the S288C strain background, the parental strain for BY4741/42. *FLO8*, the gene we deleted to decrease flocculation in SK1, is known to carry a loss of function mutation in S288C, possibly selected for reduced flocculation during laboratory use[9]. *AMN1,* on the other hand, is found to have an aspartate at position 368 in 34 of 46 investigated yeast strains including SK1. Most of the 12 strains that were found to have a valine at position 368, the exact modification we introduced into SK1 *(amn1*^{D368V}*),* had S288C as common ancestor (S288C, BY4741, BY4742, W303, FY1679, JK9-3d, X2180-1A, YPH499 and non S288C: CEN.PK113-7D, CEN.PK2-1Ca, FL100, SEY6210)[3]. The authors of that study concluded *amn1*^{D368V} to be the non-dominant from of *amn1* that has been selected for by humans during laboratory use as it

promotes efficient mother-daughter cell separation and thus decreases clumping and eases general handling. To our knowledge, there are no reports or evidence that suggests either *flo8*Δ, the *amn1*^{D368V} variant nor a constitutive *ste5*Δ modify replicative lifespan in yeast. Furthermore, we use the same engineered genetic background for all our experiments and are thus not concerned that the modifications necessary for our assay to work interfere with either lifespan or gametogenic rejuvenation outcomes.

We agree with the reviewer that adaption of the here presented rejuvenation assay into the BY4741/42 background would be valuable for the larger yeast community, therefore we undertook efforts to do so. We tested efficiency of gametogenesis in BY4741/42 wildtype diploids but found that only a very low percentage of cells formed tetrads (**Revision Fig. 4A**). Only 1% and 2% of cells at 24h and 72h after induction of gametogenesis respectively formed visible tetrads, which is known, and due to natural occurring polymorphisms affecting meiotic entry and progression in the BY4741/42 background[10,11]. Although the reviewer is right that our assay does not solely rely on high efficiency of gametogenesis to be successful, these very low numbers of formed tetrads presented a major bottleneck to collecting data that allows rigorous analyses. We can typically isolate around 10 OD_{600} Units of aged cells starting from 2x8 OD_{600} biotin-labeled founder cells. If only 1% of these cells form tetrads, that would give us 0.1 OD_{600} Units of tetrads. One concern is that the 99% of cells that did not form tetrads represent a large amount of biomass and could lead to problems during loading and increased risk of clumping if they are not completely degraded and removed during zymolyase treatment and downstream preparations. A second and more important concern is the number of cells we would be able to isolate would be prohibitively low to efficiently and reproducibly load the microfluidics devices with enough cells for robust analysis. We use at least 1 mL of cell suspension at OD_{600} 0.8 for loading of two imaging units to reach high enough loading efficiency to robustly determine replicative lifespan. Assuming we could isolate and separate 0.1 OD_{600} Units of tetrads efficiently, that would only give us 0.125 mL cell suspension at OD_{600} 0.8, an amount that would limit efficient loading. Thus, we would have to scale up isolation of aged cells at least 10-fold, which would pose significant logistical and cost challenges.

However, based on the reviewer's interest, we did attempt to analyze a BY-related background by turning to DHY213, a "repaired" BY-derived strain[12], carrying SK1-derived gametogenesis enhancing alleles (*MKT1*^{30G} *RME1*$^{INS-308A}$ *TAO3*^{1493Q}), together with alleles (*CAT5*^{91M} *SAL1+* *MIP*^{661T} *HAP1+*), which restore major mitochondrial defects of the standard BY/SC288C laboratory strain background, and thus complete gametogenesis with higher efficiency (**Table Revision 1. & Revision Fig. 4A**, 42% and 54.3% of cells 24h and 72h after induction of gametogenesis respectively)[10,12,13]. We introduced the necessary modifications to be able to perform the rejuvenation assay into DHY213 (**Table Revision 2 strain table**) and performed rejuvenation assay for two individually isolated diploids. We sorted diploid cells and analyzed replicative age of young and aged pre-gametogenic cell populations (**Revision Fig. 4B&C)**, analyzed efficiency of gametogenesis and determined age of post gametogenic tetrads (**Revision Fig. 4D-F)** before acquisition and quantification of gamete replicative lifespan. When quantifying the replicative lifespan of gametes, we found a significant lower replicative lifespan of young gametes compared to SK1 (median lifespan diploid 1= 17 and diploid 2=15). In addition and surprisingly, we found a significant difference between gametes from aged and

young DHY213 precursor cells in both diploids (**Revision Fig. 4G**, diploid 1, $p<0.0001$, diploid 2, $p<0.0001$).

There are, in principle, at least three possible interpretations for these surprising results using DHY213: 1. Gametogenic rejuvenation is not conserved across yeast strains; 2. DHY213 might contain modifications leading to accumulation of damage that severely reduce lifespan and cannot be rescued by the gametogenic rejuvenation program; and 3. One or more alleles introduced into DHY213 to improve efficiency of gametogenesis causes gametogenic rejuvenation to fail. Based on the body of existing data, we believe that this first explanation can be excluded, as gametogenic rejuvenation was discovered in W303, a laboratory strain commonly used in yeast aging research which is largely derived from S288C the parental line for BY4741/42[14]. Additionally, gametogenic rejuvenation was confirmed in A364a (Ünal et al., 2011), a strain related to S288C but more diverged than W303 and has now been verified again in SK1 a S288C independent strain background (this study).

There is reason to believe the second explanation could have merit. Although there is one study reporting similarly low replicative lifespan results using BY4741/42[15] as we observe for young gametes using DHY213, we found this to be on the lower spectrum of expected replicative lifespan for a BY4741/42 derivate[4] and to our knowledge there are no previous investigations of replicative lifespan in DHY213 specifically. Thus, we speculate that DHY213 might contain specific mutations that lead to accumulation of damage and severely reduce replicative lifespan which possibly impeded cellular rejuvenation during gametogenesis like we observed with *sir2*Δ (this study).

The third explanation could also be true and would be very exciting. If a specific modification introduced into DHY213 to improve efficiency of gametogenesis, or a DHY213-specific trait, impedes gametogenic rejuvenation, studying this could help to reveal additional mechanisms that are necessary for gametogenic rejuvenation (**Table Revision 1**). We are excited and grateful for this interesting and serendipitous lead.

Nonetheless, none of this allows us to perform gametogenic rejuvenation experiments in BY. We hope the reviewer understands why we believe that SK1 is the ideal background for our study, based on the efficiency of sporulation, recapitulation of results seen with microdissection in wild-type and mutant backgrounds shown in this manuscript, and our argument (above, based on the histories of both strains in laboratory use) that this strain is no less “wild-type” than BY.

Revision Figure 4

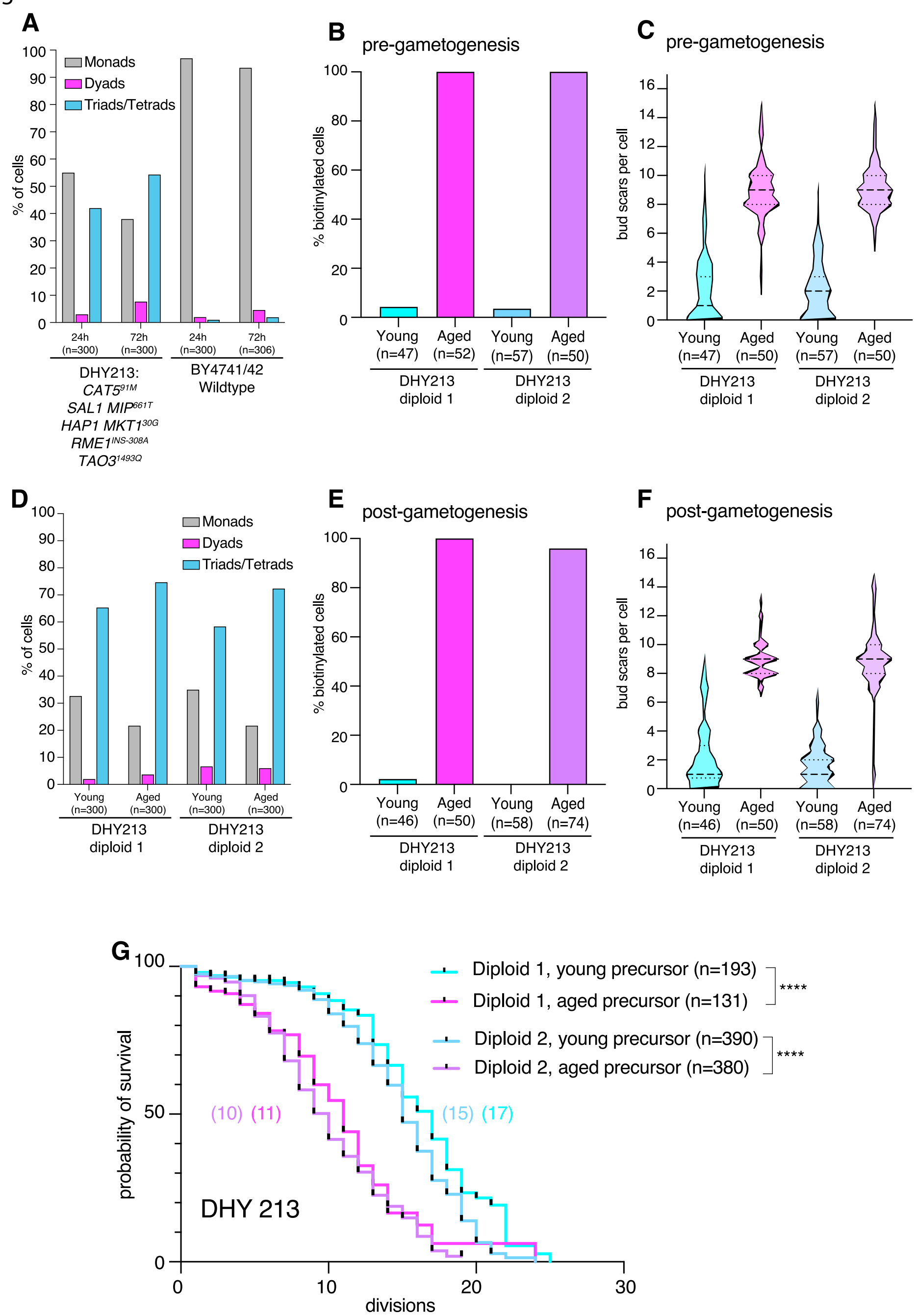

Revision Figure 4. Efforts to adopt SK1 based rejuvenation assay to a BY4741/42 derived background.

A Fraction of tetrads (cyan) in BY4741/42 wildtype and DHY213 at 24h and 72h post-transfer to gametogenesis inducing media. Fraction of tetrads in DHY213 at 24h (42.0%, 95% CI: 36.6–47.7%, n=300), BY "fixed" at 72h (54.3%, 95% CI: 48.7–59.9%, n=300), BY wildtype 24h (1.0%, 95% CI: 0.3–2.9%, n=300) and BY4741/42 wildtype 72h (2.0%, 95% CI: 0.9–4.3%, n=306).

B Fraction of biotinylated cells in DHY213 cell populations: diploid 1 young (4.3%, 95% CI: 1.2–14.2%, n=47) and aged (all, n=52); diploid 2 young (3.5%, 95% CI: 1–11.9%, n=57) and aged (all, n=50).

C Quantification of replicative age by assessing number of bud scars in DHY213 cell populations: diploid 1 young (2± 2.3, mean ± SD, n=47) and aged (8.9± 1.8, mean ± SD, n=50); diploid 2 young (1.9 ± 2, mean ± SD, n=57) and aged (9.1± 1.5, mean ± SD, n=50).
D Fraction of tetrads in post-gametogenic DHY213 cell populations: diploid 1 young (65.3%, 95% CI: 59.8–70.5%, n=300) and aged (74.7%, 95% CI: 69.5–79.3%, n=300); diploid 2 young (58.3%, 95% CI: 52.7–63.8%, n=300) and aged (72.3%, 95% CI: 67.0–77.1%, n=300).
E Fraction of biotinylated tetrads in DHY213 populations after completion of gametogenesis: diploid 1 young (2.2%, 95% CI: 0.4–11.3%, n=46) and aged (all, n=50); diploid 2 young (none, n=58) and aged (95.9%, 95% CI: 88.7–98.6%, n=74).
F Quantification of replicative age by assessing number of bud scars in DHY213 control tetrad populations: diploid 1 young (2.0± 2.1, mean ± SD, n=46) and aged (8.9± 1.2, mean ± SD, n=50); diploid 2 young (1.5± 1.4, mean ± SD, n=58) and aged (8.7± 2.3, mean ± SD, n=74).
G Quantification of replicative lifespan of DHY213 control gametes: diploid 1 young (median lifespan=17, n=193, 109 censored subjects, 1 technical replicate) and aged (median lifespan=11, n=131, 70 censored subjects, 1 technical replicate, p<0.0001 compared to young); diploid 2 young (median lifespan=15, n=390, 171 censored subjects) and aged (median lifespan=10, n=380, 191 censored subjects, p<0.0001 compared to young).

Table Revision 1. Modified alleles in DHY213 [12]

Allele	Function	BY/S288C defect	Effect of repair	Origin of repaired allele	Reference
MKT1^{30G}	Global post-transcriptional regulator	BY allele *MKT1^{30D}*, partial loss of function, altered global gene expression	QTL for sporulation, improves efficiency of gametogenesis	SK1 allele	[10,16]
RME1$^{INS-308A}$	Represses meiotic entry by repression of IME1	*RME1$^{del-308A}$* increased RME1 levels, reducing efficiency of meiotic entry	Lower RME1 transcription levels, lower repression of IME1 allows meiotic entry	SK1 allele	[10,17]
TAO3^{1493Q}	Component of RAM signaling network, involved in morphogenesis & sporulation	BY allele *TAO3^{1493E}* has lower sporulation efficiency	Metabolic remodeling necessary during early meiosis	SK1 allele	[10,18]
CAT5^{91M}	Coenzyme Q (ubiquinone) biosynthesis	Hypomorphic BY allele	Reduced petite frequency	Ancestral wildtype allele	[16]
SAL1+	Mitochondrial Ca^{2+} ATP-Mg/Pi transporter	Frameshift mutation	Reduced petite frequency	Ancestral wildtype allele	[16]

MIP^{661T}	Mitochondrial DNA polymerase gamma	BY allele *MIP*^{661A}, elevated mtDNA mutation rate	Reduced petite frequency	Ancestral wildtype allele	[16]
HAP1+	Oxygen and heme responsive transcription factor	3` Ty1 insertion	Regulation of gene expression	Ancestral wildtype allele	[12]

Minor weakness: While not claimed to be a main focus, but SIR2 analysis is underdeveloped. The conclusion that sir2Δ defects are "gametogenesis-independent" is plausible but not fully resolved. There is no direct measurement of rDNA instability or DNA damage transmission. The rescue strategy may not fully recapitulate endogenous regulation.

We agree with the reviewers point that we cannot guarantee that our rescue strategy fully recapitulates endogenous regulation. Even though we choose the *pRIM4* promoter fragment to drive *SIR2*, due to its similar expression pattern during meiosis, it is challenging to match expression levels precisely. We added additional supplementary figures (Supplementary **Fig. 4F-H**) to illustrate *SIR2* transcription, translation, and protein levels during meiosis and to show that *RIM4* transcription closely resembles the transcription pattern of *SIR2* (compare Supplementary **Fig. 4E** to **F**) and clarified this point in our result and discussion sections (**line 253-256 & 432-434**).
We fully agree that we do not present any experimental evidence to resolve how lack of Sir2 leads to failure of gametogenic rejuvenation. Therefore, we softened our language in the discussion and added a sentence about how this should be elucidated in future studies (**line 434-440**).

I would be happy to reevaluate the manuscript after the above points are sufficiently addressed by the authors.

References Revisions:

1. Kanki, T. *et al.* A Genomic Screen for Yeast Mutants Defective in Selective Mitochondria Autophagy. *Mol Biol Cell* **20**, 4730–4738 (2009).
2. King, G. A. *et al.* Meiotic cellular rejuvenation is coupled to nuclear remodeling in budding yeast. *eLife* **8**, e47156 (2019).
3. Fang, O. *et al.* Amn1 governs post-mitotic cell separation in Saccharomyces cerevisiae. *PLOS Genetics* **14**, e1007691 (2018).
4. Chen, K. L., Crane, M. M. & Kaeberlein, M. Microfluidic technologies for yeast replicative lifespan studies. *Mechanisms of Ageing and Development* **161**, 262–269 (2017).
5. Boselli, M., Rock, J., Ünal, E., Levine, S. S. & Amon, A. Effects of Age on Meiosis in Budding Yeast. *Developmental Cell* **16**, 844–855 (2009).
6. Defossez, P.-A. *et al.* Elimination of Replication Block Protein Fob1 Extends the Life Span of Yeast Mother Cells. *Molecular Cell* **3**, 447–455 (1999).
7. Kaeberlein, M., McVey, M. & Guarente, L. The SIR2/3/4 complex and SIR2 alone promote longevity in Saccharomyces cerevisiae by two different mechanisms. *Genes Dev* **13**, 2570–2580 (1999).
8. Boselli, M., Rock, J., Ünal, E., Levine, S. S. & Amon, A. Effects of Age on Meiosis in Budding Yeast. *Developmental Cell* **16**, 844–855 (2009).
9. Liu, H., Styles, C. A. & Fink, G. R. *Saccharomyces cerevisiae* S288C Has a Mutation in *FL08* , a Gene Required for Filamentous Growth. *Genetics* **144**, 967–978 (1996).
10. Deutschbauer, A. M. & Davis, R. W. Quantitative trait loci mapped to single-nucleotide resolution in yeast. *Nat Genet* **37**, 1333–1340 (2005).

11. Loll-Krippleber, R., Jiang, Y. K. & Brown, G. W. pSPObooster: A Plasmid System to Improve Sporulation Efficiency of Saccharomyces cerevisiae Lab Strains. *Yeast* **41**, 585–592 (2024).

12. Harvey, C. J. B. *et al.* HEx: A heterologous expression platform for the discovery of fungal natural products. *Sci Adv* **4**, eaar5459 (2018).

13. Dimitrov, L. N., Brem, R. B., Kruglyak, L. & Gottschling, D. E. Polymorphisms in Multiple Genes Contribute to the Spontaneous Mitochondrial Genome Instability of Saccharomyces cerevisiae S288C Strains. *Genetics* **183**, 365–383 (2009).

14. Ünal, E., Kinde, B. & Amon, A. Gametogenesis Eliminates Age-Induced Cellular Damage and Resets Life Span in Yeast. *Science* **332**, 1554–1557 (2011).

15. Salzman, V. *et al.* Reliable replicative lifespan determination of yeast with a single-channel microfluidic chip. *Biol Open* **13**, bio060596 (2024).

16. Dimitrov, L. N., Brem, R. B., Kruglyak, L. & Gottschling, D. E. Polymorphisms in Multiple Genes Contribute to the Spontaneous Mitochondrial Genome Instability of Saccharomyces cerevisiae S288C Strains. *Genetics* **183**, 365–383 (2009).

17. Bushkin, G. G. *et al.* m6A modification of a 3′ UTR site reduces RME1 mRNA levels to promote meiosis. *Nat Commun* **10**, 3414 (2019).

18. Gupta, S. *et al.* Meiotic Interactors of a Mitotic Gene TAO3 Revealed by Functional Analysis of its Rare Variant. *G3 (Bethesda)* **6**, 2255–2263 (2016).

Table Revisions 2. Strain Table

Strain number yÜB[1]	MAT	Genotype[2,3]	Source
BY	Strain background	*leu2Δ0 his3Δ1 ura3Δ0 met15Δ0*	
3412	*MAT**a***	*leu2Δ0 his3Δ1 ura3Δ0 met15Δ0*	
41485	*MAT**α***	*leu2Δ0 his3Δ1 ura3Δ0 lys2Δ0*	
49914	*MAT**a**/**α*** (diploid)	*lys2Δ0/+, met15Δ0/+*	
DHY213	Strain background	*leu2Δ0 his3Δ1 ura3Δ0 met15Δ0, MKT1*^{30G}, *RME1*$^{INS\text{-}308A}$, *TAO3*^{1493Q}, *CAT5*^{91M}, *SAL1+, MIP*^{661T}, *HAP1+*	[12]
32507	*MAT**a***	*leu2Δ0 his3Δ1 ura3Δ0 met15Δ0, MKT1*^{30G}, *RME1*$^{INS\text{-}308A}$, *TAO3*^{1493Q}, *CAT5*^{91M}, *SAL1+, MIP*^{661T}, *HAP1+*	[12]
32509	*MAT**α***	*leu2Δ0 his3Δ1 ura3Δ0 met15Δ0, MKT1*^{30G}, *RME1*$^{INS\text{-}308A}$, *TAO3*^{1493Q}, *CAT5*^{91M}, *SAL1+, MIP*^{661T}, *HAP1+*	[12]
50504	diploid	*leu2Δ0 his3Δ1 ura3Δ0 met15Δ0, MKT1*^{30G}, *RME1*$^{INS\text{-}308A}$, *TAO3*^{1493Q}, *CAT5*^{91M}, *SAL1+, MIP*^{661T}, *HAP1+, his3::pPma2-Pma2-eGFP::HIS3, ste5::NatR-pTet07.1-STE5, ura3::pRNR2-TetR-Tup1, pTetO7.1-TetR::URA3*	this study
50505	diploid	*leu2Δ0 his3Δ1 ura3Δ0 met15Δ0, MKT1*^{30G}, *RME1*$^{INS\text{-}308A}$, *TAO3*^{1493Q}, *CAT5*^{91M}, *SAL1+, MIP*^{661T}, *HAP1+, his3::pPma2-Pma2-eGFP::HIS3, ste5::NatR-pTet07.1-STE5, ura3::pRNR2-TetR-Tup1, pTetO7.1-TetR::URA3*	this study

[1]Strains are listed according to Figure order.
[2]Alleles are separated by commas.
[3]All indicated alleles in diploids are homozygous, unless otherwise specified.
